# Fractured systems: a literature review of OR/MS methods applied to orthopaedic care settings and treatments

**DOI:** 10.1080/20476965.2023.2264348

**Published:** 2023-10-09

**Authors:** Matthew Howells, Paul Harper, Geraint Palmer, Daniel Gartner

**Affiliations:** School of Mathematics, Cardiff University, Cardiff, UK

**Keywords:** Orthopaedics, literature review, operational research, management science

## Abstract

Orthopaedic systems are facing an impending wave of increased pressures as a result of global ageing populations. This is compounded by the current stresses these services face, as a result of the COVID-19 pandemic, and increasing burden of musculoskeletal conditions. It is vital that measures are taken to alleviate the pressures on these systems, to ensure timely and quality access to care for patients. This literature review presents a taxonomic classification of the applications of Operational Research and Management Science (OR/MS) methodologies to orthopaedic care settings and treatments, covering the general, medical, and methodological context of each paper. Our structured search identified 492 relevant publications that have been included in our analysis. The results found a literature largely dominated by cost analysis applications, typically utilising Markov models or decision trees. Key gaps identified in this review include the lack of holistic modelling of orthopaedic systems and pathways, and limited applications to resource and capacity planning. The implications of our review are that researchers, healthcare professionals and managers can develop a research agenda to address these gaps, and enhance decision support in orthopaedics.

## 1. Introduction

### 1.1. Background and motivation

Globally, the world faces an oncoming wave of intensified demand to many areas of healthcare delivery, as a result of rising ageing populations, with the World Health Organization (WHO) predicting that by 2030, one in six of the world’s population will be aged 60 years or older (World Health Organization, 2022). Already, in 2017, the number of people aged over 60 was double that of 1980 (United Nations, 2019). The ageing population will likely be accompanied by increased frailty, disability and dependency, creating increased demand for social and health services (Lunenfeld & Stratton, 2013).

The effect this will likely have on Trauma & Orthopaedics (T&O) is palpable. Due to their frailty and poorer bone density, this increasing population cohort finds themselves at a heightened risk and complexity of orthopaedic injury and trauma, such as fractures (Cummings & Melton, 2002; Johnell et al., 2005). Given the increased likelihood of comorbidities in older patients, it has also been found that these patients are at an elevated risk of post-surgical adverse effects in orthopaedic surgery (Roche et al., 2005).

More immediately, many countries have faced substantial backlogs in elective care services as a result of the COVID-19 pandemic. T&O departments were amongst the most severely impacted specialties, due to the cancellation of non-emergency surgical cases, reduction in inpatient stays, reconfiguration of outpatient services, and reassignment of orthopaedic staff to other hospital departments (Graichen, 2020).

The resulting backlog will have a significantly negative impact on the quality of life of the patients waiting, many of which are already experiencing severe and incapacitating conditions (Jenkins, 2020). Already in the UK, this has resulted in nearly twice the number of patients waiting for total hip or knee arthroplasty experiencing a state of life which can be considered as “worse than death” (Clement et al., 2021).

There is also the possibility of a return of unmet demand from the periods of national lockdown and enforced public health measures during the first 2 years of the pandemic. Orthopaedic teams saw large reductions in the number of referrals to departments from 2019 to 2020, with changing patient behaviours and attitudes towards presenting at health services raising concerns that a wave of returning patients may be on the horizon (Hsu et al., 2022).

It is evident then that already pressured orthopaedic departments likely face further system stresses in the immediate and long-term futures. Improving musculoskeletal health not only aids the patient and others within the healthcare system but also yields economic advantages to the employers of sufferers, and the wider economy, through the reduction of working days lost. For the sake of improving patient outcomes and access to care, as well as ensuring the long-term sustainability of health services, it is important that orthopaedic services can be run as smoothly and efficiently where and when possible.

Operational Research and Management Sciences (OR/MS) methods have a rich history of utilisation in healthcare applications stemming back to the 1950s (Royston, 2009), spanning a myriad of problems including healthcare planning; management and logistics; healthcare practice and specialised and preventative healthcare, with the aim of optimising treatment plans and system performance (Rais & Viana, 2011). As a surgical specialty that deals with the diagnosis and treatment of conditions that relate to the body’s musculoskeletal system, many of these problems extend to T&O treatments and settings. These encompass aspects such as resource optimisation for the allocation of hospital beds, operating theatres and equipment, process improvement to identify bottlenecks and patient throughput, and decision-making tools to evaluate the impact of different treatment options. However, the extent to which OR/MS methods have been applied to T&O has been unclear prior to this review.

### 1.2. Contribution

Previous OR/MS literature reviews have looked at applications of such methods to various healthcare departments, sectors, and planning decisions, each highlighting the key trends and breadth of OR/MS applications within their respective area. Reviews that have considered particular planning decisions within healthcare typically look across how this problem has been considered across departments or specialties. For example, Hulshof et al. (2012) produced a taxonomy of OR/MS methods for identifying and classifying planning and control decisions in resource capacity planning and control. Papers were categorised across four decision levels, as well as a spectrum of six services in healthcare delivery. Aspland et al. (2021) present a classification of problems related to clinical pathway modelling, including the scope and extent to which they have been modelled. Further to these, Volland et al. (2017) present an approach to classify literature pertaining to material logistics management within hospitals, separating literature into four mean streams to analyse.

Some reviews have focused on the application of OR/MS methods to specific healthcare disciplines or specialties, such as the Williams et al. (2021) review of OR/MS methods on care planning for frail and elderly patients, which categorised papers according to methodology, hospital setting, and research aims, amongst others. A review of optimisation in mental healthcare and service delivery was conducted by Noorain et al. (2022), considering the methodologies, objectives, constraints and solution approaches to such problems. Erhard et al. (2018) present a classification of mathematical programming approaches to solving physician scheduling problems within hospitals. The application of OR/MS methods in intensive care unit management was reviewed in Bai et al. (2018), classifying literature into several categories, including decision horizons, problem settings, and modelling and solution approaches.

Other reviews have considered problems relating to specific departments and healthcare settings. Cardoen et al. (2010) outline a review of operational research applications to operating room planning and scheduling, finding 247 such applications as of the date of 2009. The application of simulation modelling to Emergency Departments was considered in Salmon et al. (2018), to uncover patterns and trends in such applications. A review of the planning of the collection process in blood supply chains and clinics was carried out by Williams et al. (2020), classifying papers by methodology, application and planning decisions. Additionally, Grieco et al. (2021) present a review of OR/MS methods applied to support decision-making relating to home health care.

To the best of our knowledge, no literature review of OR/MS methods applied to orthopaedic settings and treatments exists, and this work fills this gap. The aim of this research is to help guide healthcare professionals in T&O, as well as OR/MS researchers, through the current literature of OR/MS applications to orthopaedics, including areas of previous/current focus and areas for future consideration. Healthcare professionals can use this work to identify strategies that can help sustain and enhance the accessibility and quality of their services, even during periods of high demand and pressure. OR/MS researchers can use this work to aid their own research with health service partners, as well as identifying the extent of current applications of different methodologies and research gaps for future work.

This paper has been structured as follows. Section 2 introduces our taxonomy for the literature, while Section 3 outlines the processes undertaken in identifying the papers relevant to this research. Section 4 displays the results of using our taxonomy in order to classify the identified papers. Section 5 provides discussion of areas of particular focus and research gaps. Section 6 gives some conclusive remarks of the review as a whole.

## 2. Taxonomy

Originating from biological science, a taxonomy deals with the classification of a population (Hulshof et al., 2012). In the context of a literature review, a taxonomy classifies a population of included papers into a series of categories depending on the focus of the research.

For our taxonomy, we have classified papers according to categories in three main areas: general context (Section 2.1), medical context (Section 2.2), and methodological context (Section 2.3). The categories within these areas have been chosen to present an in-depth analysis of current literature, in order to identify the key research points of focus and research gaps. The overall outline of the taxonomy can also be generalised and adapted to reviews of other surgical specialties, as well as other areas of healthcare.

### 2.1. General context

The classification of papers according to their general context spans areas that are separate to the methodology or research direction of the paper, instead considering their metadata. These were chosen to help answer questions such as who was conducting the analysis, how was the data obtained, and to what degree was the work implemented. The following data was collected on each publication:

- Clarivate Journal Citation Reports category
- Year of publication
- Data source
- Level of implementation
- Continent of application
- Funding status

#### 2.1.1. JCR category

We focused the search on six categories in the 2021 Clarivate Journal Citation Reports (JCR), each chosen so that the search results provide an interdisciplinary view of how OR/MS is not only used within the OR/MS field, but also in other related fields. A synopsis of each JCR category is presented below, which also works to rationalise our decision to include them:

- **Health Care Sciences & Services (HCSS)** – Contains journals that focus on problems arising in healthcare services, disease prevention and health promotion (e.g., Operations Research for Health Care, Value in Health).
- **Health Policy and Services (HPS)** – Captures journals that cover the impact on policy decisions and service improvement within healthcare systems (e.g., Health Care Management Science, Health Systems).
- **Industrial Engineering (IE)** – Captures journals that focus on systems that integrate people, materials and equipment to provide a service (e.g., Computers & Industrial Engineering, Computers & Operations Research).
- **Medical Informatics (MI)** – Contains journals that focus on healthcare information studies (e.g., International Journal of Technology Assessment in Health Care, Journal of Evaluation in Clinical Practice).
- **Operations Research and Management Sciences (OR/MS)** – Covers journals that focus on the application of OR/MS methods to complex problems (e.g., Decision Support Systems, Journal of Simulation).
- **Orthopedics (T&O)** – Contains a subgroup of clinical medicine journals that focus on problems in the orthopaedic specialty (e.g., Clinical Orthopaedics and Related Research, Journal of Arthroplasty).

Two upcoming journals that do not belong to a JCR category were also incorporated, and were appropriately assigned to one of the six categories. These journals were as follows: IISE Transactions on Healthcare Systems Engineering (formally known as IIE Transactions on Healthcare Systems Engineering until 2017) (assigned to IE) and the Proceedings of the Winter Simulation Conference (assigned to OR/MS). It should also be noted that some journals identified in our search may belong to more than one JCR category.

#### 2.1.2. Data source

The approach taken by researchers to collecting data for their models varies considerably (Hox & Boeije, 2005). For this analysis, we have grouped these approaches into three data source categories, where papers can be classified by any one of, or multiple of the following:

- **Primary data** – data that was gathered by the authors themselves, for use in their specified research problem. This can include data collected through observations at a facility, interviews with staff at the service of focus, as well as a survey or clinical trial conducted by authors on a target demographic.
- **Secondary data** – all data that has been used in the model that was initially sourced by a third-party not involved in the research problem. This includes data obtained from historic databases or pre-existing information at a facility, statistical institutions, national registry data, as well as existing evidence or clinical trial results obtained from published journal articles or conference papers.
- **Expert opinion** – data where the authors were unable to obtain primary or secondary sourced data but were able to obtain estimates on the parameters from people considered as experts within the field in study.

#### 2.1.3. Level of implementation

We understand that there are various definitions to analyse the extent to which each model has been implemented in practice. We have chosen to adopt the definitions used in Brailsford et al. (2009) to help keep this review consistent with other OR/MS literature reviews. However, we have adapted the definitions slightly to provide more transparency in how papers were classified in this category. The levels of model implementation used in this analysis are as follows:

- **Theoretical** – A model theoretically proposed by the authors for use in its specified functional area. Includes papers that do not reference the model’s implementation.
- **Conceptualised** – A model that has been discussed with, designed with input from, or obtained primary sourced data in agreement with a client organisation, but one that has not yet been implemented in a working environment.
- **Implemented** – A model that has led to the implementation of its findings in a working environment.

#### 2.1.4. Continent of application

A paper was classified to a continent if it specifically mentions that the analysis was taken from the perspective of a country within that continent, either from a named hospital, healthcare provider, population, or economic analysis. Failing this, the paper is classified as having no continent of application. All the continents identified within our analysis were Africa, Asia, Europe, North America, Oceania, and South America. Doing so allows us to understand the breadth of this type of research around the world.

#### 2.1.5. Funding status

Papers have further been broken down according to any mention of funding for their research, to gain an insight into the extent to which previous research has received financial support. Hence, papers have been categorised by “Funded”, “Not funded” or “No mention”. Research funding can come from a variety of sources, including health services, commercial firms, and research funding bodies. Due to the nuances with some funding sources from country to country, we have opted to just highlight whether funding was mentioned or not, and not the specific source of funding as in Brailsford et al. (2009).

### 2.2. Medical context

Papers have further been broken down by their medical context. These categories were selected to provide coverage of the direction of the paper’s research, as well as the medical settings and backgrounds that the paper is built around. The breakdown of medical context categories is as follows:

- Trauma & Orthopaedics specialty
- Condition area
- Care area
- Secondary and tertiary care area
- Modelling scope
- Modelling perspective
- Research aims.

#### 2.2.1. Trauma & orthopaedics specialty

T&O covers a wide range of musculoskeletal conditions, affecting different areas of the body, with many surgeons specialising in particular areas of the discipline. We have taxonomised papers according to the T&O specialties that they cover, as outlined by the British Orthopaedic Association (British Orthopaedic Association, 2022). The specialties considered in the papers are knee, hip, spine, foot and ankle, fracture (any), general orthopaedics, elbow, sport, hands, paediatrics, and shoulder. General orthopaedics contains papers that model settings with no specific speciaity, for example work that models a T&O outpatient department. Papers can be classified by more than one of these specialties.

#### 2.2.2. Condition area

Within other healthcare literature reviews, a typical classification is the condition area of the patient, these being acute, chronic, and surgical (Aspland et al., 2021; Williams et al., 2021; Zhang et al., 2014). This is transferable to orthopaedics too, with patients suffering from a wide range of conditions caused in many ways. Adopting this classification here may provide insight into how orthopaedics compares to the areas of healthcare in other literature reviews. It should be noted that papers can cover more than one condition area.

- **Acute** – These conditions are unforeseen and often severe in nature, for example a fractured bone caused in sudden circumstances such as a fall, or a surgical complication such as venous thrombosis (VTE).
- **Chronic** – A long-standing or developing condition, for example osteoarthritis, or a prolonged fracture event or surgical complication.
- **Surgical** – An acute surgical procedure for an orthopaedic condition, for example total knee arthroplasty, or spinal fusion.

#### 2.2.3. Care area

Generally, health services are divided into several separate care areas, these typically being Primary care, Secondary care, Tertiary care and Community care, and are intended to work as a single integrated care system with patients referred to the care area that best suits their treatment needs (For example, NHS Digital (2022)). Within an orthopaedic setting, we could consider the care areas as follows:

- **Primary** – Typically a patient’s first point of contact in the care system, for example a General Practitioner visit.
- **Secondary** – Elective or emergency care, usually within a hospital, for example an outpatient appointment, inpatient stay, surgery, or in-hospital physical rehabilitation.
- **Tertiary** – Highly specialist treatment, for example spinal surgery.
- **Community** – Services within community settings, for example long-term care facilities such as nursing/residential homes or home care.
- **Patient Progression** – Outside of these areas, there are some papers that model events of orthopaedic conditions that do not take place within the integrated care system. These include medications taken as part of a longer-term condition or recovery, as well as how the patient’s condition or recovery progresses over a period of time outside the care system, for example the progression of osteoarthritis or implant condition over time, or the development of a pulmonary embolism or venous thrombosis event post-discharge.

Some papers will contain more than one care area, showcasing the transitions from one area to another. Considering the care areas studied within the identified literature reveal which areas have received the most attention in T&O research, and the challenges these areas encounter.

#### 2.2.4. Secondary and tertiary care pathway area

Papers in the secondary and tertiary care areas were further classified according to the areas within the pathway that they modelled, in order to gain a better understanding of the extent to which these pathways are modelled. This has been done using the following categories, where papers can contain multiple categories:

- **Patient Assessment** – Where the patient is initially triaged, or assessed within a secondary/tertiary care, for example, an Emergency Department triage assessment.
- **Outpatients** – Events that occur within an outpatient setting, prior to the patient receiving surgery, for example, an outpatient consultation appointment, a magnetic resonance imaging (MRI) scan, or a computed tomography (CT) scan.
- **Inpatients** – An inpatient stay in hospital, that is not part of a post-surgical acute inpatient recovery, for example, inpatient hospitalisation following an orthopaedic injury.
- **Surgery** – Any event pertaining to the perioperative period, that is not post-surgery recovery, for example, the surgical event itself, the anaesthetic process, admission for surgery.
- **Post-Surgery** – The post-surgical recovery stay.
- **Rehabilitation** – Any form of physical rehabilitation carried out as a treatment, or part of long-term recovery, for example, rehabilitation after surgery, physical therapy, physiotherapy.
- **Follow-up** – The surgical follow-up appointments to assess the patient’s recovery.

#### 2.2.5. Modelling scope

The scope of the model indicates the extent to which the model has captured the orthopaedic setting. It considers whether the modelled activities focus on the patient themselves, or rather, the system around the patient, similar to what has been done in Aspland et al. (2021). Papers have been classified by clinical, department, or hospital scopes, as outlined below:

- **Clinical** – Papers that focus on modelling a series of clinical events that revolve around the patient, or rather, the patient’s progression both in and out of care settings.
- **Department** – The focus falls on modelling a single department within an orthopaedic setting, for example, an outpatient T&O department.
- **Hospital** – The interactions between an orthopaedics department and other departments within a hospital have been modelled as part of the patient’s treatment. For example, this could involve a patient visiting the radiology department from the orthopaedic department for diagnostics.

#### 2.2.6. Modelling perspective

The modelling perspective is useful to help understand which viewpoints the research is being conducted from, these being the patient, the provider, the societal perspective, or any combination of the three. Doing so can allow for a deeper understanding of other categories within this taxonomy, for example, the perspective the outcome measure is taken from. For papers considering cost analysis of some form, direct costs incurred to both the patient and provider consist of costs of any treatment received from a healthcare service, including ambulatory care, emergency care, and medications. Indirect costs make up the societal costs sustained as a result of the research area in question, and include loss of productivity, absenteeism and premature mortality (Kamaraj et al., 2021).

- **Patient** – The research is modelled from the patient’s perspective. This includes models of a patient’s health progression, cost analyses containing direct costs incurred to the patient, and patient-lead decision-making.
- **Provider** – The research is modelled from the provider’s perspective. This includes models of the healthcare service and departments, staffing levels and configurations as well as direct costs incurred to the healthcare provider or healthcare insurance provider.
- **Societal** – The research is modelled from a wider societal perspective. This includes population studies, as well as indirect costs incurred as part of services and treatments.

#### 2.2.7. Research aims

Here, papers have been classified assigned to three main research aims, these being examining, forecasting, and improving, as in Williams et al. (2021), where papers can also be classified as any combination of the three. These three aims outline the direction of the research, and what the model strives to achieve within its functional area. By adopting this classification from Williams et al. (2021), we can also compare how orthopaedics compares to the findings of their review.

- **Evaluating** – Using OR/MS methods to assess how the modelled area performs in its current configuration.
- **Forecasting** – Using OR/MS methods as a means to predict future scenarios of the current configuration.
- **Improving** – Using OR/MS methods to suggest or implement improvements to the current configuration of the modelled area.

### 2.3. Methodological context

In this subsection, we outline how we have taxonomised papers according to their methodological context. The methodological context refers to the OR/MS methods applied to the medical context of each paper, as well as the outcomes they return. This area has been split into the following categories:

- Primary OR/MS methodology
- Secondary OR/MS methodology
- Outcome
- Functional area
- Planning decisions

#### 2.3.1. Primary OR/MS methodology

A broad range of OR/MS methods have been applied to orthopaedic settings, highlighting their usefulness in improving areas of the discipline. Considering the methods applied to these settings provides a deeper technical understanding of the research conducted in this field. It offers insights into the specific methods applied to address various problems, while also highlighting the relative prevalence or scarcity of certain methods. The methods used can be clustered into ten OR/MS areas, with each area and its contained methods given as follows:

- **Decision Analysis** – Includes decision trees and break-even analysis.
- **Graph Theory** – Includes, for example, social network analysis.
- **Heuristics** – Includes neighbourhood search, as well as metaheuristics (Genetic algorithm, Particle swarm optimisation, Scatter search, Tabu search).
- **Markov** – Includes Markov chain models.
- **Multi-Criteria Decision-Making (MCDM)** – Includes, for example, analytical hierarchy processes.
- **Optimisation** – Includes linear programming methods, including integer, mixed-integer, goal, and the knapsack model.
- **Queueing Theory** – Includes the application of queueing theory.
- **Simulation** – Includes agent-based models (ABM), discrete-event simulation (DES), Monte Carlo, microsimulation, and system dynamics.
- **Soft OR** – Includes, for example, the Delphi method.
- **Statistical Analysis** – Includes statistical methods that were paired with a secondary OR/MS method.

Some papers may include multiple primary methods, in which case they have been classified into more than one of these categories.

#### 2.3.2. Secondary OR/MS methodology

The application of OR/MS methods as secondary, or subsidiary, methods was also analysed. The secondary methods were classified according to the same criteria as the primary methods in Section 2.3.1, and then further broken down into the exact methods used. The methods identified include the Delphi method, simulation, and sensitivity analyses. Some papers can include multiple of these.

#### 2.3.3. Outcome

The outcome, or outcomes, of interest are what drives the direction of the research, and within this literature, can be clustered into any one, or combination, of the following three categories:

- **Cost** – Any papers where one of the outcomes directly relate to financial cost, for example, cost analysis, cost-effectiveness analysis, cost-utility analysis.
- **Health** – Any papers that consider factors relating to the patient’s health, or treatment effectiveness or options, for example, Quality Adjusted Life Years (QALYs), medical decision-making.
- **Time** – Any papers that pursue an outcome related to time, for example, patient scheduling, waiting times, staff utilisation.

Analysing the outcome measures of papers offers a look into what the key performance indicators are for different areas of orthopaedic care, and what OR/MS methods have been applied to measure them.

#### 2.3.4. Functional area

The functional area dictates the type of analysis conducted within the research, with respect to the outcome measures: cost, health, time. Here, there are 15 different functional areas studied, which are listed below:

- **Bed Management**
- **Capacity Planning**
- **Cost Analysis**
- **Cost-Effectiveness Analysis**
- **Cost-Utility Analysis**
- **Expected-Value Decision Analysis**
- **Health-Utility Analysis**
- **Location Planning**
- **Manufacturing**
- **Medical Decisions**
- **Medical Simulation**
- **Patient Scheduling**
- **Risk-Benefit Analysis**
- **Staff Utilisation**
- **System Design and Planning**

#### 2.3.5. Planning decisions

Hulshof et al. (2012) outline a taxonomic classification of the planning and control decisions made in resource capacity planning within healthcare. Their taxonomy presents three different levels of planning decisions, these being Strategic, Tactical, and Operational, or any combination of these. Assessing these gives an insight into the planning horizons being considered for research relating to resource capacity planning. A summary of each planning decision is given below:

- **Strategic** – Addresses structured decision-making in the design, dimensioning and development of the delivery process, typically over a long-term planning horizon, for example location planning and dimensioning resource capacities.
- **Tactical** – Addresses the organisation of the operations of the delivery process, typically over a mid-term planning horizon, for example staff-shift scheduling.
- **Operational** – Addresses the decision-making relating to the execution of the delivery process designed at the individual patient and individual resource levels.
  - **Offline** – The advance planning of operations, regarding elective demand, for example patient-to-appointment scheduling, surgical case scheduling.
  - **Online** – Mechanisms that monitor the process and react to unplanned or uncertain events, for example dynamic rescheduling of elective patients due to emergency demand.

## 3. Literature search methodology

In this section, we outline the methodology used in searching for relevant literature to this study, followed by the process used in deciding whether a publication should be included in the analysis or not.

### 3.1. Data sources and paper identification

To identify the breadth of the current literature of operational research methods applied to orthopaedics, a structured search was performed using the Scopus electronic database. The search string features a range of OR methods, as well as orthopaedic terms, to provide a comprehensive overview of the literature. Search results will then contain relevant journal articles and conference proceedings, which feature at least one OR method, and at least one orthopaedic term in either their title, abstract, or list of keywords. No restrictions were placed on the publication date of the results, though the search has been limited to publications in the English language.

Our search string included a wide selection of OR methods and orthopaedic terms, with the OR/MS methods and orthopaedic terms shown in supplementary Appendix A, and the corresponding search string presented in full in supplementary Appendix B. In order to obtain a complete search, the articles and papers included in the results had to include at least one term from the “OR Method” group, and one term from the “Orthopaedic Term” group, within their title, abstract or keywords. The Boolean operators “AND” and “OR” were used to concatenate the terms from these groups. For each group, the terms within were linked using the “OR” command, to ensure at least one term was included from there, while the groups themselves were connected with the “AND” command to ensure at least one term was taken from both groupings.

The asterisk character has been used at the end of certain terms to allow for simplification in searching for words with various endings. For example, “Agent based model*” will return results for “Agent based model”, “Agent based models” and “Agent based modelling”. Similarly, the question mark character has been incorporated into the term “Optimi?ation” to allow for variations in letters within the term, hence allowing for the search of both “Optimisation” and “Optimization”. The dollar character is used at the end of terms that have multiple possible endings of up to one extra character, for example “Heuristic$” will return results for both “Heuristic” and “Heuristics”.

To ensure a wide cross-section of OR methods, a modified version of Williams et al. (2021) search string is used, which in itself is an amalgamation of terms identified within the aforementioned Hulshof et al. (2012) review, and the Palmer et al. (2017) review of OR methods for modelling patient flow and outcomes. Our search string expands on this to include a wider range of OR techniques. While the string in Williams et al. (2021) includes machine learning approaches such as “Clustering” and “Neural Network$”, we have chosen to remove these from our string. An initial search on Scopus which included these terms, as well as further machine learning approaches such as “Regression” and “Support Vector Machine”, resulted in a substantial increase in the number of results over the search without these terms. Hence, it is suggestive that a separate literature review into machine learning applications in orthopaedics could be worthwhile. The terms “Computer Simulation” and “Simulation” were also dropped from our string, as these terms were also found to be profoundly increasing the number of results by including 3-D orthopaedic models of bones and joints, computed tomography (CT) imaging and virtual reality surgical training, amongst others. To ensure that appropriate simulation modelling has still been captured well by our search string, we have included the terms “Agent based model*”, “Agent based simulation”, “Discrete event simulation”, “Markov chain”, “Markov decision”, “Markov model”, “Monte Carlo simulation” and “System$ dynamics”.

The orthopaedic terms included in the search string have been derived from the Medical Subject Headings (MeSH), a controlled vocabulary thesaurus of medical terms for the indexing and cataloguing of literature by the National Library of Medicine (National Library of Medicine, 2022). We have included terms listed under the “Orthopedic Procedures” headings (under both the “Therapeutics” and “Surgical Procedures, Operative” headings in the “Analytical, Diagnostic and Therapeutic Techniques and Equipment” category), as well as terms under the “Orthopedic Equipment” heading (under the “Surgical Equipment” heading in the “Analytical, Diagnostic and Therapeutic Techniques and Equipment” category). For simplicity, terms derived from the MeSH that contained overlapping terminology have been shortened to just that mutual phrase, where appropriate. For example, in our string “ligament reconstruction” will return results associated with both “Anterior Cruciate Ligament Reconstruction” and “Posterior Cruciate Ligament Reconstruction” MeSH terms.

We focused the search on six categories in the 2021 Clarivate Journal Citation Reports (JCR), each chosen so that the search results provide an overview of OR/MS methods applied to orthopaedic settings, and to filter out as many irrelevant papers as possible. To mitigate the potential limitations of narrowing the search to these six categories, we conducted both a forward and backward search on our initial set of relevant papers to identify any papers that may not have been captured.

### 3.2. Study selection and data extraction

An initial set of 1,936 papers was identified in our original search of the Scopus database, these papers being identified through the search string detailed previously.

All of these 1,936 papers underwent a screening process to identify papers for full-text analysis. A publication met the inclusion criteria for full-text analysis if it was identified, or implied, that the publication described an OR technique being applied to an orthopaedic problem; otherwise, the paper was excluded. A total of 320 papers passed this stage of the screening process. The full text of each of these papers was then read in order to taxonomise the paper or to exclude them from the full analysis. Publications were excluded at this stage if it became apparent that they did not contain an OR method applied to orthopaedics.

On completion of the screening process for the original search, a total of 288 publications were identified and included in our full analysis. This equates to approximately 14.88% of the initial 1,936 papers from the original Scopus search being included in the full analysis. The total of 288 does not include 8 identified literature reviews, which were not included in the full analysis but were included in conducting the backward and forward searches. These literature reviews were of health economic approaches to evaluate orthopaedic treatments. From an OR/MS perspective, these review papers include Markov models and decision analysis models applied to a narrow scope of just one specific treatment option or area of orthopaedics.

The next step was conducting a forward search of these 296 publications (including the review papers), which seeks to identify all publications that have cited these 296 papers within their text (Webster & Watson, 2002). The forward search identified 6,921 papers, of which 1,092 were recognised as duplicates from the original search. Through title, abstract and keyword screening, 168 papers were left from the forward search for full-text analysis. Following the completion of the full-text analysis, a final total of 148 papers from the forward search were included within the final analysis, which equates to approximately 2.14% of papers from the initial 6,921 identified.

The final step was conducting a backward search of the 296 papers identified from the original search, using Scopus to collate all publications referenced within these 296 papers (Webster & Watson, 2002). The backward search identified 10,435, of which 814 were recognised as duplicates from either the original search, or the forward search. Following title, abstract and keyword screening, 58 papers remained from the backward search for full-text analysis. These papers were then subject to the reading of their full text, in order to classify the papers according to the taxonomy, or to exclude them from the full analysis. Following the completion of the full-text analysis, a final total of 56 papers from the backward search were included within the final analysis, which equates to approximately 0.54% of papers from the initial 10,435 identified.

**Figure 1.**
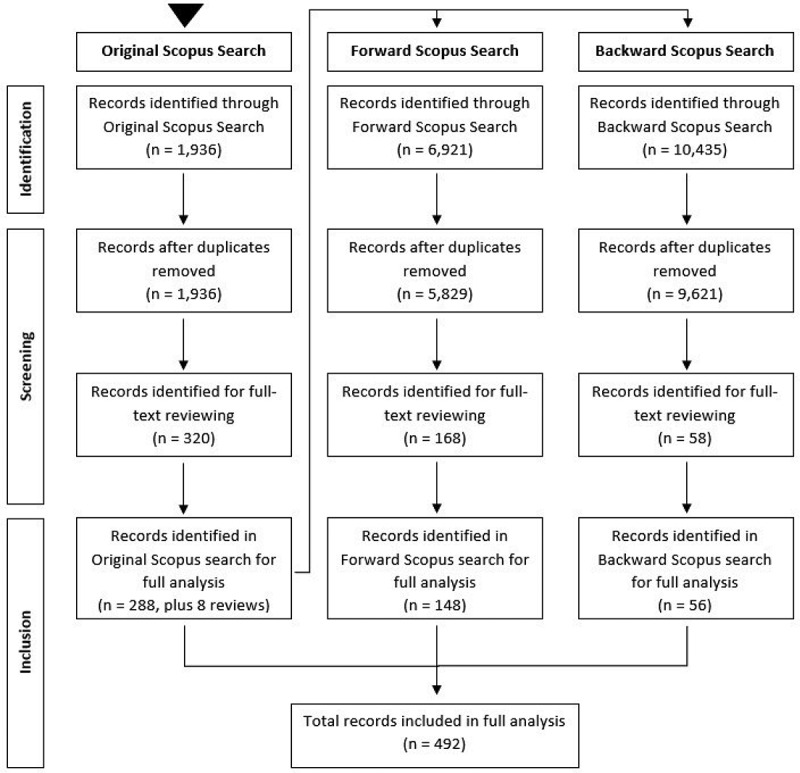
Flow diagram of the literature review search process.

This leaves a final total of 492 papers included within the final analysis, spanning the original, forward, and backward searches, as well as the separately identified paper. The search process, detailing the identification, screening and inclusion of literature for our analysis is presented visually in Figure 1. Of these 492, 463 are listed as journal articles, while the remaining 29 are conference papers. A complete list of these 492 papers, as well as the 8 existing review papers on health economic approaches, can be found in supplementary Appendix C.

For all of the 492 papers included in our final set for full analysis, we taxonomised them according to the criteria outlined in the previous Section 2 within a Microsoft Excel spreadsheet, and performed data analysis using Python. The analysis of the identified literature is presented in Section 4, whilst a full breakdown of every paper identified, and how they were classified can be found within the CSV file archived on Zenodo at https://doi.org/10.5281/zenodo.7995520 (Howells et al., 2023). This can be read and analysed using the corresponding Jupyter Notebook file, which allows for all figures in Section 4 to be reproduced. Furthermore, by providing access to these files online, it allows readers to conduct their own analyses on the taxonomy, and to view results which we have not reported on in Section 4.

## 4. Analysis of current literature

This section reports on the key findings obtained from the classification of the included papers according to our taxonomy. We have structured this section so that the reporting of our results helps answer four research questions:

- Who has done the modelling? (Section 4.1)
- What has been modelled? (Section 4.2)
- Why has it been modelled? (Section 4.3)
- How has it been modelled? (Section 4.4)

### 4.1. Who has done the modelling?

**Figure 2.**
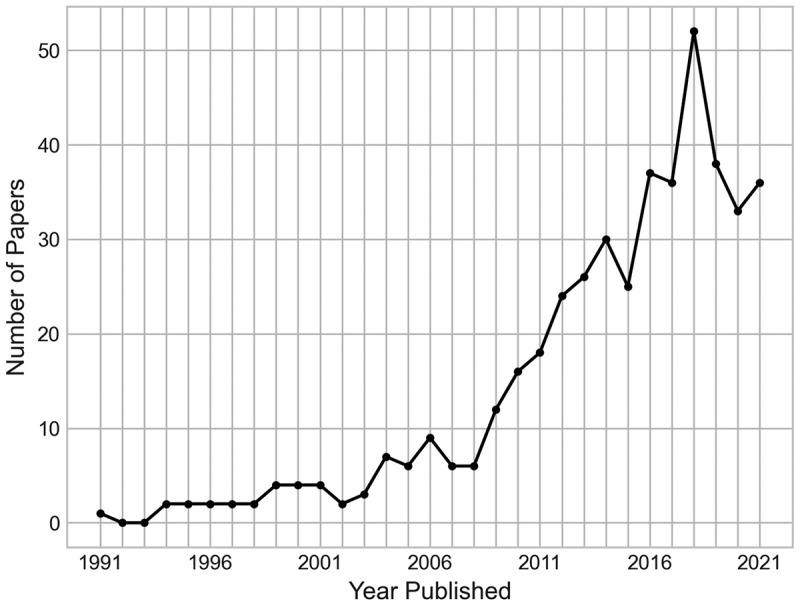
Number of papers by their year of publication (up to 2021).

The papers identified in this analysis range from publication between 1991 and 2022, with a breakdown of the frequency of papers published by year given in Figure 2. Within our search, the earliest found publication of an OR/MS method applied to an orthopaedic treatment came in Jacobson et al. (1991), which utilised a decision tree to perform a cost-effectiveness analysis on options for preventing late prosthetic joint infections in prosthetic limb patients undergoing dental surgery. Their decision tree further included the possible amputation of limbs.

The results show a generally increasing trend in the number of operational research applications to orthopaedics, with 89.01% of the papers being published from 2009 onward. Not shown in Figure 2 are the 42 papers published in the year 2022, as this analysis was conducted before the end of the year, so the results for this year would be incomplete. However, these 42 papers from 2022 are included within all other analyses in this section.

Following 2018, the number of publications per year has not followed the increasing trend prior to this. We hypothesise two possible reasons for this. One such explanation of this drop would be the impact of the COVID-19 pandemic on research. Social distancing and other public health measures had a profound impact on clinical trials, with most non-COVID related research suspended (L. Harper et al., 2020). Data collection has been reported as not being possible to carry out as planned during the pandemic, with many researchers finding difficulty in enrolling and recruiting patients to clinical trials, as well as the challenges imposed by tele-health (Sathian et al., 2020; Waterhouse et al., 2020). The financial impact of the pandemic has affected research funding too, with less research relying on public funding (Webster, 2020).

Another possible reason for the recent dip in published research could be associated with a publication or identification lag. Since this dip occurs in 3 years prior to this research, it could be that some published work was not yet identified by the search engine for the initial search, or was yet to be cited in or by another papers so did not appear in the backward or forward searches. It is unclear whether or not this is a theme in other literature reviews, as many categorise the years of publication by groups of years, rather than year-by-year.

**Figure 3.**
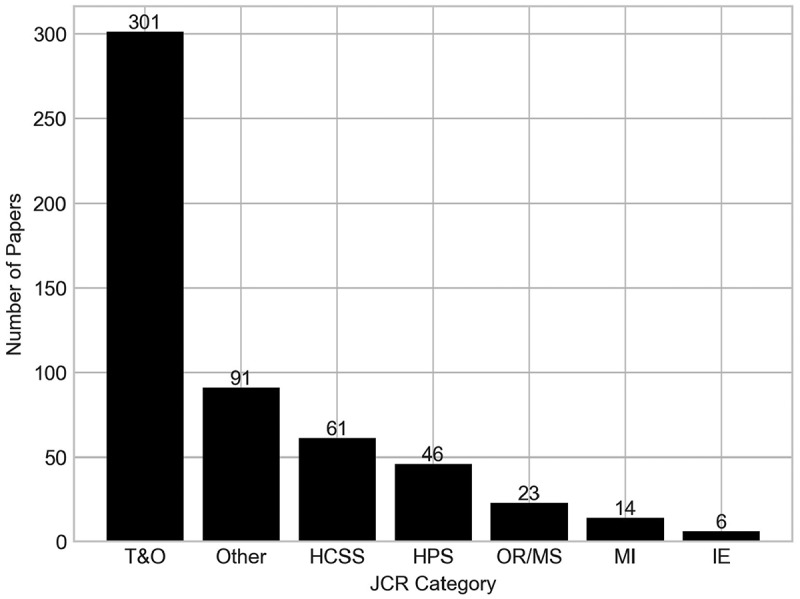
Number of papers by their JCR category. Papers with an ISSN not within any of the six JCR categories have been classified within ‘other’.

**Figure 4.**
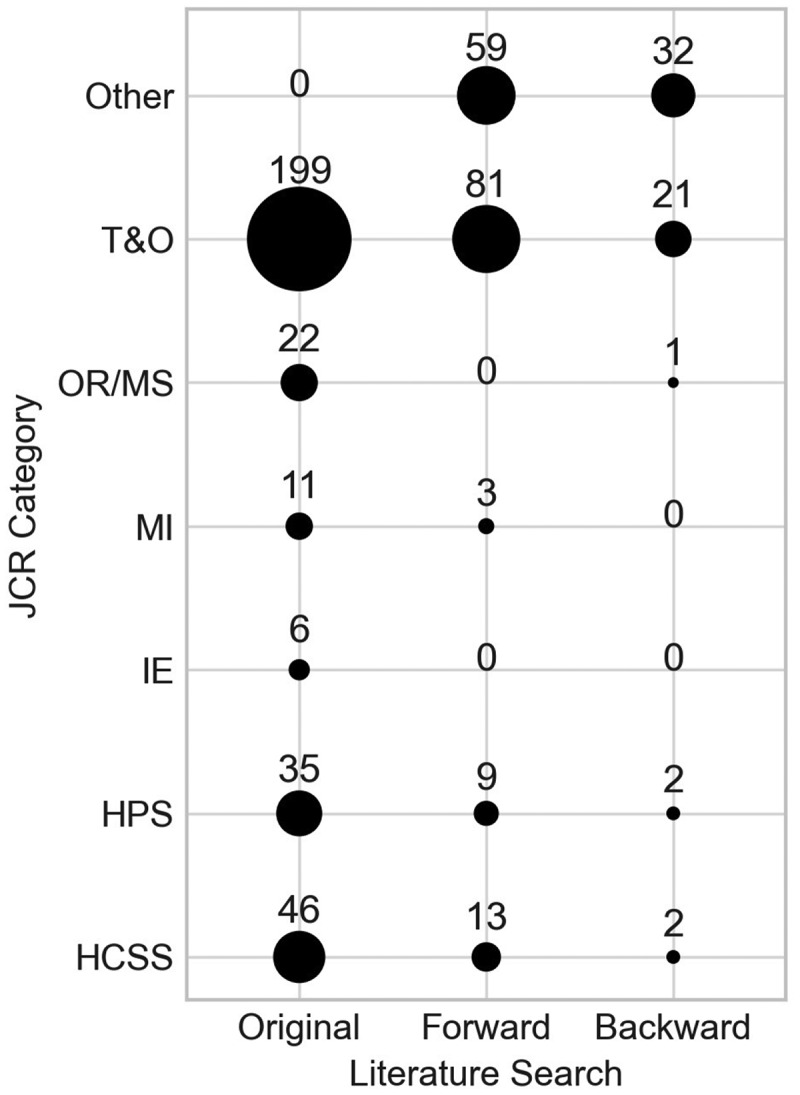
Number of papers by their JCR category and the Scopus search they were identified.

Figure 3 presents the quantity of papers assigned to each JCR category included in the search string, whilst Figure 4 shows a cross-analysis which categorises papers according to their JCR category and the Scopus search in which they were originally found.

Some papers included in the analysis have an ISSN that do not relate to any of the six categories, of which 59 are from the forward search, and 32 are from the backward search. These papers have been classified here as “Other”.

The figure reveals that the vast majority of papers fall within the T&O category, with 240 more than the next highest category from the search string (HCSS). IE and MI are the categories with the lowest number of papers, suggesting that orthopaedics is not being used as a ground for innovative OR/MS methods, rather than just applications. It is still worthwhile to include these categories though, so that we can view their perspective on T&O modelling.

A more detailed analysis of the numbers reveals that 48 papers belong to more than one of the six JCR categories, which explains the total of papers from Figure 3 amounting to more than the 492 papers in the analysis.

Across all three searches, 401 of the 492 papers belong to one of the six JCR categories, and of those 401, 71.82% were identified in the original Scopus search, highlighting the robustness of the search string. As seen by Figure 4, most papers not found in the original string that do belong to a JCR category belong to the T&O category, suggesting that there is some terminology used in these papers that was not identified by the search string.

Of the 91 papers that were published in a journal that had to be classified as “Other”, some journals with notable contributions to this review include Osteoporosis International (8 publications), JBJS Open Access (4 publications), and PLoS ONE (4 publications).

**Figure 5.**
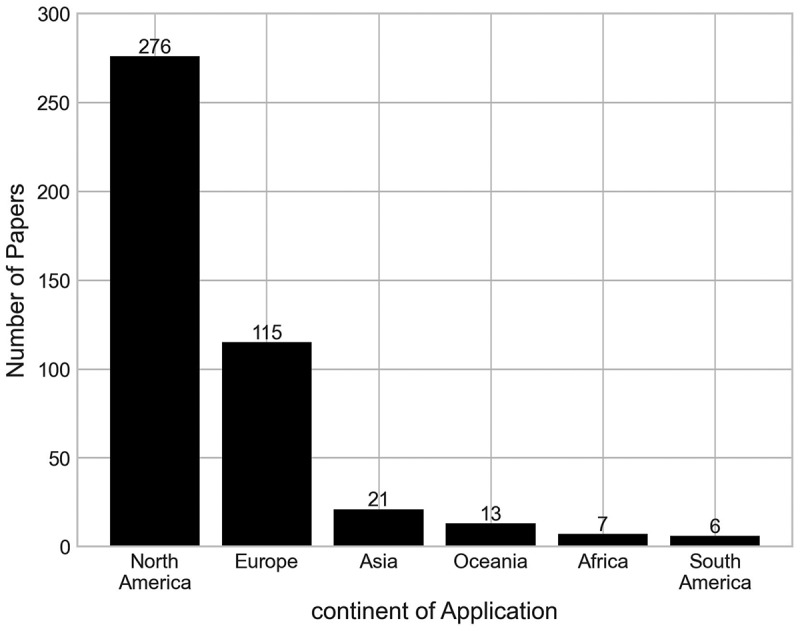
Number of papers by their continent of application.

It can be seen in Figure 5 that 56.10% of the papers are from applications within North America, followed by 23.37% of papers from Europe. The continents with the lowest number of applications were South America and Africa with five and six applications, respectively. The burden of musculoskeletal disorders has differed greatly between different countries over recent decades. The Global Burden of Disease (GBD) 2019 report found that there is a lower burden of these disorders in countries with higher sustainable development index (SDI) scores than in those with lower scores (Liu et al., 2022). This perhaps could be an explanation for the increased number of publications in North America and Europe, regions of lower SDI scores, compared to other continents as the increased burden of these conditions here results in increased demand for orthopaedic services (Sustainable Development Index, 2022).

We found 54 papers had no continent of application, this is either because the area that the work was applied to was left ambiguous within the text, or the work could be applied across many or all of the continents, such as when considering the effect a treatment has on a patient’s quality of life.

**Figure 6.**
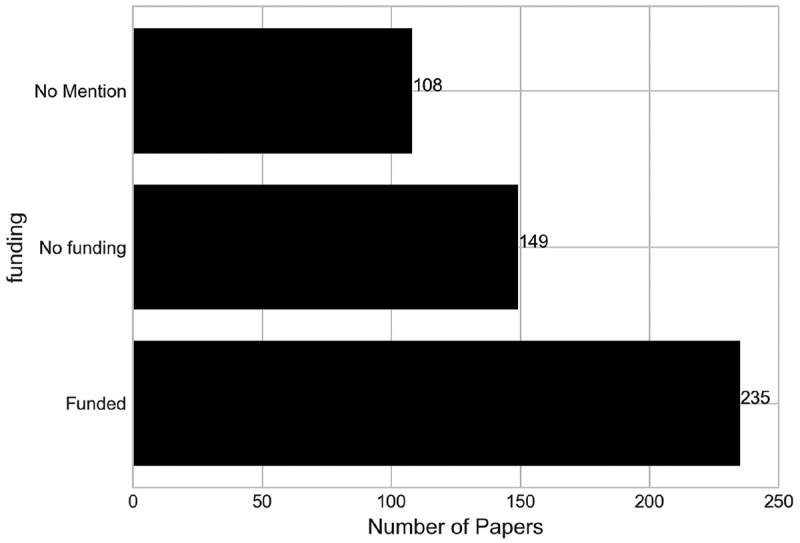
Number of papers by their funding source.

We found that less than half of all papers (47.76%) actually reported having received funding for their research, as evidenced in Figure 6. Additionally, 149 papers (30.28%) reported not receiving any funding for their work, whilst 108 papers (21.95%) made no reference to receiving any funding at all. This highlights quite a large percentage of papers in this literature cohort that do not disclose research funding in their publications.

In terms of whether papers were funded or not, our results align similarly with the findings of Brailsford et al. (2009), who found in their review of simulation and modelling in healthcare that 60% of papers in their analysis reported no formal funding.

With this subsection providing insight into who has done the modelling in T&O, it is worth uncovering what the specifics of these models entailed, which will be explored in the next sub-section.

### 4.2. What has been modelled?

**Figure 7.**
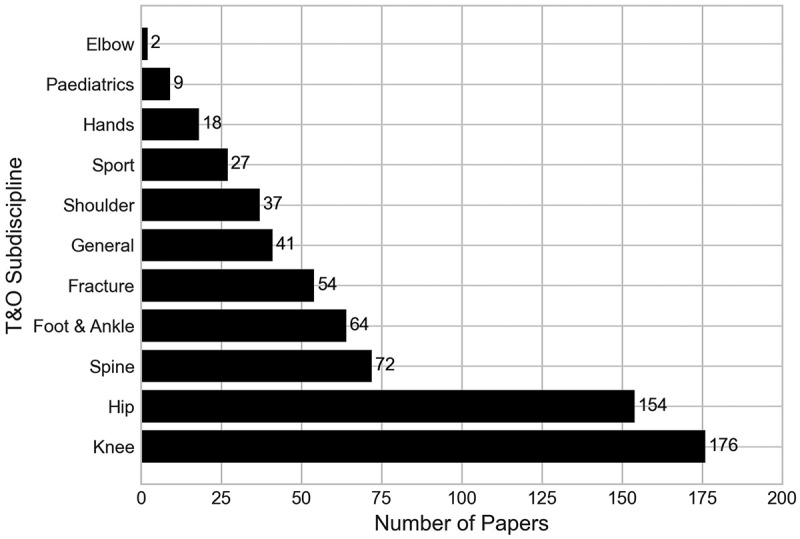
Number of papers by the Trauma and orthopaedics specialties they consider.

Within the T&O specialty category, we found that the most frequently occurring specialty were knee-related conditions or surgeries, amassing 176 publications (35.77%). This is followed closely by hip, which appears in 154 papers (31.30%). This perhaps could be expected, as surgeries relating to knee and hip conditions are among the most common, and expensive orthopaedic procedures (Blom et al., 2021; Weiss et al., 2014; Zhang et al., 2010). The least common sub-specialties found were elbow (2 papers), paediatric orthopaedics (9 papers) and hands (18 papers). One explanation of this could be that in contrast to knee and hip conditions and procedures, elbow and hand conditions may be less common, or much less expensive procedures, so there is less need to optimise these procedures. A full breakdown of the number of papers containing each sub-specialty in the taxonomy is given in Figure 7. Generally, most papers were found to model just one of the T&O specialties within our taxonomy, with 355 papers doing so. However, this number does include all papers classified as “general”, which in itself would consider a myriad of these specialties. Furthermore, 119 papers considered two specialties, 11 considered three, and 7 considered four.

We found that on the whole, papers that included a specific sub-specialty overwhelmingly favour cost or health-related outcomes, most commonly as part of cost-effectiveness analyses in an effort to mitigate the costs of these expensive procedures. This is the case in Gottlob et al. (1999), which conducts a cost-effectiveness analysis of treatment options for anterior cruciate ligament reconstruction (knee) in young adults. Papers that were classified as general had a greater proportion of time-related outcomes though. These papers were classified as general as they typically model an orthopaedic department (Rohleder et al., 2011), or operating theatre (Saadouli et al., 2015), with the focus being on improving the patient flow through these systems, or scheduling system.

**Figure 8.**
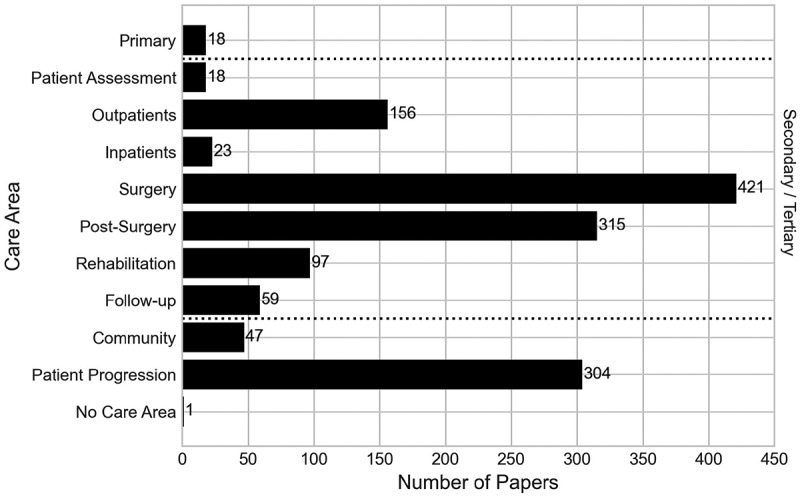
Number of papers by their care area within the care system.

The results of the care areas considered within the papers are presented in Figure 8. One paper has been classified as having no care area, as it presents a manufacturing supply chain problem of customised orthopaedic implants to be used in orthopaedic surgery (Hauser et al., 2021). This paper has been included in the analysis as it presents the only paper of its nature in the search results.

The vast majority of papers in this analysis have considered some level of secondary care in their research, with 445 papers (90.45%) doing so. Also, under a high-level consideration is the patient progression category, which features 304 papers (61.79%). The lowest modelled care within the integrated care pathway area is primary care, featuring in just 18 papers (3.66%).

We found that 327 papers (66.46%) feature more than one care area, modelling transitions between multiple care areas included in this analysis. The most commonly occurring combination of care areas was secondary care with patient progression, appearing in 273 papers. This is the case in Peersman et al. (2014), which uses a Markov model to compare the cost-effectiveness of knee surgery. Within secondary care, it considers outpatient events, alongside the perioperative process and rehabilitation, whilst also considering the long-term effects of the treatments outside the integrated care areas overly yearly intervals. Aside from patient progression, the most frequent intersections of care areas within the integrated care system were secondary care and community care (43 publications), secondary care and tertiary care (31 publications), and primary and secondary care (16 publications).

Within secondary and tertiary care, the most modelled part of these pathways that is modelled is the surgery, which has been modelled in 421 papers (85.57%), as evidenced in Figure 8. The next most frequently modelled part is the post-surgery period, appearing in 315 papers (64.02%). These two parts of the pathway are frequently interlinked, being combined in 305 of the papers in total, highlighting the holistic perioperative procedure as being well modelled in the literature. This is the case in Saadouli et al. (2015), where they worked with an orthopaedic surgery department in Tunisia, using optimisation and simulation approaches to improve the scheduling of operating rooms and recovery beds. The least-modelled parts of these pathways are the patient assessment, and inpatient stays, with 18 and 22 papers, respectively.

**Figure 9.**
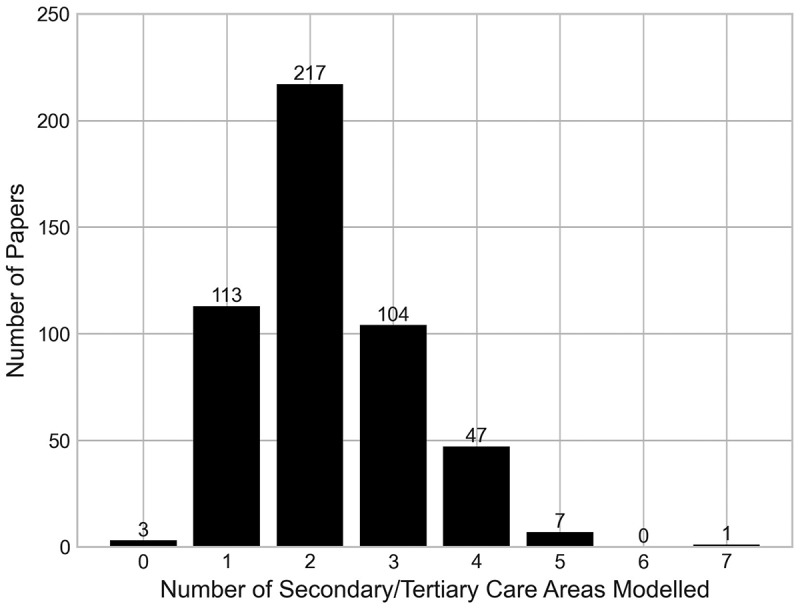
Number of papers by the number of parts of the secondary/tertiary pathway that they have modelled.

Figure 9 shows the breakdown of the number of secondary/tertiary care areas modelled in each paper. Three papers have been classified as modelling no areas of the secondary/tertiary. These include the aforementioned paper by Hauser et al. (2021) on the manufacturing supply chain, as well as one paper by Otto et al. (2016) that considers only primary care, and one by Abdel et al. (2020) that considers community care and patient progression.

Papers most commonly model two parts of the pathway, followed by one and then three. Generally, most areas of the pathway are not modelled within a single paper, showing that the orthopaedic secondary/tertiary care pathway is generally not modelled in a holistic sense. This could be explained by healthcare teams not previously having a need, or interest to model such systems, or it could also be possibly due to historical methodological limitations making it difficult to do so.

The scope of these papers differed greatly, however, with 456 papers (92.68%) having been identified as having a “Clinical” scope, as is the case in Barlow et al. (2017), which presents a Markov model outlining transitions between clinical events both in and out of hospital care. Aside from the “Clinical” papers, just 29 papers (5.89%) are “Hospital”, and 7 papers (1.42%) are “Department”. This demonstrates that there limited instances of hospital and department modelling scopes in the literature compared to clinical scopes.

**Figure 10.**
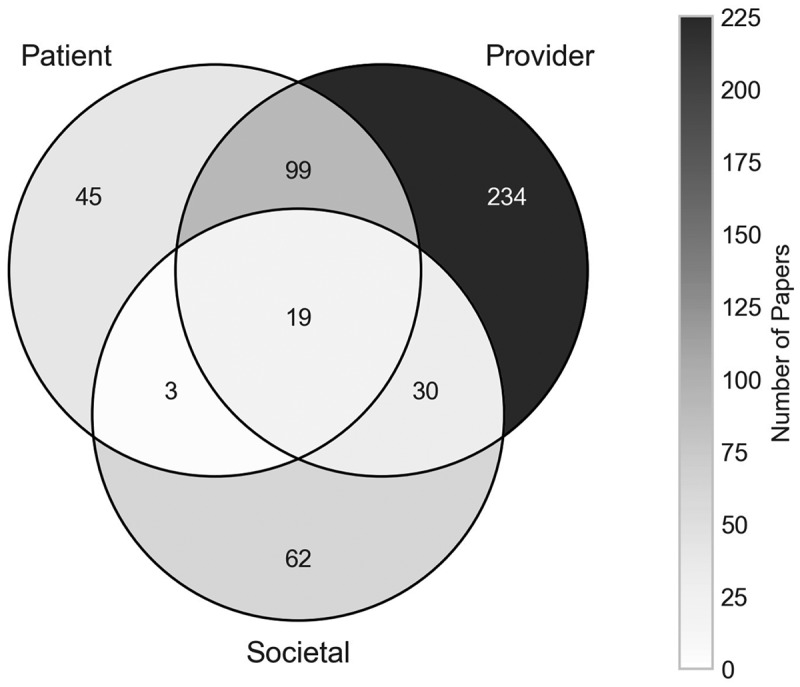
Number of papers by their modelling perspective.

Although most papers have a clinical scope, their overall perspectives and planning horizons might differ. Considering the modelling perspective, we see from Figure 10 that the provider perspective is most frequently occurring across the papers, with it being used in 382 papers (77.64%). It is frequently paired with the patient perspective too, as in 99 papers considering both and 19 papers considering all perspectives, mostly for financial analyses that consider costs incurred to both the patient and the healthcare provider. Papers that left the perspective of the cost analysis ambiguous, or from the payer’s perspective have been classified as both patient and provider, as the costs could be assumed to be incurred to either one.

In general, there is a large quantity of papers covering each of the three perspectives, making this a fairly well-represented area of the literature.

**Figure 11.**
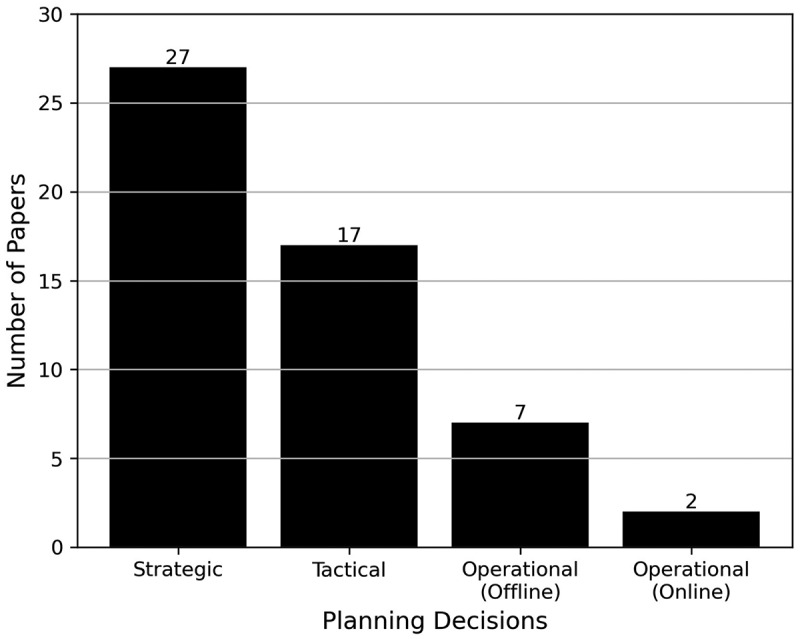
Number of papers by their planning decision level.

Figure 11 illustrates the distribution of the planning decision levels among the 43 papers that addressed resource capacity planning within this analysis. Of these 43, eight considered more than one level, with five containing both strategic and tactical levels, one containing tactical and operational (offline) levels, one containing strategic, operational (offline) and operational (online) levels, and one containing strategic, tactical, and operational (offline) levels.

Aspland et al. (2021) highlighted that the planning levels extend beyond the OR/MS JCR group that Hulshof et al. (2012) focused on within their review. Our results support this claim, with evidence of planning levels being found across all six of the JCR categories used within this research, as shown in Table 1.

**Table 1.** Number of papers by their JCR category and planning decision level.

	Strategic	Tactical	Operational (Offline)	Operational (Online)
HCSS	7	3	0	0
HPS	11	5	1	0
IE	1	2	3	0
MI	2	1	0	0
OR/MS	11	8	5	2
T&O	1	1	1	0

The upcoming sub-section will explore why the subject of the research has been modelled, to answer some of the key motivations that have driven the research in this field thus far.

### 4.3. Why has it been modelled?

**Figure 12.**
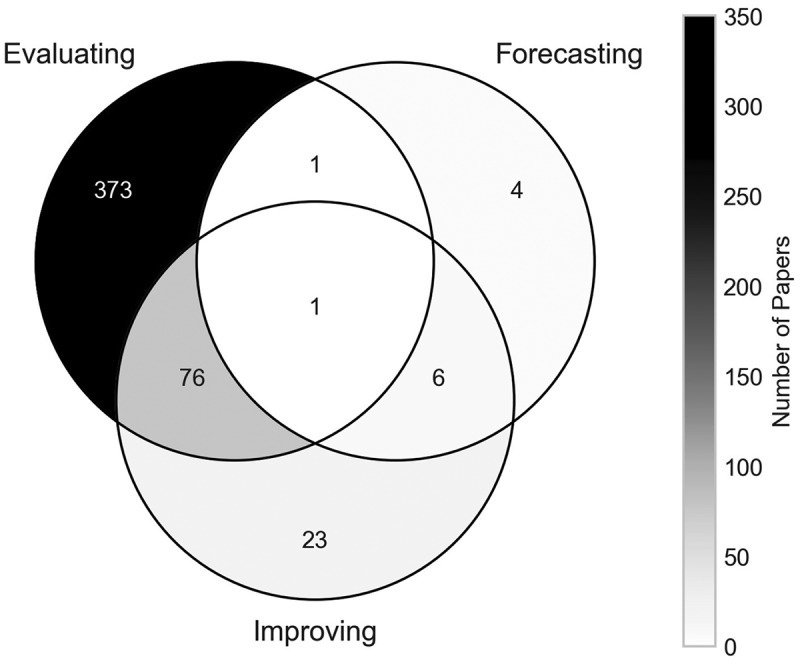
Number of papers by research aims.

Figure 12 presents the breakdown of papers by their research aims. A total of 451 papers (91.67%) aim to evaluate the orthopaedic setting or treatment in some way. Examples of evaluating aims identified include evaluating the cost-effectiveness of treatments (Mather et al., 2014) and evaluating the current system set-ups of orthopaedic clinics (Rau et al., 2013). The evaluating aim is commonly paired with the improving aim too, being combined in a total of 76 papers. An example of such is Weerawat et al. (2013), which used simulation to evaluate the current system design of an orthopaedic department, but also ran scenarios of different designs and schedule changes in an effort to improve the service’s performance.

On the other hand, forecasting has been identified as a marginal area of the literature within the scope of this review, appearing in just 11 papers (2.24%) in total, and only four as the sole research aim. Forecasting was the sole research aim of Barber and López-Valcárcel (2010), where a system dynamics simulation was used to forecast the future need for medical specialists in Spain. The limited number of identified papers that focused on forecasting could potentially be due to our exclusion of machine learning techniques from the search string. One of the primary applications of these techniques is to be employed as predictive tools for future events or outcomes.

**Figure 13.**
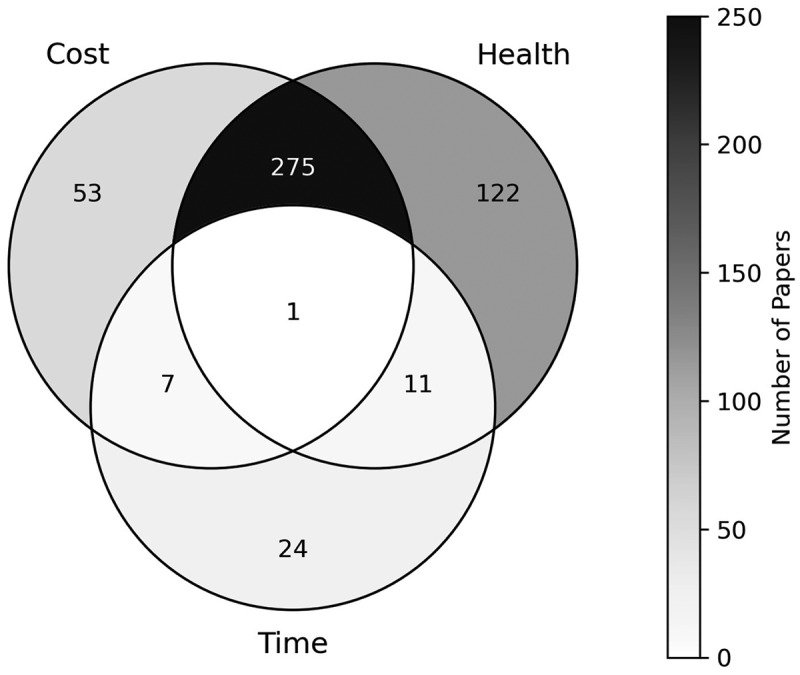
Number of papers by their research outcomes.

In terms of the outcome areas considered by each paper, the results are presented in Figure 13. Health is the most frequently occurring outcome within the papers, appearing in a total of 409 papers (83.13%). This is followed fairly closely by cost outcomes, which appear in 336 papers (68.29%). Interestingly, the most frequently occurring combination of all outcomes is the combination of cost and health outcomes, encompassing 275 papers (55.89%). This is due to the large quantity of papers that perform cost-effectiveness analyses or cost-utility analyses, where the papers will typically report a health-reported outcome such as QALYs or Disability Affected Life Years (DALYs), alongside the cost-reported outcome such as the Incremental Cost-Effectiveness Ratio (ICER).

Time outcomes are considered to a far lesser extent in the literature than the other two outcomes, with just 42 papers (8.54%) reporting such outcomes. This is perhaps surprising, considering the wide ranges of OR/MS applications to patient scheduling, and in particular operating theatre scheduling, whilst waiting times too, are frequently used as a key performance indicator within queueing and simulation models. Consequently, this too means that there are limited papers that consider either or both cost and health outcomes combined with time outcomes. In fact, just one paper considers all three outcome areas as part of its research, this being a decision analysis model that reports on complication rate and patient satisfaction (health), patient length of stay (time) and associated costs of home-visiting nurses (cost) (Ponzio et al., 2016).

Relating to the outcomes are the functional areas. The majority of publications consider some form of cost analysis, with 266 papers (54.07%) conducting a cost-effectiveness analysis of a treatment or procedure, making it the most recurrent functional area. This is followed up by papers that focus on making medical decisions, with 79 papers, 76 of which include the Delphi method as either a primary or secondary OR/MS method. Outside of cost-effectiveness, cost-utility, and cost analyses, as well as medical decision papers, all other functional areas have less than 20 instances each in the analysis, or less than 10% of the cost-effectiveness analysis total.

**Figure 14.**
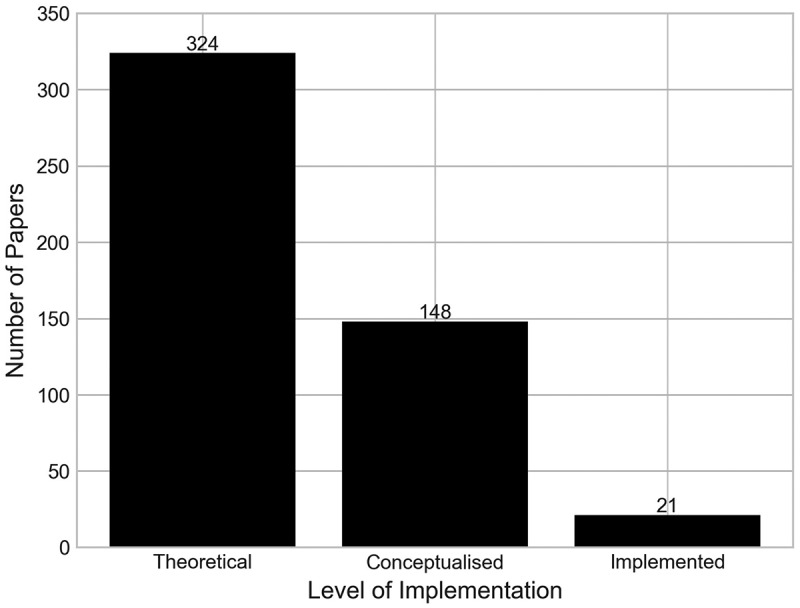
Number of papers by the level of implementation in a real world setting.

Figure 14 shows the number of papers by each implementation level. It should be noted that a single paper is classified as both conceptualised and implemented, since it reports on the development of several projects at two hospitals in Scotland (Van der Meer et al., 2005). Three of the projects within the paper report successful implementation of results at the orthopaedic department in one of the hospitals, whilst the other two projects had not yet been implemented but did involve stakeholder input.

We found that 472 papers (95.93%) either reported that the model was not implemented in practice, or did not make reference to the model’s implementation at all. In fact, 324, or almost two-thirds of the papers, were classified as theoretical, having not worked with an identified client organisation in any capacity.

In total, 4.27% of papers have reported the implementation of their research outcomes in practice. Thus, within orthopaedics, it can be said that the reporting on model implementation is on the whole low, supporting previous reviews of operational research citing this as a long-standing issue and one that has seemingly not improved (Brailsford, 2005; Brailsford et al., 2009; Fone et al., 2003).

**Figure 15.**
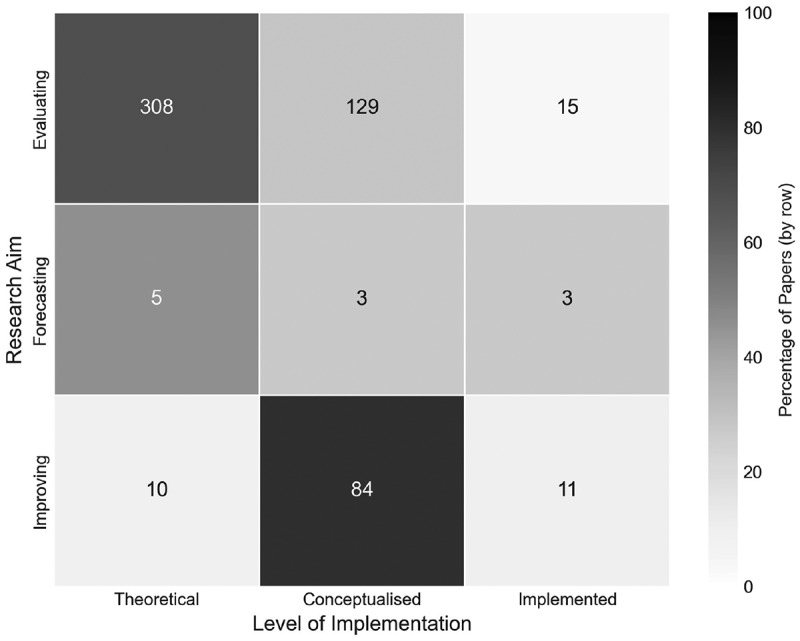
Number of papers by their research aims and level of implementation.

Figure 15 shows a cross-analysis of the number of papers by their research aims, and level of model implementation. Across the papers classified as evaluating, the majority of which are also classified as theoretical (69.62%), followed by a large number classified as conceptualised (27.27%), and very limited levels of implementation.

Interestingly, papers classified as improving have a higher proportion of also being conceptualised (80.77%), with the rest of the papers split between being theoretical and implemented. Combining conceptualised and implemented, 91.35% of papers classified as improving have reported some level of stakeholder interaction, being suggestive that stakeholders are more interested in improving the design and performance of healthcare systems and treatments than evaluating or forecasting. This could however be limited by the fact that the majority of papers within this analysis solely perform a cost/health evaluation of an orthopaedic treatment and do not report on any practical implementation.

How orthopaedic care settings and treatments have been modelled will be looked at in the next sub-section, focusing on the methodologies used by authors in conducting their research.

### 4.4. How has it been modelled?

**Figure 16.**
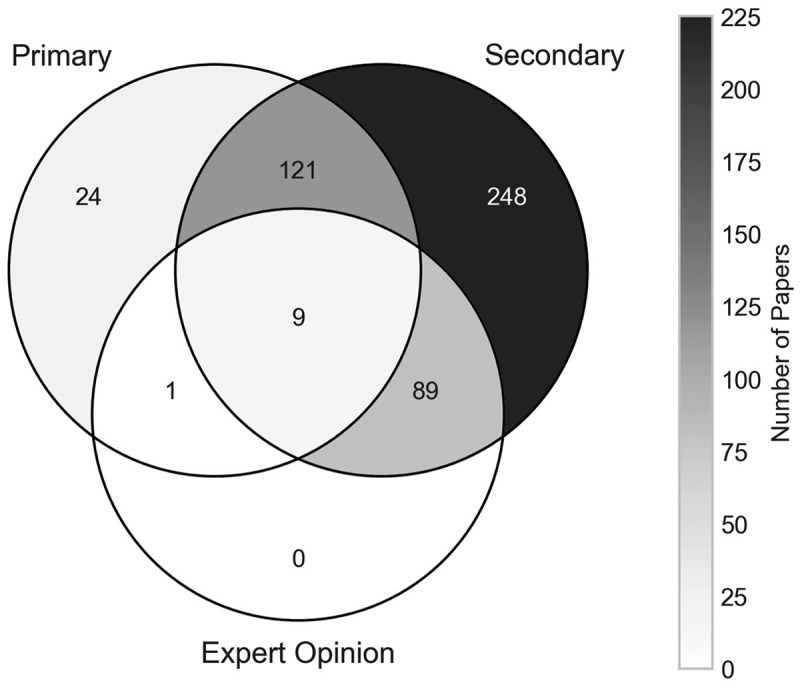
Number of papers by how the data was obtained.

Figure 16 presents the breakdown of papers by how the data used in them was sourced. Of the studied literature, 467 papers (94.92%) utilised secondary data in some way, making it substantially the largest of the three data sources. In fact, 248 papers (50.41%) used only secondary sourced data within their models. Also, note that 92.5% of all the papers that used secondary data in some capacity performed an evaluation as their research aim.

Whilst 155 papers (31.50%) use primary data of some type, this is considerably less than secondary data and is usually paired with secondary data or expert opinion in some way, leaving just 24 papers (4.88%) using primary data as their only data source. All of those 24 papers using primary data as their only source featured stakeholder involvement of some level, whether conceptualised or implemented. For the 99 papers that utilised the opinion of experts in the field, 98 of these combined it with secondary sourced data in some way, with 89 combining purely with secondary data. No papers relied solely on expert opinion for their research, suggesting that the OR/MS methods used are quantitative in nature, and reliable data sources from primary or secondary sources are vital to the work.

Table 2 outlines a cross-analysis of a paper’s level of reported implementation against how the data used was sourced. Note that the sum of each column within the table is not equal to the sum within Figure 16, due to Van der Meer et al. (2005) having been classified as having two levels of implementation. This was previously discussed in Section 4.3. Papers that reported implementation of results were split between primary and secondary sourced data, slightly in favour of primary data. There were also no papers that used primary sourced data that were also classified as theoretical, likely due to these papers obtaining that primary sourced data from some kind of stakeholder or organisation’s approval. At the same time, no implemented papers used any data from expert opinions. This may be that the estimates provided by the experts are not trustworthy or rigorous enough to be implemented, or that experts are more inclined to give their estimates for purely theoretical papers. Whilst on the other end of the spectrum, theoretically proposed papers overwhelmingly favour secondary sourced data, with 330 of the 331 theoretic papers using it in some capacity. A total of 87 theoretical papers also used expert opinion, whilst just 15 used primary sourced data, the lowest amount and percentage using primary data across the three levels of implementation.

**Table 2.** Number of papers by their level of implementation and how the data was sourced.

	Data Source		
	Primary	Secondary	Expert Opinion
Theoretical	0	324	87
Conceptualised	138	127	12
Implemented	18	17	0

**Figure 17.**
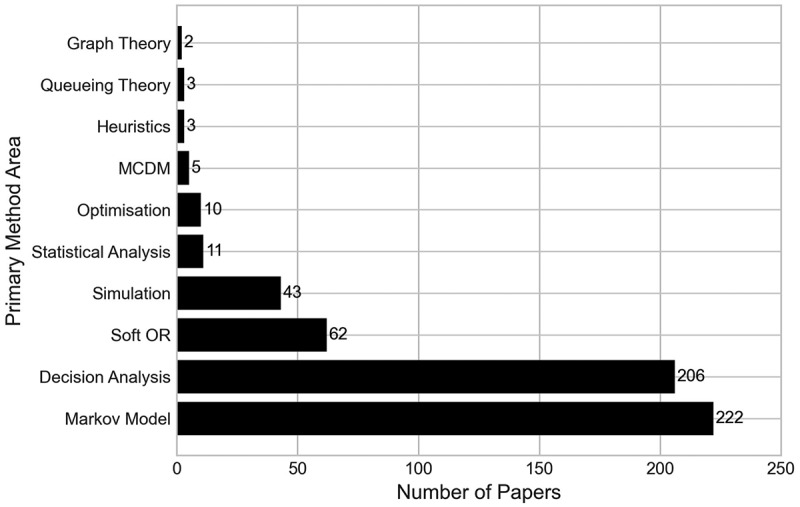
Number of papers by their primary OR/MS method area.

Figure 17 depicts how frequently the OR/MS method areas appear in the literature that was found. The most common OR area used in modelling was Markov models, found in 222 papers (45.12%). This is closely followed by decision analysis models, which featured in 206 papers (41.87%). Half of the methods (graph theory, queueing theory, heuristics, MCDM, optimisation) have been utilised in less than ten papers, with graph theory and queueing theory the joint lowest, with two applications. Initially, this number for queueing theory papers was thought surprising, but compared to the number of simulation papers, it could be that the nature of T&O modelling is more suited to more complex models (such as discrete-event simulation) than queueing theory.

A further breakdown of papers by the OR/MS method(s) applied in them is given in Table 3. The table presents all OR/MS areas found within the analysis, alongside the corresponding methods used within that area, as well as the total number of papers using that method (*N*). For the Markov, decision analysis and soft OR papers, their categories are made up entirely or almost entirely by Markov models, decision trees and the Delphi method, respectively. An array of simulation methods have been utilised across papers, to varying degrees, with 32 of the 43 simulation papers using DES in some capacity, but just two papers each for ABM and system dynamics.

**Table 3.** Breakdown of papers by their primary OR/MS area, as well as the frequency of methods used within each OR/MS area. It should be noted that some papers applied more than one primary OR/MS method in their paper.

OR/MS Area	*N*	Method	*N*
Markov Models	**222**	Markov models	222
Decision Analysis	**206**	Decision tree	204
		Break-even analysis	2
Soft OR	**62**	Delphi method	62
Simulation	**43**	Discrete-event simulation	32
		Monte Carlo simulation	6
		Agent-based model	2
		System dynamics	2
		Microsimulation	2
		Mathematical model	1
Statistical Analysis	**11**	Statistical analysis	11
Optimisation	**10**	Mixed-integer linear program	8
		Integer program	2
		Goal programming	1
		Knapsack model	1
		Particle swarm optimisation	1
MCDM	**5**	Analytic hierarchy process	5
Heuristics	**3**	Genetic Algorithm	1
		Neighbourhood search	1
		Scatter search	1
		Tabu search	1
Queueing Theory	**3**	Queueing model	3
Graph Theory	**2**	Social Network Analysis	2

In total, 73 papers (14.84%) made use of more than one OR/MS area within their research. Of these, 63 utilised a combination of Markov models with decision analysis models, commonly as part of a cost-effectiveness analysis or cost-utility analysis, making them by far the most combined areas.

Outside of mixed-method Markov-Decision analysis models, applications of mixed-methods are limited. Of other common mixed-method models, four papers used simulation-optimisation models, all of which combined DES with at least one optimisation method (goal programming (Ltaif et al., 2022); integer program (Persson & Persson, 2010); knapsack model and mixed-integer linear program (Saadouli et al., 2015); mixed-integer linear program and particle swarm optimisation (Vahdat et al., 2019)). Three of these look at patient surgical scheduling and flow (Ltaif et al., 2022; Persson & Persson, 2010; Saadouli et al., 2015), whilst the other looks at creating an efficient facility design of an outpatient department (Vahdat et al., 2019). Additionally, there is just one use of a hybrid simulation model which uses DES and ABM to improve patient waiting times in an orthopaedic outpatient department by implementing dynamic scheduling rulings on a tactical level (Kittipittayakorn & Ying, 2016).

**Figure 18.**
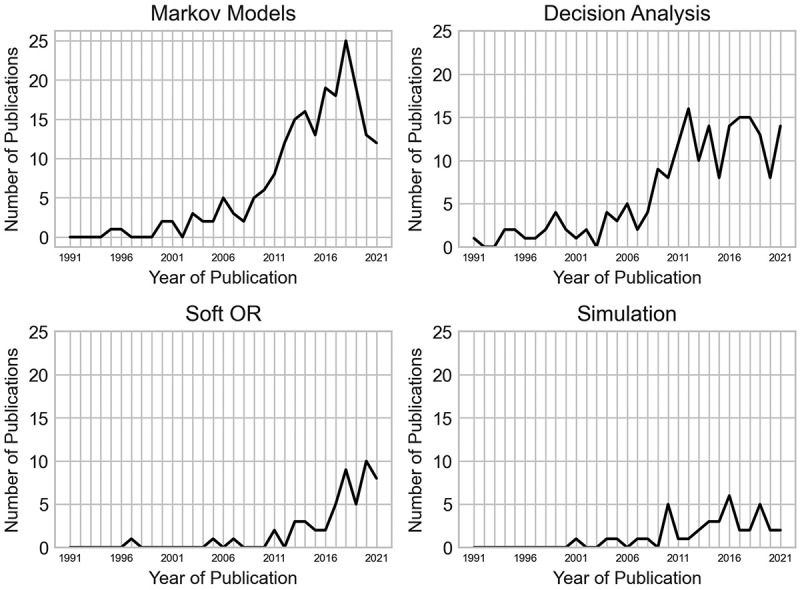
Number of papers by their year published for the four most applied OR/MS areas (Markov models, decision analysis, soft OR, simulation).

For the four most common OR/MS areas in orthopaedics (Markov models, decision analysis, soft OR, simulation), we have provided a breakdown of how the frequency of published papers per year has changed through the years in Figure 18. Markov and decision analysis models were amongst the earliest, and most popularly used methodologies in orthopaedics, with Markov models largely following the increasing trend of all methods applied as previously shown in Figure 2. However, the popularity of decision analysis models has plateaued since 2012, with soft OR methods quickly approaching it in terms of the number of papers published per year. Soft OR methods have experienced a publication boom since 2016, with 85.96% of papers using this approach being published since the start of that year. Simulation methods are also gradually becoming more utilised, though the increasing trend is slower than that of Soft OR, and as previously discussed, this is largely driven by the use of discrete-event simulation.

**Figure 19.**
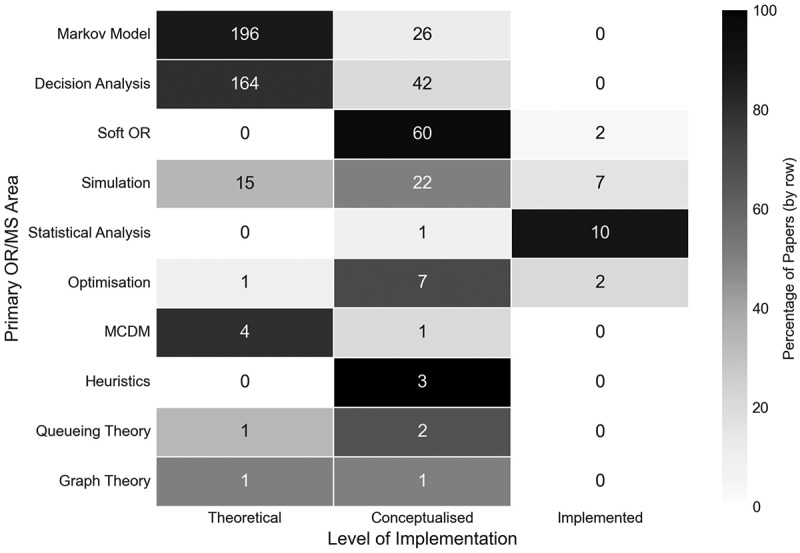
Number of papers by their primary OR/MS method area and level of implementation.

In terms of the level of implementation from the primary OR/MS method, we found that the two most common methodologies, Markov models and decision analysis, were very largely classified as theoretical, and neither in fact had any results reported as being implemented, as shown in Figure 19. On the other hand, certain areas saw much higher rates of stakeholder involvement and/or model implementation. All three papers that used heuristics were classified as conceptualised, with these papers working with outpatient departments (Ltaif et al., 2022; Vahdat et al., 2019), and surgical teams (Ebadi et al., 2017). Of the ten optimisation papers, nine featured stakeholder involvement of some degree, with seven of these being conceptualised, and two implemented in practice. No Soft OR methods have been classified as theoretical. We elected to classify these as conceptualised, as they all feature panels of medical experts convening to produce medical guidelines, unless they explicitly report on practitioners using the results in practice, in which case they are classified as implemented, as two are. Further to this, ten of the eleven statistical analysis papers are classified as implemented. All ten of these feature the Delphi method as a secondary OR/MS method and provide a statistical analysis of the results of using this soft method in the real world. The full results of the implementation and primary method cross-analysis are shown in Figure 19.

**Figure 20.**
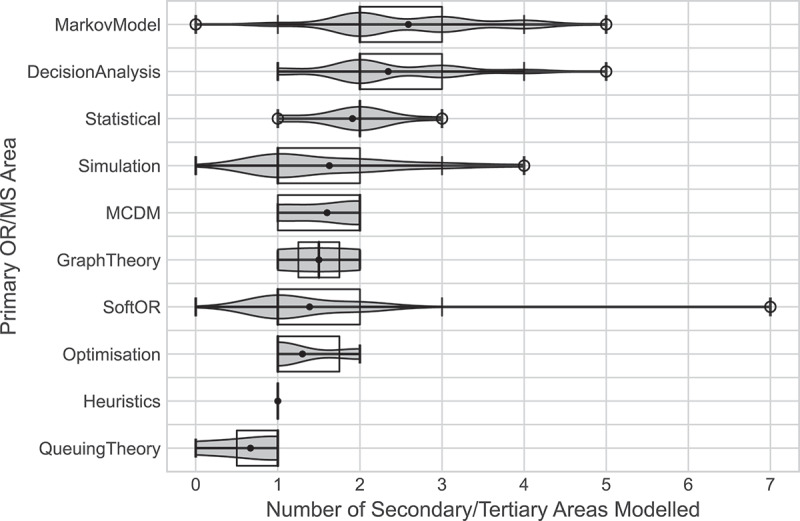
Violin plots showing the distribution of the number of secondary/tertiary care areas modelled by each primary or area.

Within secondary and tertiary care settings, which heavily featured throughout the analysis, some OR/MS methods modelled the patient care pathways more holistically than others, as shown in Figure 20 which shows the distribution of the number of secondary/tertiary care areas modelled by each primary OR area. Markov models and decision analysis models considered more areas within the pathway than other models, modelling on average more than two areas. Some papers using these methodologies modelled up to five parts of the pathway. Generally, these Markov models and decision analyses considered cost analyses of some type, or health-utility analyses, and tended to model the passage of the patient through a series of clinical events as their condition progressed, allowing for a wider modelling scope of the pathway.

Other papers modelled a much narrower scope of the secondary/tertiary care pathway. Queueing theory considered less than one pathway area on average, owing to one of the three queueing theory papers being the paper by Hauser et al. (2021) that modelled a manufacturing process of orthopaedic implants. All other methodologies modelled between one and two areas on average. The lowest of these were heuristics, each of which only modelling one area of the pathway; optimisation, which had either one or two areas modelled, where the three papers that considered two areas focused solely on the perioperative process; soft OR, which generally focused on one area, but also saw the most modelled areas included. The one paper that used soft OR methods to model all secondary/tertiary areas was classified as such as it considered developing best practice guidelines for practitioners to use, which would be applicable throughout the pathway (Bini & Mahajan, 2016).

**Figure 21.**
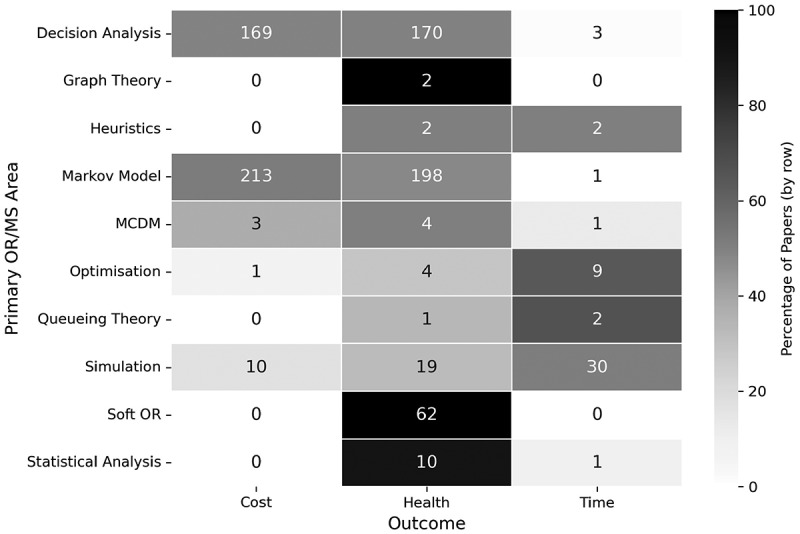
Number of papers by their research outcomes and primary OR/MS method.

Figure 21 gives the breakdown of papers by their primary OR/MS methodology and the outcome of their research. Decision analysis and Markov models focused primarily on cost and health-related outcomes, with these methods being the key drivers of cost research outcomes across all methodologies. A fair proportion of all primary methods studied health-related outcomes in some way, with soft OR and graph theory exclusively considering them. Health-reported outcomes may be more difficult to quantitatively measure, so the use of a qualitative approach such as the Delphi method may be preferential.

Time-related outcomes were mostly the result of research that used simulation or optimisation within papers, typically to assess or improve the timing efficiencies of a department or wider hospital in some way. A successful implementation of this can be seen in Neumann et al. (2020), where discrete-event simulation was used to support the implementation of improved operating room set-ups for orthopaedic surgery.

The application of OR/MS methods as secondary, or subsidiary, methods was also analysed. A total of 380 (77.23%) of the 492 papers studied in this analysis have been classified as having a secondary OR/MS method. Within the simulation classification, one used discrete-event simulation, six used a microsimulation, 123 used Monte Carlo Simulation, and one other used an unnamed type of simulation. Probabilistic sensitivity analysis was the most-utilised secondary method, appearing in 363 papers (73.63%). Of the 123 Monte Carlo simulation papers, 118 used Monte Carlo simulation as a method of probabilistic sensitivity analysis. All six papers that used microsimulation as a secondary method also used it as a means of performing probabilistic sensitivity analysis.

## 5. Discussion

We provide an explicit, reproducible search methodology and taxonomy for healthcare professionals and OR/MS researchers to use and adapt for the identification and classification of relevant papers. As in other literature reviews, we recognise the caveats posed by this method of reviewing the literature. The list of search terms used within the search string for both OR/MS methods and orthopaedic terms was broad, but not exhaustive, so it is possible not all relevant papers were found and thus the scope of this review is limited to the search terms used. The scope of our review is also limited to the Scopus database, whereas including searches on other electronic databases such as Medline or PubMed may have enhanced the search. We also recognise that although providing a broad cross-section of OR/MS applications, the six JCR categories we used in the original search did not provide an exhaustive list of all papers, with many applications found in papers outside of the journals in these categories. The effects of this were mitigated, however, through the use of the forward and backward search approaches, and we found the majority of papers were identified in our initial search, highlighting the strength of the string.

The taxonomy was designed to collate classification variables from other literature reviews of OR/MS methods applied to healthcare, with the addition or restructuring of several more to give a thorough overview of the details of each paper identified. The taxonomy is generalisable enough to be adapted to future OR/MS healthcare reviews of other areas of healthcare.

Alongside the taxonomy, we have ensured complete transparency in the results of this literature review by uploading the CSV file containing all paper classifications, as well as the Jupyter Notebook file for analysis, to Zenodo, as discussed in Section 3.2. This provides readers with the opportunity to replicate analysis on our findings, while also allowing for the work to be expanded and updated.

In all, this review uncovered valuable findings of the current extent of OR/MS applications to orthopaedic settings and treatments. This section adds some discussion around the major points of interest resulting from our analysis, answering the subsection questions posed in Section 4, and outlining areas of future work researchers may wish to consider.

### 5.1. Who has done the modelling?

The papers included in this review featured publication dates spanning 32 years, and highlight the richness of research in certain areas of orthopaedic care and methodologies, but also point to several opportunities for further development and study. In general, it was found that the trend of published papers is increasing, with valuable input from all six of the JCR Categories that we derived the papers from.

Papers generally applied their work to countries within North America and Europe, though this could be biased by the scope of this review, being limited to English language papers. We considered that this might be expected, as areas of lower SDI scores, as in North America and Europe, have a higher burden of orthopaedic disorders. It may also be the case that in general, research funding is more available in more prosperous countries, who may choose to spend the funding on themselves rather than elsewhere. However, the threat ageing populations pose on healthcare systems is a global phenomenon, and the worldwide prevalence of orthopaedic conditions is increasing, so future research in areas of higher SDI scores could well be vital. This may be particularly true for Africa and South America, which had the lowest number of publications.

### 5.2. What has been modelled?

Secondary care was the most frequently modelled care area across the papers found in our analysis, appearing in 445 publications. Primary care had limited applications, appearing in just 18 papers. Since orthopaedic surgeries are carried out in secondary or tertiary care, this is to be expected.

Successful care mapping should document the journey of a patient through their condition or healthcare system. We took a more in-depth look at secondary and tertiary care to assess the extent to which the patient care pathways were modelled in these papers. In total, 217 papers modelled two areas of the pathway, with 153 of these considering the perioperative process (surgery and post-surgery), which are closely woven together anyway. Decision analysis and Markov models were most successful in considering wider scopes of the pathway, whilst all other methods modelled on average less than two areas of the pathway per paper. In order to assess the flow of patients from referral to follow-up, it would be imperative to model the holistic orthopaedic pathway to understand the full picture, which could provide valuable insights into the system for demand and capacity planning. This is useful for simulation models since this review found limited interactions between elective outpatient and surgical activities.

Most orthopaedic specialties were well represented within the results, with knee, hip and spine conditions being the most commonly modelled, which was perhaps expected with treatments for these being among the most common, and expensive procedures, making it imperative that their costs are minimised, but effectiveness maximised. As discussed in Section 5.1, considering the growing global prevalence of orthopaedic conditions and the ageing population, it is likely that treatments for less common orthopaedic conditions may require greater attention. Falling quite short of the totals of most of the specialties though were papers that modelled orthopaedic departments (29 publications), or hospital systems (9 publications). All but two papers modelling a department or hospital system considered resource capacity planning of some degree, which ties into the papers with reported planning decision levels, which we also found to be low (43 publications). Comparing this to other reviews, Aspland et al. (2021) found that of their 175 identified papers on clinical pathway modelling, 82 (46.86%) considered planning decision levels. Furthermore, in Williams et al. (2021), p. 56 papers were found considering planning decision levels of the 62 identified for care planning for frail and elderly patients.

The dimensioning and planning of system resources and capacity levels is vital to allow for the efficient operations of a healthcare service, to ensure low waiting times and higher quality of care. With the threat of increased demand in the future, as well as the current backlog many elective care systems face, resource capacity planning should be at the centre of the minds of healthcare providers. Successful care planning should take into consideration the range of these planning levels, from long-term (strategic) care, to day-to-day operations and planning (operational). Our work highlighted that within the already limited subset of papers considering resource capacity planning, the majority focused on mid-to-long-term planning decisions (strategic and tactical), whilst there were few applications of day-to-day planning.

### 5.3. Why has it been modelled?

The level of implementation of research in a real-world setting was found to be low. This has been an issue previously reported in reviews of other OR/MS applications (Brailsford, 2005; Brailsford et al., 2009; Fone et al., 2003). As in Brailsford et al. (2009), we too found that a large number of papers did reach the conceptualised stage of implementation, though the number of theoretical papers still dwarfs the other categories. Whether the papers classified as theoretical were intended to be implemented is unknown, as this was largely left ambiguous. Simulation and optimisation papers had the highest proportion of implemented results, though the total count of both of these categories is fairly low, especially optimisation. Qualitative methods such as soft OR were more likely to reach the conceptualised stage of implementation than others. Reporting on the implementation, or planned implementation of future research could aid healthcare providers and stakeholders in seeing the benefits of OR/MS techniques to helping to improve services.

Among the examined methods in the scope of this review, one notable finding was the limited number of papers classified with forecasting as their research aim. Specifically, only twelve studies were identified in this category. This was particularly noticeable when compared to much greater quantities of papers that aimed to evaluate (451 papers), or improve (105) the focal point of the study. As previously discussed in Section 4.3, this could be limited by the fact that we did not include machine learning techniques in our search string. In the event that this is an area that has received limited research, it can be concluded that forecasting demand is critical in ensuring the right resource and capacity dimensioning is in place at orthopaedic departments and facilities, which could help in improving the quality of care for many. This is particularly true now due to the effects of the COVID-19 pandemic on healthcare systems, and in particular orthopaedic departments, which have faced strenuous backlogs to elective care services. Forecasting future demand, the return of previous unmet demand during periods of public health measures, or the degeneration of patients waiting on these lists could be a key tool in aiding the recovery of these systems. This review identified no papers that sought to model the ongoing effects of the pandemic on orthopaedic health systems.

In contrast, there was a considerably larger amount of papers that aimed to evaluate or improve a system or treatment, especially those seeking to evaluate. Evaluating the performance of something can be key to identifying its efficiencies and whether or not there is scope to improve it further. This is particularly true when we see the number of papers that sought to both evaluate and improve something, which was by far the largest combination of research aims found.

Tying into the limited number of resource capacity planning papers previously discussed, we found that time-related outcomes were also, by far the least modelled outcome measure. Improving time-related outcomes such as patient waiting times, resource utilisation and patient/staff scheduling can be key in ensuring the system runs at optimal, or close to optimal performance.

### 5.4. How has it been modelled?

The bulk of the literature stems from Markov models and decision analyses to perform cost-effectiveness analyses, cost-utility analyses, cost analyses, or health-utility analyses. In total, 365 papers used one, or both of these methods, highlighting the popularity of these methodologies, especially within the functional areas stated above. Soft OR methods, but in particular the Delphi method, are becoming increasingly used in orthopaedics, allowing clinical professionals to devise nationally, or internationally agreed upon medical decision guidelines.

Some methodologies were utilised to far lesser degrees though, highlighting the disparities in methodological applications. Given the strong potential of optimisation methods to appointment scheduling (Van den Bergh et al., 2013), and especially historically to operating theatre scheduling (Cardoen et al., 2010), it was perhaps surprising to find just eleven applications of this method throughout our literature search, being suggestive that this is an under looked area of research within the specialty. Just three papers applied heuristics or metaheuristics to their work as well, which can be used to strengthen optimisation models.

Despite simulation being the fourth most used primary OR/MS area in this analysis, 33 of the 44 simulation papers used discrete-event simulation, and an extra six used Monte Carlo simulation. Agent-based models and system dynamics models had just two applications each. System dynamics models in particular could be useful to assess demand and capacity on wider scopes, such as for assessing the interactions between multiple areas of the integrated care system (e.g., primary, secondary and community services), or the interactions between orthopaedic departments and multiple other areas of a hospital.

The limited number of optimisation and simulation approaches used may link with the lack of papers that modelled time-related outcomes, since these outcomes would be key performance indicators for problems relating to operating theatre scheduling and waiting list management. Additionally, only three papers utilising queueing theory were found in this review, which could also be beneficial in modelling time-related outcomes. Given the stochastic nature of orthopaedic treatments, with some patients requiring emergency appointments, and the current and future pressures of waiting list sizes, these methodologies have the potential to gain increased attention in the future.

There were some methodologies included in our Scopus search string that yielded no results too. Despite searching for game theoretical models, our original Scopus search returned no results of this. It could be that this methodology is not suited to the focus of orthopaedic modelling, but it may be an area of future consideration for researchers. We identified a total of 62 papers using soft OR/MS methods; however, all of these papers utilised the Delphi method in their approach. This is in spite of the inclusion of other soft OR methods being included in the search string, such as drama theory and strategic choice analysis. We previously discussed in Section 3.1, the omission of machine learning terms from our search string, and this analysis. We would like to reiterate that there is the potential for further study of the application of these methods to orthopaedics.

The application of mixed-methodologies was found to not be particularly diverse. We found that 63 of the 73 papers using more than one OR/MS combined Markov models with decision analysis models, leaving just 10 other combinations of other methods, four of which using simulation and optimisation approaches, and only one paper using a hybrid-simulation approach. Many healthcare systems face problems that are both diverse and complex in nature, and not ones which a single method may be able to effectively solve alone. Mixing OR/MS methods provides a useful tool in mitigating the weaknesses of single methods (Jackson & Keys, 1984). Further to this, we classified the papers using mixed-methodologies according to the interactions between the methods as in Brailsford et al. (2019). However, our findings are consistent with Brailsford et al. (2019), in that it was difficult to identify the level of interactions between the methods in most papers, as they were unclear in their description of the extent of this.

## 6. Conclusion

The aim of this paper was to quantify and taxonomise the current breadth of OR/MS methods applied to orthopaedics treatments and settings in published literature. We first outlined our methodology for classifying papers according to their general, medical and methodological contexts (Section 2). The approach and criteria for searching for and including papers for this analysis was then described, to allow researchers to reproduce this work, and systematically search for relevant papers (Section 3). The papers that achieved our inclusion criteria were then classified according to our outlined taxonomy, and we presented the results of this analysis in an effort to identify the key research trends, streams, and gaps (Section 4).

The findings of this paper highlight certain areas of advised focus that future research may consider, as discussed in Section 5. The key considerations for the directions of future research are summarised as follows:

- The modelling of multiple care areas, and in particular the holistic pathway within secondary/tertiary care, could be considered, to allow healthcare providers to ascertain a more complete picture of the patient’s journey, and the effect of patient flow through the system. ‘
- Increased focus on resource capacity planning, in particular day-to-day operations, may be considered to help ensure systems operate as efficiently as possible and alleviate any bottlenecks in the system. The most noteworthy gap found in this context was for time-related outcomes.
- Increased attention to applying optimisation, simulation and queueing theory methodologies that can be used to tackle problems relating to appointment scheduling and waiting list management, where time-related outcomes are key performance indicators.
- There is space in the literature for increased applications of mixed-methodologies, to provide more powerful tools for optimising the performance of orthopaedic systems and treatments.
- The reporting of model implementation is low and may be improved where possible to highlight the success of OR/MS model applications in the real world.
- Given the global phenomenon of an ageing population, the increased applications of OR/MS methods in orthopaedics, particularly in countries where such applications have been limited thus far, could yield significant benefits.

To conclude, orthopaedic health systems and treatments are vital to patients suffering with musculoskeletal conditions by aiding their recovery and offering an improved quality of life. With the current stresses these systems are under, as well as the impending threat of increased demand, these systems face being stretched to the limit.

The results of this review have provided researchers and healthcare professionals with a comprehensive overview of previous OR/MS applications to orthopaedic treatments and care settings, as well as directions for future research. OR/MS methods have been shown to be incredibly valuable tools within this area of care and will continue to be going forward, perhaps even more so in helping healthcare services optimise their systems and treatments for future demand.

## Supplementary Material

Supplemental Material
