# Supplementary material for: Fractured systems: a literature review of OR/MS methods applied to orthopaedic care settings and treatments
Source: Health Syst (Basingstoke). 2023 Oct 9;13(3):151–76. doi: 10.1080/20476965.2023.2264348 (PMC11338206; doi:10.1080/20476965.2023.2264348)
Supplement: Supplemental Material [file THSS_A_2264348_SM6665.pdf]

Appendix A. Search Terms

**OR/MS Methods**	
Agent based modelling	Branch and bound
Branch and price	Column generation
Confrontation analysis	Constraint programming
Decision tree	Delphi method
Discrete event simulation	Discrete optimisation
Drama theory	Dynamic programming
Evolutionary algorithm	Frog-leaping algorithm
Game theory	Genetic algorithm
Goal programming	Heuristics
Integer programming	Linear programming
Markov chain	Markov decision model
Markov model	Mathematical model
Mathematical programming	Memetic algorithm
Metaheuristics	Mixed integer programming
Monte Carlo simulation	Multi-objective programming
Network analysis	Optimisation
Problem structuring	Quadratic programming
Queuing	Queueing
Robustness analysis	Scatter search
Simulated annealing	Soft operational research
Soft systems methodology	Stochastic analysis
Stochastic modelling	Stochastic processes
Stochastic programming	Strategic choice analysis
Strategic options development and analysis	Systems dynamics
Tabu search	

Table A1. List of OR/MS methods used in the search

Orthopaedic Term	
Acetabuloplasty	Alveolar bone grafting
Amputation	Arthrodesis
Arthroplasty	Arthroscopy
Artificial limbs	Athletic tape
Bone lengthening	Bone transplantation
Braces	Canes
Cementoplasty	Cruciate ligament reconstruction
Crutches	Disarticulation
Diskectomy	Fixation devices
Fixators	Foot orthoses
Fracture fixation	Fracture reduction
Hemiarthroplasty	Hemipelvectomy
Ilizarov technique	Intervertebral disc chemolysis
Joint capsule release	Kyphoplasty
Laminectomy	Laminoplasty
Ligament reconstruction	Limb salvage
Meniscectomy	Occlusal splints
Orthogonal surgical procedure	Orthopaedics
Orthotic device	Osteogenesis
Osteotomy	Sinus floor augmentation
Spinal fusion	Synovectomy
Tendon transfer	Tendon-bone grafting
Tendosis	Tenotomy
Total disc replacement	Traction
Vertebroplasty	Viscosupplementation
Walkers	

Table A2. List of T&O terms used in the search

Appendix B. Search String

The complete search string used for the Scopus search is listed below. It includes all OR/MS terms, orthopaedic terms, journal ISSN or EISSN codes from the relevant JCR categories, and the restriction to the English language, as outlined in Section 3.

```
TITLE-ABS-KEY(("Agent based model*" OR "Agent based simulation" OR
"Branch and bound" OR "Branch and price" OR "Column generation"
OR "Confrontation Analysis" OR "Constraint program*" OR "Decision
tree" OR "Delphi method" OR "Discrete event simulation" OR "Discrete
optimi*" OR "Drama Theory" OR "Dynamic program*" OR "Evolutionary
algorithm" OR "Frog-leaping algorithm" OR "Game theor*" OR "Genetic
algorithm" OR "Goal program*" OR "Heuristic$" OR "Integer program*" OR
"Linear program*" OR "Markov chain" OR "Markov decision" OR "Markov
model" OR "Mathematical model" OR "Mathematical program*" OR "Memetic
algorithm" OR "Metaheuristic$" OR "Mixed integer program*" OR "Monte
```

carlo simulation" OR "Multi-objective program*" OR "Network analysis" OR "Optimi?ation" OR "Problem structuring" OR "Quadratic program*" OR "Queuing" OR "Queueing" OR "Robustness analysis" OR "Scatter search" OR "Simulated annealing" OR "Strategic Choice Analysis" OR "Strategic Options Development and Analysis" OR "Stochastic analysis" OR "Stochastic modelling" OR "Stochastic processes" OR "Stochastic program*" OR "Soft Operation* Research" OR "Soft Systems Methodology" OR "System$ dynamics" OR "Tabu Search") AND ("orthopaedic$" OR "orthopedic$" OR "acetabuloplasty" OR "amputation" OR "disarticulation" OR "hemipelvectomy" OR "arthrodesis" OR "spinal fusion" OR "arthroplasty" OR "cruciate ligament reconstruction" OR "tendon-bone grafting" OR "hemiarthroplasty" OR "total disc replacement" OR "arthroscopy" OR "joint capsule release" OR "bone lengthening" OR "Ilizarov technique" OR "osteogenesis" OR "bone transplantation" OR "sinus floor augmentation" OR "cementoplasty" OR "vertebroplasty" OR "kyphoplasty" OR "diskectomy" OR "fracture fixation" OR "fracture reduction" OR "laminectomy" OR "limb salvage" OR "meniscectomy" OR "osteotomy" OR "orthogonal surgical procedure$" OR "alveolar bone grafting" OR "synovectomy" OR "tendon transfer" OR "tenodesis" OR "tenotomy" OR "traction" OR "ulnar collateral ligament reconstruction" OR "viscosupplementation" OR "ligament reconstruction" OR "intervertebral disc chemolysis" OR "laminoplasty" OR "artificial limbs" OR "canes" OR "crutches" OR "fixation device$" OR "fixators" OR "orthotic device$" OR "athletic tape" OR "braces" OR "foot Orthoses" OR "occlusal Splints" OR "walkers")) AND (ISSN(0001-5415 OR 0001-6462 OR 0003-6870 OR 0007-8506 OR 0009-921X OR 0010-3853 OR 0013-5585 OR 0013-791X OR 0014-0139 OR 0017-9124 OR 0018-7208 OR 0018-9391 OR 0019-5413 OR 0020-1383 OR 0020-7314 OR 0020-7543 OR 0020-7721 OR 0021-9355 OR 0022-3239 OR 0022-4065 OR 0025-1909 OR 0025-5610 OR 0025-7079 OR 0026-1270 OR 0028-3045 OR 0030-364X OR 0030-5898 OR 0031-9023 OR 0038-0121 OR 0041-1655 OR 0041-1868 OR 0046-9580 OR 0085-4530 OR 0091-3847 OR 0092-2102 OR 0094-5145 OR 0140-0118 OR 0143-2087 OR 0143-991X OR 0147-7447 OR 0148-5598 OR 0156-5788 OR 0160-5682 OR 0163-2787 OR 0166-4972 OR 0167-6296 OR 0167-6377 OR 0167-6911 OR 0167-9236 OR 0168-8510 OR 0169-2607 OR 0169-8141 OR 0171-6468 OR 0190-6011 OR 0191-2615 OR 0197-5897 OR 0213-9111 OR 0217-5959 OR 0219-6220 OR 0233-1934 OR 0254-5330 OR 0257-0130 OR 0263-5577 OR 0266-4623 OR 0267-5730 OR 0268-0033 OR 0268-1080 OR 0269-9648 OR 0271-6798 OR 0272-6963 OR 0272-989X OR 0277-6715 OR 0278-2715 OR 0278-6125 OR 0294-0337 OR 0300-8207 OR 0305-0483 OR 0305-0548 OR 0305-215X OR 0309-3646 OR 0315-5986 OR 0341-2695 OR 0360-8352 OR 0361-6274 OR 0361-6878 OR 0362-2436 OR 0363-5023 OR 0363-5465 OR 0364-2348 OR 0364-765X OR 0377-2217 OR 0399-0559 OR 0733-9364 OR 0736-0266 OR 0737-6782 OR 0742-597X OR 0744-6020 OR 0748-5492 OR 0748-8017 OR 0749-0712 OR 0749-6753 OR 0749-8063 OR 0825-8597 OR 0883-5403 OR 0887-378X OR 0890-5339 OR 0890-765X OR 0891-5245 OR 0891-8422 OR 0894-069X OR 0894-1130 OR 0894-587X OR 0895-6308 OR 0898-2112 OR 0898-2643 OR 0923-4748 OR 0924-0136 OR 0924-6703 OR 0925-5001 OR 0925-5273 OR 0925-7535 OR 0926-6003 OR 0932-0555 OR 0933-3657 OR 0934-6694 OR 0934-9839 OR 0936-8051 OR

0940-6719 OR 0942-2056 OR 0949-2658 OR 0951-192X OR 0951-8320 OR
0953-7287 OR 0954-0121 OR 0957-4174 OR 0957-4824 OR 0959-3020 OR
0962-2802 OR 0962-9343 OR 0963-1801 OR 0966-6362 OR 0968-0160 OR
0969-6016 OR 0969-9988 OR 1004-3756 OR 1004-4132 OR 1012-277X OR
1017-995X OR 1020-3397 OR 1022-5536 OR 1041-0236 OR 1042-9247 OR
1049-2089 OR 1050-642X OR 1053-8127 OR 1054-8289 OR 1055-6788 OR
1057-9230 OR 1058-2746 OR 1059-1478 OR 1059-7700 OR 1060-152X OR
1062-2551 OR 1063-293X OR 1063-4584 OR 1063-8628 OR 1065-3058 OR
1067-151X OR 1067-2516 OR 1067-5027 OR 1071-1007 OR 1075-2730 OR
1076-8971 OR 1077-2618 OR 1077-5587 OR 1082-5983 OR 1083-7515 OR
1088-0224 OR 1091-4358 OR 1091-9856 OR 1094-3412 OR 1094-6136 OR
1096-9012 OR 1098-1241 OR 1098-3015 OR 1109-2858 OR 1120-7000 OR
1134-5764 OR 1170-7690 OR 1175-5652 OR 1178-1653 OR 1220-1766 OR
1268-7731 OR 1348-9151 OR 1353-4505 OR 1355-8196 OR 1356-1294 OR
1356-1820 OR 1366-5545 OR 1369-6513 OR 1386-5056 OR 1386-9620 OR
1389-4420 OR 1413-3555 OR 1413-7852 OR 1432-2994 OR 1435-246X OR
1435-5558 OR 1438-8871 OR 1446-1242 OR 1448-7527 OR 1460-4582 OR
1471-678X OR 1472-684X OR 1473-2262 OR 1473-7167 OR 1477-7266 OR
1478-4505 OR 1478-7547 OR 1478-9515 OR 1523-4614 OR 1524-1904 OR
1529-9430 OR 1532-0464 OR 1538-2931 OR 1538-8506 OR 1547-5816 OR
1549-8417 OR 1551-3203 OR 1566-113X OR 1568-4156 OR 1568-4539 OR
1572-5286 OR 1590-9921 OR 1618-7598 OR 1619-4500 OR 1684-3703 OR
1696-2281 OR 1726-4529 OR 1729-0376 OR 1741-038X OR 1741-1122 OR
1744-1331 OR 1745-3674 OR 1747-7778 OR 1748-006X OR 1748-5908 OR
1749-799X OR 1751-5254 OR 1753-1934 OR 1753-8157 OR 1757-7853 OR
1819-5164 OR 1833-3583 OR 1836-9553 OR 1862-3522 OR 1862-4472 OR
1863-2521 OR 1864-6697 OR 1865-9284 OR 1869-0327 OR 1877-0568 OR
1923-2926 OR 1932-8184 OR 1936-6574 OR 1936-6582 OR 1942-2962 OR
1943-670X OR 1947-6035 OR 2000-656X OR 2040-4166 OR 2044-5415 OR
2045-4015 OR 2046-3758 OR 2049-4394 OR 2054-8397 OR 2055-2076 OR
2151-4585 OR 2168-2194 OR 2168-4790 OR 2191-1991 OR 2192-5682 OR
2199-9023 OR 2211-8837 OR 2213-0764 OR 2214-031X OR 2291-5222 OR
2291-9279 OR 2328-8604 OR 2332-3493 OR 2380-0186 OR 2396-7544 OR
2398-6352 OR 2467-964X OR 2468-1229 OR 2472-5854 OR 2687-4784 OR
8750-7315 OR 2472-5579 OR 2047-6965 OR 1572-9389 OR 0891-7736 OR
1040-2446 OR 1382-4996 OR 2223-9170 OR 2078-5127 OR 2327-8994 OR
1049-9091 OR 1062-8606 OR 1120-9135 OR 2141-9248 OR 2224-5820 OR
2341-5401 OR 0043-9630 OR 2056-6697 OR 2045-435X OR 0125-9695 OR
0007-5140 OR 1742-3953 OR 2324-481X OR 1178-6981 OR 1751-4258 OR
1179-1365 OR 1029-1857 OR 0961-5423 OR 1091-7527 OR 1721-6915 OR
1827-1987 OR 2284-2403 OR 0093-0334 OR 2353-6942 OR 1025-9848 OR
1387-3741 OR 1178-6329 OR 1366-5278 OR 0973-1075 OR 2528-4398 OR
1368-2156 OR 1687-6415 OR 1540-580X OR 2691-3321 OR 1929-0748 OR
1553-7250 OR 0148-9917 OR 2458-8938 OR 0895-4356 OR 2042-6305 OR
0894-1912 OR 0884-8734 OR 0857-4421 OR 2040-2295 OR 2376-0540 OR
0161-4754 OR 1369-6998 OR 1178-2390 OR 0885-3924 OR 1096-6218 OR
2374-3743 OR 2330-068X OR 1759-8885 OR 0022-4391 OR 1357-633X OR
2327-6886 OR 0022-5045 OR 1729-0341 OR 1636-6522 OR 0308-0110 OR
0025-7273 OR 0142-159X OR 2081-0016 OR 2211-6923 OR 0269-2163 OR
1179-271X OR 2212-2761 OR 1942-7891 OR 1932-8087 OR 0281-3432 OR

```
1559-2332 OR 2253-1564 OR 0941-4355 OR 1040-1334 OR 0928-7329 OR
1530-5627 OR 2317-269X OR 1615-2921) OR EISSN(1471-2474 OR 1757-1146
OR 2325-9671 OR 1472-6947 OR 2214-7829 OR 2291-9694 OR 2589-7500
OR 1477-7525 OR 2227-9032 OR 2322-5939 OR 1179-1594 OR 1472-698X
OR 1478-4491 OR 1472-6963 OR 1471-2288 OR 1477-7525 OR 2227-9032
OR 2281-7824 OR 1744-1609 OR 2322-5939 OR 2214-7829 OR 2075-4426 OR
1179-1594 OR 1178-203X)) AND ( LIMIT-TO ( LANGUAGE,''English" ) )
```

Appendix C. Relevant Papers Identified in the Searches

All 492 papers used as part of the analysis, as well as the 8 existing literature reviews on health economic approaches found in the original search are included here.

Identified Papers

[1] T.F. Aae, P.-H. Randsborg, H. Lurås, A. Årøen, and Ø.B. Lian. Microfracture is more cost-effective than autologous chondrocyte implantation: a review of level 1 and level 2 studies with 5 year follow-up. *Knee Surgery, Sports Traumatology, Arthroscopy*, 26(4):1044–1052, 2018.

[2] M.P. Abdel, L.E. Miller, A.D. Hanssen, and M.W. Pagnano. Cost analysis of dual-mobility versus large femoral head constructs in revision total hip arthroplasty. *Journal of Arthroplasty*, 34(2):260–264, 2019.

[3] M.P. Abdel, L.E. Miller, S.A. Hull, A.B. Coppolecchia, A.D. Hanssen, and M.W. Pagnano. Cost analysis of dual-mobility constructs in revision total hip arthroplasty: A european payer perspective. *Orthopedics*, 43(4):250–255, 2020.

[4] H. Abdel Khalik, B. Humphries, M. Zoratti, D. Axelrod, C. Kruse, B. Ristevski, K. Rajaratnam, M. Gardner, J.-E. Tarride, and H. Johal. Reverse total shoulder arthroplasty is the most cost-effective treatment strategy for proximal humerus fractures in older adults: A cost-utility analysis. *Clinical Orthopaedics and Related Research*, 480(10):2013–2026, 2022.

[5] A. Aichmair, J.M. Burgstaller, M. Schwenkglenks, J. Steurer, F. Porchet, F. Brunner, M. Farshad, and LSOS Study Group. Cost-effectiveness of conservative versus surgical treatment strategies of lumbar spinal stenosis in the swiss setting: analysis of the prospective multicenter lumbar stenosis outcome study (lsos). *European Spine Journal*, 26(2):501–509, 2017.

[6] H. Al Hasan, C. Guéret, D. Lemoine, and D. Rivreau. Surgical case scheduling with sterilising activity constraints. *International Journal of Production Research*, 57(10):2984–3002, 2019.

[7] R.H. Albright, S. Haller, E. Klein, J.R. Baker, Jr. Weil, L., Sr. Weil, L.S., and A.E. Fleischer. Cost-effectiveness analysis of primary arthrodesis versus open reduction internal fixation for primarily ligamentous lisfranc injuries. *Journal of Foot and Ankle Surgery*, 57(2):325–331, 2018.

[8] R.H. Albright, B.J. Waverly, E. Klein, Jr. Weil, L., Sr. Weil, L.S., and A.E. Fleischer. Percutaneous kirschner wire versus commercial implant for hammertoe repair: A cost-effectiveness analysis. *Journal of Foot and Ankle Surgery*, 57(2):332–338, 2018.

[9] I.S. Aleem, P.J. Karanicolas, and M. Bhandari. Arthroplasty versus internal fixation of femoral neck fractures: A clinical decision analysis. *Ortopedia Traumatologia Rehabilitacja*, 11(3):233–241, 2009.

[10] Z.S. Ali, K. Thavorn, R. Murphy, S. Sparavalo, and I. Wong. Primary bankart repair versus arthroscopic anatomic glenoid reconstruction in patients with subcritical bone loss: A cost-utility analysis. *JBJS Open Access*, 6(4), 2021.

[11] B. Allenet, F. Parée, T. Lebrun, L. Carr, J. Posnett, J. Martini, and C. Yvon. Cost-effectiveness modeling of dermagraft® for the treatment of diabetic foot ulcers the french context. *Diabetes and Metabolism*, 26(2):125–132, 2000.

[12] J. Ament, B. Thaci, Z. Yang, E. Kulubya, W. Hsu, G. Bouma, and K.D. Kim. Cost-effectiveness of a bone-anchored annular closure device versus conventional lumbar discectomy in treating lumbar disc herniations. *Spine*, 44(1):5–16, 2019.

[13] J.D. Ament, Z. Yang, P. Nunley, M.B. Stone, and K.D. Kim. Cost-effectiveness of cervical total disc replacement vs fusion for the treatment of 2-level symptomatic degenerative disc disease. *JAMA Surgery*, 149(12):1231–1239, 2014.

[14] J.D. Ament, Z. Yang, P. Nunley, M.B. Stone, D. Lee, and K.D. Kim. Cost utility analysis of the cervical artificial disc vs fusion for the treatment of 2-level symptomatic degenerative disc disease: 5-year follow-up. *Neurosurgery*, 79(1):135–144, 2016.

[15] N. Amin, L. McIntyre, T. Carter, J. Xerogeanes, and J. Voigt. Cost-effectiveness analysis of needle arthroscopy versus magnetic resonance imaging in the diagnosis and treatment of meniscal tears of the knee. *Arthroscopy - Journal of Arthroscopic and Related Surgery*, 35(2):554–562.e13, 2019.

[16] A.M. Anderson, C. Comer, T.O. Smith, B.T. Drew, H. Pandit, D. Antcliff, A.C. Redmond, and G.A. McHugh. Consensus on pre-operative total knee replacement education and prehabilitation recommendations: a uk-based modified delphi study. *BMC Musculoskeletal Disorders*, 22(1), 2021.

[17] H. Anetzberger, R. Becker, H. Eickhoff, F.J. Seibert, B. Döring, F. Haasters, M. Mohr, and S. Reppenhagen. The diagnostic arthroscopy skill score (dass): a reliable and suitable assessment tool for arthroscopic skill training. *Knee Surgery, Sports Traumatology, Arthroscopy*, 30(1):349–360, 2022.

[18] P.P.J. Ang, B. Hugo, and R. Silvester. Acute postoperative pain management protocols in podiatric surgery within australia: a delphi study. *Journal of Foot and Ankle Research*, 15(1), 2022.

[19] P.D. Angevine, J.G. Zivin, and P.C. McCormick. Cost-effectiveness of single-level anterior cervical discectomy and fusion for cervical spondylosis. *Spine*, 30(17):1989–1997, 2005.

[20] J.K. Antonios, K.J. Bozic, H.D. Clarke, M.J. Spangehl, J.S. Bingham, and A.J. Schwartz. Cost-effectiveness of single vs double debridement and implant retention for acute periprosthetic joint infections in total knee arthroplasty: A markov model. *Arthroplasty Today*, 11:187–195, 2021.

[21] C. Bait, P. Randelli, R. Compagnoni, P. Ferrua, R. Papalia, F. Familiari, A. Tecame, P. Adravanti, E. Adriani, E. Arnaldi, F. Benazzo, M. Berruto, G. Bonaspetti, G.L. Canata, P.P. Canè, A. Causero, G. Coari, M. Denti, M. Farè, A. Ferretti, M. Fravisini, F. Giron, A. Gobbi, V. Madonna, A. Manunta, P.P. Mariani, C. Mazzola, G. Milano, L. Pederzini, F. Quaglia, M. Ronga, H. Schönhuber, G. Stefani, P. Volpi, G. Zanon, R. Zini, C. Zorzi, and S. Zaffagnini. Italian consensus statement for the use of allografts in acl reconstructive surgery. *Knee Surgery, Sports Traumatology, Arthroscopy*, 27(6):1873–1881, 2019.

[22] P. Barber and B.G. López-Valcárcel. Forecasting the need for medical specialists in spain: Application of a system dynamics model. *Human Resources for Health*, 8, 2010.

[23] C. Baril, V. Gascon, and S. Cartier. Design and analysis of an outpatient orthopaedic clinic performance with discrete event simulation and design of experiments. *Computers and Industrial Engineering*, 78:285–298, 2014.

[24] B.T. Barlow, A.S. McLawhorn, and G.H. Westrich. The cost-effectiveness of dual mobility implants for primary total hip arthroplasty: A computer-based cost-utility model. *Journal of Bone and Joint Surgery - American Volume*, 99(9):768–777, 2017.

[25] N.R. Barshes, J.D. Chambers, J. Cohen, M. Belkin, and Model To Optimize Healthcare Value in Ischemic Extremities 1 (MOVIE) Study Collaborators. Cost-effectiveness in the contemporary management of critical limb ischemia with tissue loss. *Journal of Vascular Surgery*, 56(4):1015–1024.e1, 2012.

[26] S. Bayona, K. Akhtar, C. Gupte, R.J.H. Emery, A.L. Dodds, and F. Bello. Assessing performance in shoulder arthroscopy: The imperial global arthroscopy rating scale (igars). *Journal of Bone and Joint Surgery - American Volume*, 96(13):e112(1), 2014.

[27] C. Becher, J. Beckmann, R. Von Eisenhart-Rothe, M. Hirschmann, J. Holz, A. Franz, C. Gwinner, R. Hube, and G. Matziolis. Unicondylar tibiofemoral arthroplasty - opinions of the members of the german knee society (dkg) and the german professional association of orthopaedic and trauma specialists (bvou). *Zeitschrift fur Orthopadie und Unfallchirurgie*, 159(1):47–53, 2021.

[28] H. Bedair, T.D. Cha, and V.J. Hansen. Economic benefit to society at large of total knee arthroplasty in younger patients a markov analysis. *Journal of Bone and Joint Surgery - Series A*, 96(2):119–126, 2014.

[29] H. Bedair, N. Ting, K.J. Bozic, C.J. Della Valle, and S.M. Sporer. Treatment of early postoperative infections after tha: A decision analysis. *Clinical Orthopaedics and Related Research*, 469(12):3477–3485, 2011.

[30] H. Bedair, J. Yang, M.K. Dwyer, and J.C. McCarthy. Preoperative erythropoietin alpha reduces postoperative transfusions in tha and tka but may not be cost-effective. *Clinical Orthopaedics and Related Research*, 473(2):590–596, 2015.

[31] M.S. Beerekamp, R. Haverlag, D.T. Ubbink, J.S. Luitse, K.J. Ponsen, and J.C. Goslings. How to evaluate the quality of fracture reduction and fixation of the wrist and ankle in clinical practice: A delphi consensus. *Archives of Orthopaedic and Trauma Surgery*, 131(6):739–746, 2011.

[32] M.S.H. Beerekamp, J.S.K. Luitse, D.T. Ubbink, M. Maas, N.W.L. Schep, and J.C. Goslings. Evaluation of reduction and fixation of calcaneal fractures: A delphi consensus. *Archives of Orthopaedic and Trauma Surgery*, 133(10):1377–1384, 2013.

[33] I. Bendich, W. Rubenstein, M. Mustafa Diab, and B. Feeley. Evaluating meniscus allograft transplant using a cost-effectiveness threshold analysis. *Knee*, 25(6):1171–1180, 2018.

[34] A.R. Berg, M.B. Held, B. Jiao, E. Swart, A. Lakra, H.J. Cooper, R.P. Shah, and J.A. Geller. Is the direct anterior approach to tha cost-effective? a markov analysis. *Clinical Orthopaedics and Related Research*, 480(8):1518–1532, 2022.

[35] S.B. Bhat, M. Lazarus, C. Getz, Jr. Williams, G.R., and S. Namdari. Economic decision model suggests total shoulder arthroplasty is superior to hemiarthroplasty in young patients with end-stage shoulder arthritis. *Clinical Orthopaedics and Related Research*, 474(11):2482–2492, 2016.

[36] S.B. Bhat, F.E. Liss, and P.K. Beredjiklian. Economic analysis of the cost of implants used for treatment of distal radius fractures. *Archives of Bone and Joint Surgery*, 6(5):371–375, 2018.

[37] M. Biddle, J.W. Kennedy, P.M. Wright, N.D. Ritchie, R.M.D. Meek, and B.P. Rooney. Improving outcomes in acute and chronic periprosthetic hip and knee joint infection with a multidisciplinary approach. *Bone and Joint Open*, 2(7):509–514, 2021.

[38] M. Bierbaum, O. Schöffski, B. Schliemann, and C. Kösters. Cost-utility analysis of dynamic intraligamentary stabilization versus early reconstruction after rupture of the anterior cruciate ligament. *Health Economics Review*, 7(1), 2017.

[39] S.A. Bini and J. Mahajan. Achieving 90% adoption of clinical practice guidelines using the delphi consensus method in a large orthopedic group. *Journal of Arthroplasty*, 31(11):2380–2384, 2016.

[40] J.A. Bishop, T.S. Crall, and M.S. Kocher. Operative versus nonoperative treatment after primary traumatic anterior glenohumeral dislocation: Expected-value decision analysis. *Journal of Shoulder and Elbow Surgery*, 20(7):1087–1094, 2011.

[41] R. Blythe, P.M. O'Gorman, R.W. Crawford, R. Feenan, A. Hatton, S.L. Whitehouse, and N. Graves. Fixation method for hip arthroplasty stem following hip fracture: A population-level cost-effectiveness analysis. *Journal of Arthroplasty*, 35(6):1614–1621, 2020.

[42] K.M.D. Bolz, R.W. Crawford, B. Donnelly, S.L. Whitehouse, and N. Graves. The cost-effectiveness of routine follow-up after primary total hip arthroplasty. *Journal of Arthroplasty*, 25(2):191–196, 2010.

[43] F. Borgström, Å. Carlsson, H. Sintonen, S. Boonen, P. Haentjens, R. Burge, O. Johnell, B. Jönsson, and J.A. Kanis. The cost-effectiveness of risedronate in the treatment of osteoporosis: An international perspective. *Osteoporosis International*, 17(7):996–1007, 2006.

[44] T.M. Borsinger, D.A. Pierce, T.M. Hanson, P.M. Werth, A.R. Orem, and W.E. Moschetti. Is the proportion of patients with "successful" outcomes after two-stage revision for prosthetic joint infection different when applying the musculoskeletal infection society outcome reporting tool compared with the delphi-based consensus criteria? *Clinical Orthopaedics and Related Research*, 479(7):1589–1597, 2021.

[45] A.M. Bove, K.J. Smith, C.G. Bise, J.M. Fritz, J. Childs, G.P. Brennan, J.H. Abbott, and G.K. Fitzgerald. Exercise, manual therapy, and booster sessions in knee osteoarthritis: Cost-effectiveness analysis from a multicenter randomized controlled trial. *Physical Therapy*, 98(1):16–27, 2018.

[46] J. Bowers. Waiting list behaviour and the consequences for nhs targets. *Journal of the Operational Research Society*, 61(2):246–254, 2010.

[47] J. Bowers and G. Mould. Managing uncertainty in orthopaedic trauma theatres. *European Journal of Operational Research*, 154(3):599–608, 2004.

[48] M.R. Boylan, III Bosco, J.A., and J.D. Slover. Cost-effectiveness of preoperative smoking cessation interventions in total joint arthroplasty. *Journal of Arthroplasty*, 34(2):215–220, 2019.

[49] K.J. Bozic, S. Morshed, M.D. Silverstein, H.E. Rubash, and J.G. Kahn. Use of cost-effectiveness analysis to evaluate new technologies in orthopaedics: The case of alternative bearing surfaces in total hip arthroplasty. *Journal of Bone and Joint Surgery - Series A*, 88(4):706–714, 2006.

[50] K.J. Bozic, C.M. Pui, M.J. Ludeman, T.P. Vail, and M.D. Silverstein. Do the potential benefits of metal-on-metal hip resurfacing justify the increased cost and

risk of complications? *Clinical Orthopaedics and Related Research*, 468(9):2301–2312, 2010.

[51] E. Braxton, B.J. Wohlfeld, S. Blumenthal, A. Bozzio, G. Buttermann, R. Guyer, J. Idema, D. Laich, J. Morreale, M. Nikolakis, A. Patel, J.S. Price, J.-P. Witt, J. Zigler, and M. Martin. Postoperative care pathways following lumbar total disc replacement: Results of a modified delphi approach. *Spine*, 44:S1–S12, 2019.

[52] A. Briggs, M. Sculpher, J. Dawson, R. Fitzpatrick, D. Murray, and H. Malchau. The use of probabilistic decision models in technology assessment: The case of total hip replacement. *Applied Health Economics and Health Policy*, 3(2):79–89, 2004.

[53] J. Brockbank and S. Wolowacz. Economic evaluations of new oral anticoagulants for the prevention of venous thromboembolism after total hip or knee replacement: A systematic review. *PharmacoEconomics*, 35(5):517–535, 2017.

[54] D.J. Brodke, S.K. Devana, A. Upfill-Brown, and C. Lee. Cost-effectiveness of fixation versus arthroplasty for geriatric distal femur fractures. *Injury*, 53(2):661–668, 2022.

[55] E. Brooks, M. Burns, R. Ma, H.J. Scholten, and S. Becker. Remote diabetic foot temperature monitoring for early detection of diabetic foot ulcers: A cost-effectiveness analysis. *ClinicoEconomics and Outcomes Research*, 13:873–881, 2021.

[56] E. Burn, A.D. Liddle, T.W. Hamilton, A. Judge, H.G. Pandit, D.W. Murray, and R. Pinedo-Villanueva. Cost-effectiveness of unicompartmental compared with total knee replacement: A population-based study using data from the national joint registry for england and wales. *BMJ Open*, 8(4), 2018.

[57] E. Burn, D.W. Murray, G.A. Hawker, R. Pinedo-Villanueva, and D. Prieto-Alhambra. Lifetime risk of knee and hip replacement following a gp diagnosis of osteoarthritis: a real-world cohort study. *Osteoarthritis and Cartilage*, 27(11):1627–1635, 2019.

[58] E. Burn, D. Prieto-Alhambra, T.W. Hamilton, J.A. Kennedy, D.W. Murray, and R. Pinedo-Villanueva. Threshold for computer- and robot-assisted knee and hip replacements in the english national health service. *Value in Health*, 23(6):719–726, 2020.

[59] J.W. Busse, M. Bhandari, S. Sprague, A.P. Johnson-Masotti, and A. Gafni. An economic analysis of management strategies for closed and open grade i tibial shaft fractures. *Acta Orthopaedica*, 76(5):705–712, 2005.

[60] S.C. Buttigieg, D. Gauci, F. Bezzina, and P.K. Dey. Post-surgery length of stay using multi-criteria decision-making tool. *Journal of Health Organization and Management*, 32(4):514–531, 2018.

[61] M. Bydon, M. MacKi, N.B. Abt, T.F. Witham, J.-P. Wolinsky, Z.L. Gokaslan, A. Bydon, and D.M. Sciubba. The cost-effectiveness of interbody fusions versus posterolateral fusions in 137 patients with lumbar spondylolisthesis. *Spine Journal*, 15(3):492–498, 2015.

[62] H. Campbell, J. Karnon, and R. Dowie. Cost analysis of a hospital-at-home initiative using discrete event simulation. *Journal of Health Services Research and Policy*, 6(1):14–22, 2001.

[63] K.J. Carnes, S.M. Odum, J.L. Troyer, and T.K. Fehring. Cost analysis of ceramic heads in primary total hip arthroplasty. *Journal of Bone and Joint Surgery - American Volume*, 98(21):1794–1800, 2016.

[64] L.Y. Carreon, S.D. Glassman, M. Djurasovic, M.J. Campbell, R.M. Puno, J.R. Johnson, and J.R. Dimar. Rhbmp-2 versus iliac crest bone graft for lumbar spine

fusion in patients over 60 years of age: A cost-utility study. *Spine*, 34(3):238–243, 2009.

[65] M.J. Carter. Dehydrated human amnion and chorion allograft versus standard of care alone in treatment of wagner 1 diabetic foot ulcers: a trial-based health economics study. *Journal of Medical Economics*, 23(11):1273–1283, 2020.

[66] A. Castagna, R. Garofalo, E. Maman, A.C. Gray, and E.A. Brooks. Comparative cost-effectiveness analysis of the subacromial spacer for irreparable and massive rotator cuff tears. *International Orthopaedics*, 43(2):395–403, 2019.

[67] C.M. Cavaliere and K.C. Chung. Total wrist arthroplasty and total wrist arthrodesis in rheumatoid arthritis: A decision analysis from the hand surgeons' perspective. *Journal of Hand Surgery*, 33(10):1744–1755.e2, 2008.

[68] C.M. Cavaliere and K.C. Chung. A cost-utility analysis of nonsurgical management, total wrist arthroplasty, and total wrist arthrodesis in rheumatoid arthritis. *Journal of Hand Surgery*, 35(3):379–391.e2, 2010.

[69] M. Ceballos, C.O. Valderrama, L.E. Orozco, L. Sánchez, J.P. Valderrama, and L.H. Lugo. Cost-utility analysis of reconstruction compared with primary amputation for patients with severe lower limb trauma in colombia. *Journal of Orthopaedic Trauma*, 31(9):e288–e294, 2017.

[70] G. Chang, S.B. Bhat, S.M. Raikin, J.M. Kane, A. Kay, J. Ahmad, D.I. Pedowitz, and J. Krieg. Economic analysis of anatomic plating versus tubular plating for the treatment of fibula fractures. *Orthopedics*, 41(2):e252–e256, 2018.

[71] R.W. Chang, J.M. Pellissier, and G.B. Hazen. A cost-effectiveness analysis of total hip arthroplasty for osteoarthritis of the hip. *JAMA*, 275(11):858–865, 1996.

[72] H. Chawla, B.U. Nwachukwu, J.P. Van Der List, A.A. Eggman, A.D. Pearle, and H.M. Ghomrawi. Cost effectiveness of patellofemoral versus total knee arthroplasty in younger patients. *Bone and Joint Journal*, 99B(8):1028–1036, 2017.

[73] C. Chen, M. Hanson, R. Chaturvedi, S. Mattke, R. Hillestad, and H.H. Liu. Economic benefits of microprocessor controlled prosthetic knees: A modeling study. *Journal of NeuroEngineering and Rehabilitation*, 15, 2018.

[74] L. Chokotho, C.A. Donnelley, S. Young, B.C. Lau, H.-H. Wu, N. Mkandawire, J.-E. Gjertsen, G. Hallan, K.J. Agarwal-Harding, and D. Shearer. Cost utility analysis of intramedullary nailing and skeletal traction treatment for patients with femoral shaft fractures in malawi. *Acta Orthopaedica*, 92(4):436–442, 2021.

[75] I. Chow, E.V. Lemos, and T.R. Einarson. Management and prevention of diabetic foot ulcers and infections: A health economic review. *PharmacoEconomics*, 26(12):1019–1035, 2008.

[76] A.B. Christian, P.T. Delaplain, A. Grigorian, J. Nahmias, L. Mueller, E. Tay, W.Q. Duong, W.Y. Rockne, and S.D. Schubl. Research priorities in chest wall injury: A modified delphi approach. *Journal of Trauma and Acute Care Surgery*, 89(4):e106–e111, 2020.

[77] S.M. Christopher, A.N. Garcia, S.J. Snodgrass, and C. Cook. Common musculoskeletal impairments in postpartum runners: an international delphi study. *Archives of Physiotherapy*, 10(1), 2020.

[78] A.W. Chuck, D. Hailey, P. Jacobs, and D.C. Perry. Cost-effectiveness and budget impact of adjunctive hyperbaric oxygen therapy for diabetic foot ulcers. *International Journal of Technology Assessment in Health Care*, 24(2):178–183, 2008.

[79] K.C. Chung, M.J. Shauver, D. Saddawi-Konefka, and S.C. Haase. A decision analysis of amputation versus reconstruction for severe open tibial fracture from

the physician and patient perspectives. *Annals of Plastic Surgery*, 66(2):185–191, 2011.

[80] L.E. Cipriano, B.M. Chesworth, C.K. Anderson, and G.S. Zaric. Predicting joint replacement waiting times. *Health Care Management Science*, 10(2):195–215, 2007.

[81] L.E. Cipriano, B.M. Chesworth, C.K. Anderson, and G.S. Zaric. An evaluation of strategies to reduce waiting times for total joint replacement in ontario. *Medical Care*, 46(11):1177–1183, 2008.

[82] N.D. Clement, D.J. Deehan, and J.T. Patton. Robot-assisted unicompartmental knee arthroplasty for patients with isolated medial compartment osteoarthritis is cost-effective: A markov decision analysis. *Bone and Joint Journal*, 101-B(9):1063–1070, 2019.

[83] R.C. Clement, P.J. Lang, B.J. Pettett, R.A. Overman, R.F. Ostrum, and J.N. Tennant. Sanders ii/iii calcaneus fractures in laborers: A cost-effectiveness analysis and call for effectiveness research. *Journal of Orthopaedic Trauma*, 31(6):299–304, 2017.

[84] M.P. Coe, R.M. Greiwe, R. Joshi, B.M. Snyder, L. Simpson, A.N.A. Tosteson, C.S. Ahmad, W.N. Levine, and J.-E. Bell. The cost-effectiveness of reverse total shoulder arthroplasty compared with hemiarthroplasty for rotator cuff tear arthropathy. *Journal of Shoulder and Elbow Surgery*, 21(10):1278–1288, 2012.

[85] C.W. Colwell, M.I. Froimson, M.A. Mont, M.A. Ritter, R.T. Trousdale, K.C. Buehler, A.I. Spitzer, T.K. Donaldson, and D.E. Padgett. Cost-effectiveness of venous thromboembolism prophylaxis with a new mobile device after total hip arthroplasty. *Journal of Arthroplasty*, 27(8):1513–1517.e1, 2012.

[86] T. Comans, M. Raymer, S. O'Leary, D. Smith, and P. Scuffham. Cost-effectiveness of a physiotherapist-led service for orthopaedic outpatients. *Journal of Health Services Research and Policy*, 19(4):216–223, 2014.

[87] M. Comas, R. Román, J.M. Quintana, and X. Castells. Unmet needs and waiting list prioritization for knee arthroplasty. *Clinical Orthopaedics and Related Research*, 468(3):789–797, 2010.

[88] C. Cook, J.-M. Brismée, R. Fleming, and P.S. Sizer Jr. Identifiers suggestive of clinical cervical spine instability: A delphi study of physical therapists. *Physical Therapy*, 85(9):895–906, 2005.

[89] X.F. Courville, P.J. Hecht, and A.N.A. Tosteson. Is total ankle arthroplasty a cost-effective alternative to ankle fusion? *Clinical Orthopaedics and Related Research*, 469(6):1721–1727, 2011.

[90] X.F. Courville, I.M. Tomek, K.B. Kirkland, M. Birhle, S.R. Kantor, and S.R.G. Finlayson. Cost-effectiveness of preoperative nasal mupirocin treatment in preventing surgical site infection in patients undergoing total hip and knee arthroplasty: A cost-effectiveness analysis. *Infection Control and Hospital Epidemiology*, 33(2):152–159, 2012.

[91] D. Coyle, K.M. Lee, D.A. Fergusson, and A. Laupacis. Economic analysis of erythropoietin use in orthopaedic surgery. *Transfusion Medicine*, 9(1):21–30, 1999.

[92] S. Coyle, S. Kinsella, B. Lenehan, and J.M. Queally. Cost-utility analysis in orthopaedic trauma; what pays? a systematic review. *Injury*, 49(3):575–584, 2018.

[93] T.S. Crall, J.A. Bishop, D. Guttman, M. Kocher, K. Bozic, and J.H. Lubowitz. Cost-effectiveness analysis of primary arthroscopic stabilization versus nonoperative treatment for first-time anterior glenohumeral dislocations. *Arthroscopy -*

Journal of Arthroscopic and Related Surgery, 28(12):1755–1765, 2012.

[94] W.M. Cregar, A. Beletsky, G.L. Cvetanovich, B.T. Feeley, G.P. Nicholson, and N.N. Verma. Cost-effectiveness analyses in shoulder arthroplasty: a critical review using the quality of health economic studies (qhes) instrument. *Journal of Shoulder and Elbow Surgery*, 30(5):1007–1017, 2021.

[95] D. Cumming, F. Song, R.S. Taylor, M. Zahra, A. Williams, and S. Eggington. Cost-effectiveness of 4 mg dibotermin alfa/absorbable collagen sponge versus iliac crest bone graft for lumbar degenerative disc disease in the united kingdom. *Journal of Medical Economics*, 25(1):59–65, 2022.

[96] J.S. Cummins, I.M. Tomek, S.R. Kantor, O. Furnes, L.B. Engesæter, and S.R.G. Finlayson. Cost-effectiveness of antibiotic-impregnated bone cement used in primary total hip arthroplasty. *Journal of Bone and Joint Surgery - Series A*, 91(3):634–641, 2009.

[97] M.K. Cárdenas, A.J. Mirelman, C.J. Galvin, M. Lazo-Porras, M. Pinto, J.J. Miranda, and R.H. Gilman. The cost of illness attributable to diabetic foot and cost-effectiveness of secondary prevention in peru. *BMC Health Services Research*, 15(1), 2015.

[98] J. Dahmen, S. Bayer, J. Toale, C. Mulvin, E.T. Hurley, J. Batista, G.C. Berlet, C.W. DiGiovanni, R.D. Ferkel, Y. Hua, S. Kearns, J.W. Lee, C.J. Pearce, H. Pereira, M.P. Prado, S.M. Raikin, L.C. Schon, J.W. Stone, M. Sullivan, M. Takao, V. Valderrabano, C.N. van Dijk, Z. Ali, J.N. Altink, R. Buda, J.D.F. Calder, M.S. Davey, P. D'Hooghe, A.L. Gianakos, E. Giza, M. Glazebrook, L. Hangody, D. Haverkamp, B. Hintermann, M.V. Hogan, K.J. Hunt, D.J. Hurley, M.S. Jamal, J. Karlsson, J.G. Kennedy, G.M.M.J. Kerkhoffs, K.T.A. Lambers, G. McCollum, N.P. Mercer, II Nunley, J.A., J. Paul, I. Savage-Elliott, Y. Shimozono, S.A.S. Stufkens, H. Thermann, D. Thordarson, F. Vannini, C.J.A. van Bergen, R.J. Walls, M. Walther, Y. Yasui, A.S.E. Younger, and C.D. Murawski. Osteochondral lesions of the tibial plafond and ankle instability with ankle cartilage lesions: Proceedings of the international consensus meeting on cartilage repair of the ankle. *Foot and Ankle International*, 43(3):448–452, 2022.

[99] H. Dakin, P. Eibich, D. Beard, A. Gray, and A. Price. The use of patient-reported outcome measures to guide referral for hip and knee arthroplasty: Part 2: A cost-effectiveness analysis. *Bone and Joint Journal*, 102-B(7):950–958, 2020.

[100] E.N. Davis, K.C. Chung, S.V. Kotsis, F.H. Lau, and S. Vijan. A cost/utility analysis of open reduction and internal fixation versus cast immobilization for acute nondisplaced mid-waist scaphoid fractures. *Plastic and Reconstructive Surgery*, 117(4):1223–1235, 2006.

[101] D.M. Dawoud, D. Wonderling, J. Glen, S. Lewis, X. Griffin, B.J. Hunt, G. Stansby, M. Reed, N. Rossiter, J.K. Chahal, C. Sharpin, and P. Barry. Cost-utility analysis of venous thromboembolism prophylaxis strategies for people undergoing elective total hip and total knee replacement surgeries in the english national health service. *Frontiers in Pharmacology*, 9(NOV), 2018.

[102] P. Dayton, J.G. DeVries, A. Landsman, A. Meyr, and M. Schweinberger. American college of foot and ankle surgeons' clinical consensus statement: Perioperative prophylactic antibiotic use in clean elective foot surgery. *Journal of Foot and Ankle Surgery*, 54(2):273–279, 2015.

[103] A.F.S. de Brito, N.C. Brito, S.K.T. Tanaka, V.L. Ferreira, A.B.S. Ferreira Júnior, B.S. Riveros, and M.E. Nita. Thromboprophylaxis of patients submitted to total hip and knee arthroplasty: A cost-effectiveness assessment from the perspective of the brazilian national health system. *Value in Health Regional Issues*, 31:111–

118, 2022.

[104] C.J. DeFrancesco, D.G. Lebrun, Jr. Molony, J.T., M.R. Heath, and P.D. Fabricant. Safer and cheaper: An enhanced milestone-based return to play program after anterior cruciate ligament reconstruction in young athletes is cost-effective compared with standard time-based return to play criteria. *American Journal of Sports Medicine*, 48(5):1100–1107, 2020.

[105] C. Dehlendorff, M. Kulahci, and K.K. Andersen. Analysis of computer experiments with multiple noise sources. *Quality and Reliability Engineering International*, 26(2):137–146, 2010.

[106] G.L. Di Tanna, S. Ferro, F. Cipriani, B. Bordini, S. Stea, A. Toni, F. Silipo, G. Pirini, and R. Grilli. Modeling the cost-effectiveness for cement-less and hybrid prosthesis in total hip replacement in emilia romagna, italy. *Journal of Surgical Research*, 169(2):227–233, 2011.

[107] A. Diamantopoulos, M. Lees, P.S. Wells, F. Forster, J. Ananthapavan, and H. McDonald. Cost-effectiveness of rivaroxaban versus enoxaparin for the prevention of postsurgical venous thromboembolism in canada. *Thrombosis and Haemostasis*, 104(4):760–770, 2010.

[108] C. Diaz-Ledezma, C.A. Higuera, and J. Parvizi. Success after treatment of periprosthetic joint infection: A delphi-based international multidisciplinary consensus infection. *Clinical Orthopaedics and Related Research*, 471(7):2374–2382, 2013.

[109] C. Diaz-Ledezma, P.M. Lichstein, J.G. Dolan, and J. Parvizi. Diagnosis of periprosthetic joint infection in medicare patients: Multicriteria decision analysis. *Clinical Orthopaedics and Related Research*, 472(11):3275–3284, 2014.

[110] C. Diaz-Ledezma and J. Parvizi. Surgical approaches for cam femoroacetabular impingement: The use of multicriteria decision analysis hip. *Clinical Orthopaedics and Related Research*, 471(8):2509–2516, 2013.

[111] T. Diermeier, B.B. Rothrauff, L. Engebretsen, A.D. Lynch, O.R. Ayeni, M.V. Paterno, J.W. Xerogeanes, F.H. Fu, J. Karlsson, V. Musahl, E. Svantesson, E. Hamrin Senorski, T. Rauer, S.J. Meredith, and The Panther Symposium ACL Treatment Consensus Group. Treatment after anterior cruciate ligament injury: Panther symposium acl treatment consensus group. *Knee Surgery, Sports Traumatology, Arthroscopy*, 28(8):2390–2402, 2020.

[112] H. Dong and M. Buxton. Early assessment of the likely cost-effectiveness of a new technology: A markov model with probabilistic sensitivity analysis of computer-assisted total knee replacement. *International Journal of Technology Assessment in Health Care*, 22(2):191–202, 2006.

[113] H. Dong, D. Coyle, and M. Buxton. Value of information analysis for a new technology: Computer-assisted total knee replacement. *International Journal of Technology Assessment in Health Care*, 23(3):337–342, 2007.

[114] G.J. Dornan, J.C. Katthagen, D.S. Tahal, M. Petri, J.A. Greenspoon, P.J. Denard, S.S. Burkhart, and P.J. Millett. Cost-effectiveness of arthroscopic rotator cuff repair versus reverse total shoulder arthroplasty for the treatment of massive rotator cuff tears in patients with pseudoparalysis and nonarthritic shoulders. *Arthroscopy - Journal of Arthroscopic and Related Surgery*, 33(4):716–725, 2017.

[115] G. Dranitsaris, C. Stumpo, R. Smith, and W. Bartle. Extended dalteparin prophylaxis for venous thromboembolic events: Cost-utility analysis in patients undergoing major orthopedic surgery. *American Journal of Cardiovascular Drugs*, 9(1):45–58, 2009.

[116] M. Drummond, M. Aristides, L. Davies, and C. Forbes. Economic evaluation of standard heparin and enoxaparin for prophylaxis against deep vein thrombosis in elective hip surgery. *British Journal of Surgery*, 81(12):1742–1746, 1994.

[117] A. Duran, N. Sengupta, A. Diamantopoulos, F. Forster, L. Kwong, and M. Lees. Cost effectiveness of rivaroxaban versus enoxaparin for prevention of post-surgical venous thromboembolism from a us payer's perspective. *PharmacoEconomics*, 30(2):87–101, 2012.

[118] A. Durojaiye, J. Fackler, N. McGeorge, K. Webster, H. Kharrazi, and A. Gurses. Examining diurnal differences in multidisciplinary care teams at a pediatric trauma center using electronic health record data: Social network analysis. *Journal of Medical Internet Research*, 24(2), 2022.

[119] A. Ebadi, P.J. Tighe, L. Zhang, and P. Rashidi. Disteam: A decision support tool for surgical team selection. *Artificial Intelligence in Medicine*, 76:16–26, 2017.

[120] M.H. Eckman, J.B. Wong, S.G. Pauker, S. Greenfield, K. Dukes, S. Kaplan, L. Sullivan, and W.C. Mackey. Foot infections in diabetic patients: Decision and cost-effectiveness analyses. *JAMA: The Journal of the American Medical Association*, 273(9):712–720, 1995.

[121] D.S. Edwards, R.D. Phillip, N. Bosanquet, A.M.J. Bull, and J.C. Clasper. What is the magnitude and long-term economic cost of care of the british military afghanistan amputee cohort? *Clinical Orthopaedics and Related Research*, 473(9):2848–2855, 2015.

[122] A.M. Elbuluk, J. Slover, A.A. Anoushiravani, R. Schwarzkopf, N. Eftekhary, and J.M. Vigdorchik. The cost-effectiveness of dual mobility in a spinal deformity population with high risk of dislocation: A computer-based model. *Bone and Joint Journal*, 100B(10):1297–1302, 2018.

[123] J.-A. Epinette, A. Lafuma, J. Robert, and M. Doz. Cost-effectiveness model comparing dual-mobility to fixed-bearing designs for total hip replacement in france. *Orthopaedics and Traumatology: Surgery and Research*, 102(2):143–148, 2016.

[124] J.S. Everhart, A.B. Campbell, M.M. Abouljoud, J.C. Kirven, and D.C. Flanigan. Cost-efficacy of knee cartilage defect treatments in the united states. *American Journal of Sports Medicine*, 48(1):242–251, 2020.

[125] M. Farshad, C. Gerber, D.C. Meyer, A. Schwab, P.R. Blank, and T. Szucs. Reconstruction versus conservative treatment after rupture of the anterior cruciate ligament: Cost effectiveness analysis. *BMC Health Services Research*, 11, 2011.

[126] S.C. Faucett, C.A. Collinge, and K.J. Koval. Is reconstruction nailing of all femoral shaft fractures cost effective? a decision analysis. *Journal of Orthopaedic Trauma*, 26(11):624–632, 2012.

[127] S.C. Faucett, B.P. Geisler, J. Chahla, A.J. Krych, P.R. Kurzweil, A.M. Garner, S. Liu, R.F. LaPrade, and J.B. Pietzsch. Meniscus root repair vs meniscectomy or nonoperative management to prevent knee osteoarthritis after medial meniscus root tears: Clinical and economic effectiveness. *American Journal of Sports Medicine*, 47(3):762–769, 2019.

[128] S.C. Faucett, J.W. Genuario, A.N.A. Tosteson, and K.J. Koval. Is prophylactic fixation a cost-effective method to prevent a future contralateral fragility hip fracture? *Journal of Orthopaedic Trauma*, 24(2):65–74, 2010.

[129] C.G. Fawsitt, H.H.Z. Thom, L.P. Hunt, S. Nemes, A.W. Blom, N.J. Welton, W. Hollingworth, J.A. López-López, A.D. Beswick, A. Burston, O. Rolfson, G. Garellick, and E.M.R. Marques. Choice of prosthetic implant combinations

in total hip replacement: Cost-effectiveness analysis using uk and swedish hip joint registries data. *Value in Health*, 22(3):303–312, 2019.

[130] A.E. Federer, III Mather, R.C., M.L. Ramsey, and G.E. Garrigues. Cost-effectiveness analysis of total elbow arthroplasty versus open reduction–internal fixation for distal humeral fractures. *Journal of Shoulder and Elbow Surgery*, 28(1):102–111, 2019.

[131] B.T. Feeley, S. Liu, A.M. Garner, A.L. Zhang, and J.B. Pietzsch. The cost-effectiveness of meniscal repair versus partial meniscectomy: A model-based projection for the united states. *Knee*, 23(4):674–680, 2016.

[132] P. Fennema, T.J. Heyse, and C.A. Uyl-De Groot. Cost-effectiveness and clinical implications of advanced bearings in total knee arthroplasty: A long-term modeling analysis. *International Journal of Technology Assessment in Health Care*, 30(2):218–225, 2014.

[133] J.-L. Fernandez, A. McGuire, and M. Raikou. Hospital coordination and integration with social care in england: The effect on post-operative length of stay. *Journal of Health Economics*, 61:233–243, 2018.

[134] L. Figueiredo, E.C. Makhni, M. Dierks, F.C. Ferreira, and S. Finkelstein. Early cost estimating model for new bioabsorbable orthopedic implant candidates: A theoretical study. *Journal of the Mechanical Behavior of Biomedical Materials*, 124, 2021.

[135] D.N. Fisman, D.T. Reilly, A.W. Karchmer, and S.J. Goldie. Clinical effectiveness and cost-effectiveness of 2 management strategies for infected total hip arthroplasty in the elderly. *Clinical Infectious Diseases*, 32(3):419–430, 2001.

[136] H.M. Fox, L.J. Hsue, A.R. Thompson, D.C. Ramsey, R.W. Hadden, A.J. Mirarchi, and O.F. Nazir. Humeral shaft fractures: a cost-effectiveness analysis of operative versus nonoperative management. *Journal of Shoulder and Elbow Surgery*, 31(9):1969–1981, 2022.

[137] H.M. Fox, D.C. Ramsey, A.R. Thompson, C.J. Hoekstra, A.J. Mirarchi, and O.F. Nazir. Neer type-ii distal clavicle fractures: A cost-effectiveness analysis of fixation techniques. *Journal of Bone and Joint Surgery*, 102(3):254–261, 2020.

[138] T. Francis, T. Washington, K. Srivastava, V. Moutzouros, E.C. Makhni, and W. Hakeos. Societal costs in displaced transverse olecranon fractures: using decision analysis tools to find the most cost-effective strategy between tension band wiring and locked plating. *Journal of Shoulder and Elbow Surgery*, 26(11):1995–2003, 2017.

[139] S. Franovic, A. Pietroski, N. Kuhlmann, T. Bazzi, Y. Zhou, and S. Muh. Rockwood grade-iii acromioclavicular joint separation a cost-effectiveness analysis of treatment options. *JBJS Open Access*, 6(2), 2021.

[140] J. Frappier, W. Stanish, M. Brittberg, M. Steinwachs, L. Crowe, D. Castelo, and A. Restrepo. Economic evaluation of bst-cargel as an adjunct to microfracture vs microfracture alone in knee cartilage surgery. *Journal of Medical Economics*, 17(4):266–278, 2014.

[141] R.J. Friedman and G.A. Dunsworth. Cost analyses of extended prophylaxis with enoxaparin after hip arthroplasty. *Clinical Orthopaedics and Related Research*, 370:171–182, 2000.

[142] F. Frosini, R. Miniati, P. Avezzano, F. Dori, D. Cocchi, E. Iadanza, S. Belli, M.T. Mechi, V. Ceccherini, and A. Belardinelli. A simulation based model for planning operating theater activity in complex hospitals: Case study in orthopedics. volume 51, pages 1554–1557. Springer Verlag, 2015.

[143] A.R. Fréz, S.R. Alouche, A.C. Binda, and C.M. Nunes Cabral. Content validity

of the international classification of functioning, disability and health core set for knee dysfunction: a delphi study. *Physiotherapy Theory and Practice*, 2022.

[144] C.C. Fuller, C. Kweon, T. Baker, C. Reese, and W.D. Lack. Cost-effectiveness analysis of short versus long cephalomedullary nails for treatment of stable intertrochanteric femoral fractures: A theoretical cohort study. *Current Orthopaedic Practice*, 32(6):584–590, 2021.

[145] S.S. Gagnon, T.B. Birmingham, B.M. Chesworth, D. Bryant, M. Werstine, and J.R. Giffin. Development of a clinician-rated drop vertical jump scale for patients undergoing rehabilitation after anterior cruciate ligament reconstruction: A delphi approach. *Journal of Orthopaedic and Sports Physical Therapy*, 47(8):557–564, 2017.

[146] S.M. Gancarczyk, E.S. Jang, E.P. Swart, E.C. Makhni, and R.K. Kadiyala. Percutaneous trigger finger release: A cost-effectiveness analysis. *Journal of the American Academy of Orthopaedic Surgeons*, 24(7):475–482, 2016.

[147] A. Gandjour and E.-J. Weyler. Cost-effectiveness of referrals to high-volume hospitals: An analysis based on a probabilistic markov model for hip fracture surgeries. *Health Care Management Science*, 9(4):359–369, 2006.

[148] K. Geale, M. Álvarez, M. Polyzoi, X. Màlaga, C. Pineda, and C. Hernández. Budget impact analysis of demineralized bone matrix in combination with autograft in lumbar spinal fusion procedures for the treatment of lumbar degenerative disc disease in spain. *Journal of Medical Economics*, 21(10):977–982, 2018.

[149] J.W. Genuario, R.P. Donegan, D. Hamman, J.-E. Bell, M. Boublik, T. Schlegel, and A.N.A. Tosteson. The cost-effectiveness of single-row compared with double-row arthroscopic rotator cuff repair. *Journal of Bone and Joint Surgery - Series A*, 94(15):1369–1377, 2012.

[150] J.W. Genuario, S.C. Faucett, M. Boublik, and T.F. Schlegel. A cost-effectiveness analysis comparing 3 anterior cruciate ligament graft types: Bone-patellar tendon-bone autograft, hamstring autograft, and allograft. *American Journal of Sports Medicine*, 40(2):307–314, 2012.

[151] J. George, D. Gautam, N. Devasenapathy, and R. Malhotra. Is it worth delaying total knee replacement as late as possible? a cost-effectiveness analysis using a markov model in the indian setting. *Value in Health Regional Issues*, 24:173–180, 2021.

[152] L. Gerlier, M. Lamotte, M. Wille, P.C. Kreuz, J. Vanlauwe, D. Dubois, and F.M. Meurgey. The cost utility of autologous chondrocytes implantation using chondrocelect® in symptomatic knee cartilage lesions in belgium. *PharmacoEconomics*, 28(12):1129–1146, 2010.

[153] H.M. Ghomrawi, A.A. Eggman, and A.D. Pearle. Effect of age on cost-effectiveness of unicompartmental knee arthroplasty compared with total knee arthroplasty in the u.s. *Journal of Bone and Joint Surgery - American Volume*, 97(5):396–402, 2015.

[154] J. Gillespie, S. McClean, F. FitzGibbons, B. Scotney, F. Dobbs, and B.J. Meenan. Do we need stochastic models for healthcare? the case of icats? *Journal of Simulation*, 8(4):293–303, 2014.

[155] A.M. Gilligan, C.R. Waycaster, and T.A. Motley. Cost-effectiveness of becaplermin gel on wound healing of diabetic foot ulcers. *Wound Repair and Regeneration*, 23(3):353–360, 2015.

[156] T.D. Goldberg, J.A. Maltry, M. Ahuja, and J.A. Inzana. Logistical and economic advantages of sterile-packed, single-use instruments for total knee arthroplasty. *Journal of Arthroplasty*, 34(9):1876–1883.e2, 2019.

[157] C.A. Gottlob, C.L. Baker Jr., J.M. Pellissier, and L. Colvin. Cost effectiveness of anterior cruciate ligament reconstruction in young adults. *Clinical Orthopaedics and Related Research*, 367:272–282, 1999.

[158] A.E. Graham, S.Q. Xie, K.C. Aw, S. Mukherjee, and W.L. Xu. Bone-muscle interaction of the fractured femur. *Journal of Orthopaedic Research*, 26(8):1159–1165, 2008.

[159] N. Graves, C. Wloch, J. Wilson, A. Barnett, A. Sutton, N. Cooper, K. Merollini, V. McCreanor, Q. Cheng, E. Burn, T. Lamagni, and A. Charlett. A cost-effectiveness modelling study of strategies to reduce risk of infection following primary hip replacement based on a systematic review. *Health Technology Assessment*, 20(54):1–144, 2016.

[160] Q. Gu, L. Koenig, III Mather, R.C., and J. Tongue. Surgery for hip fracture yields societal benefits that exceed the direct medical costs. *Clinical Orthopaedics and Related Research*, 472(11):3536–3546, 2014.

[161] R.E. Guerrero-Ludueña, M. Comas, M. Espallargues, M. Coll, M. Pons, S. Sabatés, A. Allepuz, and X. Castells. Predicting the burden of revision knee arthroplasty: Simulation of a 20-year horizon. *Value in Health*, 19(5):680–687, 2016.

[162] J.F. Guest, L. Atkin, and C. Aitkins. Potential cost-effectiveness of using adjunctive dehydrated human amnion/chorion membrane allograft in the management of non-healing diabetic foot ulcers in the united kingdom. *International Wound Journal*, 18(6):889–901, 2021.

[163] A.-L. Guillermin, A. Lloyd, J.H. Best, M.B. Deyoung, Y. Samyshkin, and J.A. Gaebler. Long-term cost-consequence analysis of exenatide once weekly vs sitagliptin or pioglitazone for the treatment of type 2 diabetes patients in the united states. *Journal of Medical Economics*, 15(4):654–663, 2012.

[164] A.-L. Guillermin, Y. Samyshkin, D. Wright, T. Nguyen, and J. Villeneuve. Modeling the lifetime costs of insulin glargine and insulin detemir in type 1 and type 2 diabetes patients in canada: A meta-analysis and a cost-minimization analysis. *Journal of Medical Economics*, 14(2):207–216, 2011.

[165] A. Gustafsson, B. Viberg, C. Paltved, H. Palm, L. Konge, and L.J. Nayahangan. Identifying technical procedures in orthopaedic surgery and traumatology that should be integrated in a simulation-based curriculum: A national general needs assessment in denmark. *Journal of Bone and Joint Surgery - American Volume*, 101(20):E108, 2019.

[166] S. Gyftopoulos, J. Conroy, J. Koo, M. Jones, A. Miniaci, and N. Subhas. Imaging of patients suspected of slap tear: A cost-effectiveness study. *American Journal of Roentgenology*, 218(2):227–233, 2022.

[167] S. Gyftopoulos, K.E. Guja, N. Subhas, M.S. Virk, and H.T. Gold. Cost-effectiveness of magnetic resonance imaging versus ultrasound for the detection of symptomatic full-thickness supraspinatus tendon tears. *Journal of Shoulder and Elbow Surgery*, 26(12):2067–2077, 2017.

[168] J.F. Gómez-Cerezo, I. Gómez-Arrayás, C. Suárez-Fernández, L. Betegón-Nicolás, M. de Salas-Cansado, and C. Rubio-Terrés. Cost-effectiveness analysis of apixaban compared to dabigatran in the prevention of venous thromboembolism in patients subjected to total knee or hip replacement [análisis coste-efectividad de apixaban frente a dabigatrán en la prevención de la tromboembolia venosa en pacientes intervenidos de artroplastia total de rodilla o de cadera]. *Revista Espanola de Cirugia Ortopedica y Traumatologia*, 56(6):459–470, 2012.

[169] A. Gómez-Outes, C. Avendaño-Solá, A.I. Terleira-Fernández, and E. Vargas-

Castrillón. Pharmacoeconomic evaluation of dabigatran, rivaroxaban and apixaban versus enoxaparin for the prevention of venous thromboembolism after total hip or knee replacement in spain. *PharmacoEconomics*, 32(9):919–936, 2014.

[170] O. Gøthesen, J. Slover, L. Havelin, J.E. Askildsen, H. Malchau, and O. Furnes. An economic model to evaluate cost-effectiveness of computer assisted knee replacement surgery in norway. *BMC Musculoskeletal Disorders*, 14, 2013.

[171] W. Habacher, I. Rakovac, E. Görzer, W. Haas, R.J. Gfrerer, P. Wach, and T.R. Pieber. A model to analyse costs and benefit of intensified diabetic foot care in austria. *Journal of Evaluation in Clinical Practice*, 13(6):906–912, 2007.

[172] P. Haentjens, K. De Groote, and L. Annemans. Prolonged enoxaparin therapy to prevent venous thromboembolism after primary hip or knee replacement. a cost-utility analysis. *Archives of Orthopaedic and Trauma Surgery*, 124(8):507–517, 2004.

[173] V. Hamidi, T. Ringerike, G. Hagen, A. Reikvam, and M. Klemp. New anticoagulants as thromboprophylaxis after total hip or knee replacement. *International Journal of Technology Assessment in Health Care*, 29(3):234–243, 2013.

[174] C.P. Hannon, S. Bayer, C.D. Murawski, G.L. Canata, T.O. Clanton, D. Haverkamp, J.W. Lee, M.J. O'Malley, H. Yinghui, J.W. Stone, J. Ackermann, Jr. Adams, S.B., C.L. Andrews, C. Angthong, J.P. Batista, O.L. Baur, C. Becher, G.C. Berlet, L.A.T. Boakye, A.J. Brown, R. Buda, J.D.F. Calder, D.S. Carreira, J. Dahmen, P. D'Hooghe, C.W. DiGiovanni, M.E. Dombrowski, M.C. Drakos, R.D. Ferkel, P.N.F. Ferrao, L.A. Fortier, M. Glazebrook, E. Giza, M. Gomaa, S. Görtz, A.M. Haleem, K. Hamid, L. Hangody, J. Hertel, B. Hintermann, M.V. Hogan, K.J. Hunt, E.T. Hurley, J. Karlsson, S.R. Kearns, J.G. Kennedy, G.M.M.J. Kerkhoffs, H.J. Kim, S.W. Kong, S.A. Labib, K.T.A. Lambers, K. Bae Lee, J.S. Ling, U. Giuseppe Longo, A. Marangon, G. McCollum, A.W. Mitchell, P.N. Mittwede, S. Nehrer, P. Niemeyer, II Nunley, J.A., D.O. Osei-Hwedieh, J. Paul, C.J. Pearce, H. Pereira, A. Popchak, M.P. Prado, S.M. Raikin, M.L. Reilingh, B.B. Rothrauff, L.C. Schon, Y. Shimozono, H. Simpson, N.A. Smyth, C.M. Sofka, P. Spennacchio, M. Sullivan, M. Takao, Y. Tanaka, D.B. Thordarson, R. Tuan, V. Valderrabano, C.J.A. van Bergen, C.N. van Dijk, P.A.D. van Dijk, F. Vannini, T. Vaseenon, M. Walther, M. Wiewiorski, X. Xu, Y. Yasui, I. Yoshimura, A.S.E. Younger, Z. Zhang, and the International Consensus Group on Cartilage Repair of the Ankle. Debridement, curettage, and bone marrow stimulation: Proceedings of the international consensus meeting on cartilage repair of the ankle. *Foot and Ankle International*, 39(1_suppl):16S–22S, 2018.

[175] E. Hansson, K. Hagberg, M. Cawson, and T.H. Brodtkorb. Patients with unilateral transfemoral amputation treated with a percutaneous osseointegrated prosthesis: A cost-effectiveness analysis. *Bone and Joint Journal*, 100B(4):527–534, 2018.

[176] M. Hauser, R. King, R. Wysk, and O. Harrysson. Resource planning for direct fabrication of customized orthopedic implants using ebm technology. *Journal of Manufacturing Systems*, 60:500–511, 2021.

[177] S. Heintzbergen, N.A. Kulin, M.J. Ijzerman, L.M.G. Steuten, J. Werle, H. Khong, and D.A. Marshall. Cost-utility of metal-on-metal hip resurfacing compared to conventional total hip replacement in young active patients with osteoarthritis. *Value in Health*, 16(6):942–952, 2013.

[178] N. Helmy, C. Anglin, N.V. Greidanus, and B.A. Masri. To resurface or not

to resurface the patella in total knee arthroplasty. *Clinical Orthopaedics and Related Research*, 466(11):2775–2783, 2008.

[179] R.J. Hernandez, R.G. Cornell, and R.N. Hensinger. Ultrasound diagnosis of neonatal congenital dislocation of the hip. a decision analysis assessment. *Journal of Bone and Joint Surgery - Series B*, 76(4):539–543, 1994.

[180] H. Higashi and J.J. Barendregt. Cost-effectiveness of total hip and knee replacements for the australian population with osteoarthritis: Discrete-event simulation model. *PLoS ONE*, 6(9), 2011.

[181] M. Hiligsmann, W. Ben Sedrine, O. Bruyère, and J.-Y. Reginster. Cost-effectiveness of strontium ranelate in the treatment of male osteoporosis. *Osteoporosis International*, 24(8):2291–2300, 2013.

[182] M. Hiligsmann, W.B. Sedrine, and J.-Y. Reginster. Cost-effectiveness of bazedoxifene compared with raloxifene in the treatment of postmenopausal osteoporotic women. *Journal of Bone and Mineral Research*, 28(4):807–815, 2013.

[183] R.S. Hinman, K.D. Allen, K.L. Bennell, F. Berenbaum, N. Betteridge, A.M. Briggs, P.K. Campbell, L.E. Dahlberg, K.S. Dziedzic, J.P. Eyles, D.J. Hunter, S.T. Skou, A. Woolf, S.P. Yu, and M. van der Esch. Development of a core capability framework for qualified health professionals to optimise care for people with osteoarthritis: an oarsi initiative. *Osteoarthritis and Cartilage*, 28(2):154–166, 2020.

[184] E. Hohmann, R. Angelo, R. Arciero, B.R. Bach, B. Cole, M. Cote, J. Farr, J. Feller, B. Gelbhart, A. Gomoll, A. Imhoff, R. LaPrade, B.R. Mandelbaum, R.G. Marx, J.C. Monllau, F. Noyes, D. Parker, S. Rodeo, N. Sgaglione, K. Shea, D.K. Shelbourne, S. Yoshiya, V. Glatt, and K. Tetsworth. Degenerative meniscus lesions: An expert consensus statement using the modified delphi technique. *Arthroscopy - Journal of Arthroscopic and Related Surgery*, 36(2):501–512, 2020.

[185] J. Honorato, A. Gómez-Outes, A. Navarro-Quilis, J. Martínez-González, E. Rocha, and A. Planès. Pharmacoeconomic analysis of bemiparin and enoxaparin as prophylaxis for venous thromboembolism in total knee replacement surgery. *PharmacoEconomics*, 22(13):885–894, 2004.

[186] T.J. Hopkins, S. Eggington, M. Quinn, and C.I. Nichols-Ricker. Cost-effectiveness of balloon kyphoplasty and vertebroplasty versus conservative medical management in the usa. *Osteoporosis International*, 31(12):2461–2471, 2020.

[187] A.L. Huang, K. Thavorn, S. Van Katwyk, P. MacDonald, and P. Lapner. Double-row arthroscopic rotator cuff repair is more cost-effective than single-row repair. *Journal of Bone and Joint Surgery - American Volume*, 99(20):1730–1736, 2017.

[188] B.M.A. Huisstede, P. Hoogvliet, J. Henk Coert, and J. Fridén. Multidisciplinary consensus guideline for managing trigger finger: Results from the european handguide study. *Physical Therapy*, 94(10):1421–1433, 2014.

[189] J.M. Hummel, I.S.M. Boomkamp, L.M.G. Steuten, B.G.J. Verkerke, and M.J. Ijzerman. Predicting the health economic performance of new non-fusion surgery in adolescent idiopathic scoliosis. *Journal of Orthopaedic Research*, 30(9):1453–1458, 2012.

[190] D.J. Hurley, M.S. Davey, E.T. Hurley, C.D. Murawski, J.D.F. Calder, P. D'Hooghe, C.J.A. van Bergen, R.J. Walls, Z. Ali, J.N. Altink, J. Batista, S. Bayer, G.C. Berlet, R. Buda, J. Dahmen, C.W. DiGiovanni, R.D. Ferkel, A.L. Gianakos, E. Giza, M. Glazebrook, S. Guillo, L. Hangody, D. Haverkamp, B. Hintermann, M.V. Hogan, Y. Hua, K. Hunt, M.S. Jamal, J. Karlsson, S. Kearns, G.M.M.J. Kerkhoffs, K. Lambers, J.W. Lee, G. McCollum, N.P. Mercer, C. Mulvin, J.A. Nunley, J. Paul, C. Pearce, H. Pereira, M. Prado, S.M. Raikin, I. Savage-

Elliott, L.C. Schon, Y. Shimozono, J.W. Stone, S.A.S. Stufkens, M. Sullivan, M. Takao, H. Thermann, D. Thordarson, J. Toale, V. Valderrabano, F. Vannini, C.N. van Dijk, M. Walther, Y. Yasui, A.S. Younger, and J.G. Kennedy. Paediatric ankle cartilage lesions: Proceedings of the international consensus meeting on cartilage repair of the ankle. *Journal of ISAKOS*, 7(5):90–94, 2022.

[191] C.A. Jacobs, J.M. Burnham, E. Makhni, C.S. Malempati, E. Swart, and D.L. Johnson. Allograft augmentation of hamstring autograft for younger patients undergoing anterior cruciate ligament reconstruction: Clinical and cost-effectiveness analyses. *American Journal of Sports Medicine*, 45(4):892–899, 2017.

[192] J.J. Jacobson, S.O. Schweitzer, and C.J. Kowalski. Chemoprophylaxis of prosthetic joint patients during dental treatment: A decision-utility analysis. *Oral Surgery, Oral Medicine, Oral Pathology*, 72(2):167–177, 1991.

[193] A. Jain, M.C. Marks, M.P. Kelly, L.G. Lenke, T.J. Errico, B.S. Lonner, P.O. Newton, and P.D. Sponseller. Cost-utility analysis of operative versus nonoperative treatment of thoracic adolescent idiopathic scoliosis. *Spine*, 44(5):309–317, 2019.

[194] A. Jain, S. Yeramaneni, K.M. Kebaish, M. Raad, J.L. Gum, E.O. Klineberg, H. Hassanzadeh, M.P. Kelly, P.G. Passias, C.P. Ames, J.S. Smith, C.I. Shaffrey, S. Bess, V. Lafage, S. Glassman, L.Y. Carreon, and R.A. Hostin. Cost-utility analysis of rhbmp-2 use in adult spinal deformity surgery. *Spine*, 45(14):1009–1015, 2020.

[195] M. Javanbakht, A. Mashayekhi, A. Carlson, E. Moloney, M. Snow, J. Murray, and T. Spalding. Cost-effectiveness analysis of a medial meniscus replacement prosthesis for the treatment of patients with medial compartment pain in the united kingdom. *PharmacoEconomics - Open*, 6(5):681–696, 2022.

[196] S. Jerabkova, P. Fulin, J. Vacek, and H. Holubova. Shoulder joint arthroplasty cost-effectiveness in the czech republic. Institute of Electrical and Electronics Engineers Inc., 2016.

[197] S.Y. Jiang, D.J. Kaufman, B.Y. Chien, M. Longoria, R. Shachter, and J.A. Bishop. Prophylactic fixation can be cost-effective in preventing a contralateral bisphosphonate-associated femur fracture. *Clinical Orthopaedics and Related Research*, 477(3):480–490, 2019.

[198] B. Johnson, E.C.-C. Lai, H.-T. Ou, H. Li, and B. Stollenwerk. Real-world cost-effectiveness of denosumab for the treatment of postmenopausal osteoporosis in taiwan. *Archives of Osteoporosis*, 16(1), 2021.

[199] C.B. Johnson. A personalized shared decision-making tool for osteoarthritis management of the knee. *Orthopaedic Nursing*, 40(2):64–70, 2021.

[200] G. Johnson, J. Kiernan, A. Swan, E. Botwick, W. Spier, Jr. White, K.P., J. Valdez, H. Kang, and J.M. Lobo. Designing patient throughput and task management innovations in orthopaedics. pages 124–129. Institute of Electrical and Electronics Engineers Inc., 2016.

[201] S.B. Kailes and J.C. Richmond. Arthroscopic vs. open bankart reconstruction: A comparison using expected value decision analysis. *Knee Surgery, Sports Traumatology, Arthroscopy*, 9(6):379–385, 2001.

[202] Y.E. Kaliberda, V.N. Leonenko, and V.A. Artyukh. Towards cost-effective treatment of periprosthetic joint infection: From statistical analysis to markov models. *Lecture Notes in Computer Science (including subseries Lecture Notes in Artificial Intelligence and Lecture Notes in Bioinformatics)*, 12744 LNCS:494–505, 2021.

[203] A. Kamaraj, N. Agarwal, K.T. Matthew Seah, and W. Khan. Understanding cost-utility analysis studies in the trauma and orthopaedic surgery literature. *EFORT Open Reviews*, 6(5):305–315, 2021.

[204] J.R. Kang, A.T. Sin, and E.V. Cheung. Treatment of massive irreparable rotator cuff tears: A cost-effectiveness analysis. *Orthopedics*, 40(1):e65–e76, 2017.

[205] J.A. Kanis, J. Adams, F. Borgström, C. Cooper, B. Jönsson, D. Preedy, P. Selby, and J. Compston. The cost-effectiveness of alendronate in the management of osteoporosis. *Bone*, 42(1):4–15, 2008.

[206] U. Karamchandani, R. Bhattacharyya, R. Patel, S. Oussedik, R. Bhattacharya, and C. Gupte. Training surgeons to perform arthroscopic all-inside meniscal repair: A randomized controlled trial evaluating the effectiveness of a novel cognitive task analysis teaching tool, imperial college london/university college london meniscus repair cognitive task analysis (iumercta). *American Journal of Sports Medicine*, 49(9):2341–2350, 2021.

[207] D. Karczewski, Y. Ren, O. Andronic, D. Akgün, C. Perka, M. Müller, and A. Kienzle. Candida periprosthetic joint infections — risk factors and outcome between albicans and non-albicans strains. *International Orthopaedics*, 46(3):449–456, 2022.

[208] T.D. Karmarkar, A. Maurer, M.L. Parks, T. Mason, A. Bejinez-Eastman, M. Harrington, R. Morgan, M.I. O'Connor, J.E. Wood, and D.J. Gaskin. A fresh perspective on a familiar problem. *Medical Care*, 55(12):993–1000, 2017.

[209] J. Karnon, B.M. Haghighi, B. Sajjad, S. Yem, A. Gamage, and A. Thorpe. Cost-utility analysis of private contracting to reduce public waiting times for joint replacement surgery. *International Journal of Technology Assessment in Health Care*, 34(2):147–155, 2018.

[210] B.G. Kaufman, K.D. Allen, C.J. Coffman, S. Woolson, K. Caves, K. Hall, H.M. Hoenig, K.M. Huffman, M.C. Morey, N.J. Hodges, S. Ramasunder, and C.H. van Houtven. Cost and quality of life outcomes of the stepped exercise program for patients with knee osteoarthritis trial. *Value in Health*, 25(4):614–621, 2022.

[211] R.M. Kay, K. Pierz, J. McCarthy, H.K. Graham, H. Chambers, J.R. Davids, U. Narayanan, T.F. Novacheck, J. Rhodes, E. Rutz, J. Shilt, B.J. Shore, M. Veerkamp, M.W. Shrader, T. Theologis, A. Van Campenhout, and T. Dreher. Distal rectus femoris surgery in children with cerebral palsy: Results of a delphi consensus project. *Journal of Children's Orthopaedics*, 15(3):270–278, 2021.

[212] G.S. Kazarian, J.H. Lonner, M.G. Maltenfort, H.M.K. Ghomrawi, and A.F. Chen. Cost-effectiveness of surgical and nonsurgical treatments for unicompartmental knee arthritis: Markov model. *Journal of Bone and Joint Surgery - American Volume*, 100(19):1653–1660, 2018.

[213] J.F. Kellam. The core competencies for general orthopaedic surgeons. *Journal of Bone and Joint Surgery - American Volume*, 99(2):175–181, 2017.

[214] B. Keren and J.S. Pliskin. Optimal timing of joint replacement using mathematical programming and stochastic programming models. *Health Care Management Science*, 14(4):361–369, 2011.

[215] C.L. Kerrigan and M.G. Stanwix. Using evidence to minimize the cost of trigger finger care. *Journal of Hand Surgery*, 34(6):997–1005, 2009.

[216] A. Keshmiri, F. Dirisamer, M. Liebensteiner, R. El Attal, G. Pagenstert, G. Seitlinger, D. Wagner, P. Balcarek, P. Kappel, P. Schöttle, J. Frings, P. Tscholl, C. Becher, and the AGA Patellofemoral Committee. Operative treatment options for patellofemoral arthritis: An expert recommendation of the aga patellofemoral committee. *Orthopaedic Journal of Sports Medicine*, 9(3), 2021.

[217] M.M. Kheir, T.L. Tan, M.M. Gomez, A.F. Chen, and J. Parvizi. Patients with failed prior two-stage exchange have poor outcomes after further surgical intervention. *Journal of Arthroplasty*, 32(4):1262–1265, 2017.

[218] A. Khoshbin, F.S. Haddad, S. Ward, S. O'hEireamhoin, J. Wu, L. Nherera, and A. Atrey. A cost-effectiveness assessment of dual-mobility bearings in revision hip arthroplasty. *Bone and Joint Journal*, 102B(9):1128–1135, 2020.

[219] J.S. Kim, J. Dowdell, Z.B. Cheung, V. Arvind, L. Sun, C. Jandhyala, C. Ukogu, W. Ranson, S. Jacobs, S. McAnany, and S.K.-W. Cho. The seven-year cost-effectiveness of anterior cervical discectomy and fusion versus cervical disc arthroplasty: A markov analysis. *Spine*, 43(22):1543–1551, 2018.

[220] S. Kim, S. Mortaz Hedjri, P.C. Coyte, and Y.R. Rampersaud. Cost-utility of lumbar decompression with or without fusion for patients with symptomatic degenerative lumbar spondylolisthesis. *Spine Journal*, 12(1):44–54, 2012.

[221] C. Kittipittayakorn and K.-C. Ying. Using the integration of discrete event and agent-based simulation to enhance outpatient service quality in an orthopedic department. *Journal of Healthcare Engineering*, 2016, 2016.

[222] S. Klouche, P. Leonard, V. Zeller, L. Lhotellier, W. Graff, P. Leclerc, P. Mamoudy, and E. Sariali. Infected total hip arthroplasty revision: One- or two-stage procedure? *Orthopaedics and Traumatology: Surgery and Research*, 98(2):144–150, 2012.

[223] M.S. Kocher, J. Bishop, R. Marshall, K.K. Briggs, and R.J. Hawkins. Operative versus nonoperative management of acute achilles tendon rupture: Expected-value decision analysis. *American Journal of Sports Medicine*, 30(6):783–790, 2002.

[224] M.S. Kocher, J.A. Bishop, M.T. Hresko, M.B. Millis, Y.-J. Kim, and J.R. Kasser. Prophylactic pinning of the contralateral hip after unilateral slipped capital femoral epiphysis. *Journal of Bone and Joint Surgery - Series A*, 86(12):2658–2665, 2004.

[225] R.J. Koehler, S. Amsdell, E.A. Arendt, L.J. Bisson, J.P. Bramen, A. Butler, A.J. Cosgarea, C.D. Harner, W.E. Garrett, T. Olson, W.J. Warme, and G.T. Nicandri. The arthroscopic surgical skill evaluation tool (asset). *American Journal of Sports Medicine*, 41(6):1229–1237, 2013.

[226] K.M. Koenig, G.C. Davis, M.R. Grove, A.N.A. Tosteson, and K.J. Koval. Is early internal fixation preferred to cast treatment for well-reduced unstable distal radial fractures? *Journal of Bone and Joint Surgery - Series A*, 91(9):2086–2093, 2009.

[227] L. Koenig, T.M. Dall, Q. Gu, J. Saavoss, and M.F. Schafer. How does accounting for worker productivity affect the measured cost-effectiveness of lumbar discectomy? *Clinical Orthopaedics and Related Research*, 472(4):1069–1079, 2014.

[228] L. Koenig, T.M. Dall, Jr. Ruiz, D., J. Saavoss, and J. Tongue. Can value-based insurance impose societal costs? *Value in Health*, 17(6):749–751, 2014.

[229] L. Koenig, C. Feng, F. He, and J.T. Nguyen. The effects of revision total hip arthroplasty on medicare spending and beneficiary outcomes: Implications for the comprehensive care for joint replacement model. *Journal of Arthroplasty*, 33(9):2764–2769.e2, 2018.

[230] L. Koenig, Q. Zhang, M.S. Austin, B. Demiralp, T.K. Fehring, C. Feng, III Mather, R.C., J.T. Nguyen, A. Saavoss, B.D. Springer, and Jr. Yates, A.J. Estimating the societal benefits of tha after accounting for work status and productivity: A markov model approach. *Clinical Orthopaedics and Related Research*, 474(12):2645–2654, 2016.

[231] J.C.B. Koltsov, C. Gribbin, S.J. Ellis, and B.U. Nwachukwu. Cost-effectiveness of operative versus non-operative management of acute achilles tendon ruptures. *HSS Journal*, 16(1):39–45, 2020.

[232] J.F. Konopka, A.H. Gomoll, T.S. Thornhill, J.N. Katz, and E. Losina. The cost-effectiveness of surgical treatment of medial unicompartmental knee osteoarthritis in younger patients a computer model-based evaluation. *Journal of Bone and Joint Surgery - American Volume*, 97(10):807–817, 2014.

[233] J.A. Kopec, E.C. Sayre, A. Okhmatovskaia, J. Cibere, L.C. Li, N. Bansback, H. Wong, S. Ghanbarian, and J.M. Esdaile. A comparison of three strategies to reduce the burden of osteoarthritis: A populationbased microsimulation study. *PLoS ONE*, 16(12 December), 2021.

[234] S. Kotirum, B. Chongmelaxme, and N. Chaiyakunapruk. A cost-utility analysis of dabigatran, enoxaparin, and usual care for venous thromboprophylaxis after hip or knee replacement surgery in thailand. *Journal of Thrombosis and Thrombolysis*, 43(2):252–262, 2017.

[235] S.T. Kunkel, M.J. Sabatino, R. Kang, D.S. Jevsevar, and W.E. Moschetti. The cost-effectiveness of total hip arthroplasty in patients 80 years of age and older. *Journal of Arthroplasty*, 33(5):1359–1367, 2018.

[236] K.M. Kuntz, R.K. Snider, J.N. Weinstein, M.H. Pope, and J.N. Katz. Cost-effectiveness of fusion with and without instrumentation for patients with degenerative spondylolisthesis and spinal stenosis. *Spine*, 25(9):1132–1139, 2000.

[237] N. Kusnezov, E.D. Eisenstein, J.C. Dunn, A.J. Wey, D.R. Peterson, and B.R. Waterman. Anterior cruciate ligament graft removal versus retention in the setting of septic arthritis after reconstruction: A systematic review and expected value decision analysis. *Arthroscopy - Journal of Arthroscopic and Related Surgery*, 34(3):967–975, 2018.

[238] D.G. Kwon, C.Y. Chung, M.S. Park, K.H. Sung, T.W. Kim, and K.M. Lee. Arthroplasty versus arthrodesis for end-stage ankle arthritis: Decision analysis using markov model. *International Orthopaedics*, 35(11):1647–1653, 2011.

[239] K.N. Lam, N.R. Heneghan, J. Mistry, A.O. Ojoawo, A. Peolsson, A.P. Verhagen, B. Tampin, E. Thoomes, G. Jull, G.G.M. Scholten Peeters, H. Slater, N. Moloney, T. Hall, Å. Dedering, A. Rushton, and D. Falla. Classification criteria for cervical radiculopathy: An international e-delphi study. *Musculoskeletal Science and Practice*, 61, 2022.

[240] R.H. Lan, J. Yu, L.T. Samuel, M.A. Pappas, P.J. Brooks, and A.F. Kamath. How are we measuring cost-effectiveness in total joint arthroplasty studies? systematic review of the literature. *Journal of Arthroplasty*, 35(11):3364–3374, 2020.

[241] A. Lazo-Langner, M.A. Rodger, N.J. Barrowman, T. Ramsay, P.S. Wells, and D.A. Coyle. Comparing multiple competing interventions in the absence of randomized trials using clinical risk-benefit analysis. *BMC Medical Research Methodology*, 12, 2012.

[242] D.G. LeBrun, C.J. DeFrancesco, P.D. Fabricant, and J.T.R. Lawrence. Cost-effectiveness analysis of nonoperative management versus early drilling for stable osteochondritis dissecans lesions of the knee in skeletally immature patients. *Arthroscopy - Journal of Arthroscopic and Related Surgery*, 37(2):624–634.e2, 2021.

[243] D.G. LeBrun, B.U. Nwachukwu, S.S. Buza, S. Gruber, W.A. Marmor, E.R. Dennis, and B.E. Shubin Stein. Particulated juvenile articular cartilage and matrix-induced autologous chondrocyte implantation are cost-effective for patellar chondral lesions. *Arthroscopy - Journal of Arthroscopic and Related Surgery*,

38(4):1252–1263.e3, 2022.

[244] H. Lee, U.C. Kim, J.K. Oh, T. Kim, S. Park, and Y. Ha. Cost-effectiveness analysis of cervical anterior fusion and cervical artificial disc replacement in the korean medical system. *Journal of Korean Neurosurgical Society*, 62(1):83–89, 2019.

[245] H.K. Lee, J. Li, P.A. Bain, and C. Baker. Optimal intervention policies for total joint replacement postoperative care process. *Manufacturing and Service Operations Management*, 24(1):617–631, 2022.

[246] K.M. Lee, C.Y. Chung, D.K. Gwon, K.H. Sung, T.W. Kim, I.H. Choi, T.-J. Cho, W.J. Yoo, and M.S. Park. Medial and lateral crossed pinning versus lateral pinning for supracondylar fractures of the humerus in children: Decision analysis. *Journal of Pediatric Orthopaedics*, 32(2):131–138, 2012.

[247] Y.-K. Lee, C.Y. Chung, M.S. Park, K.M. Lee, and K.-H. Koo. Intramedullary nail versus extramedullary plate fixation for unstable intertrochanteric fractures: Decision analysis. *Archives of Orthopaedic and Trauma Surgery*, 133(7):961–968, 2013.

[248] V.N. Leonenko, Y.E. Kaliberda, and V.A. Artyuk. A modeling framework for decision support in periprosthetic joint infection treatment. *Studies in Health Technology and Informatics*, 285:106–111, 2021.

[249] V.N. Leonenko, Y.E. Kaliberda, Y.V. Muravyova, and V.A. Artyukh. A decision support framework for periprosthetic joint infection treatment: A cost-effectiveness analysis using two modeling approaches †. *Journal of Personalized Medicine*, 12(8), 2022.

[250] J.D. Lester, J.D. Gorbaty, S.M. Odum, M.E. Rogers, and J.E. Fleischli. The cost-effectiveness of meniscal repair versus partial meniscectomy in the setting of anterior cruciate ligament reconstruction. *Arthroscopy - Journal of Arthroscopic and Related Surgery*, 34(9):2614–2620, 2018.

[251] D.A. Lewis, B. Kirkbride, C.J. Vertullo, L. Gordon, and T.A. Comans. Comparison of four alternative national universal anterior cruciate ligament injury prevention programme implementation strategies to reduce secondary future medical costs. *British Journal of Sports Medicine*, 52(4):277–282, 2018.

[252] D.J. Lewis, M.A. Attiah, N.R. Malhotra, M.G. Burnett, and S.C. Stein. Anterior surgical management of single-level cervical disc disease: A cost-effectiveness analysis. *Spine*, 39(25):2084–2092, 2014.

[253] D. Lewkowicz, A.M. Wohlbrandt, and E. Bottinger. Digital therapeutic care apps with decision-support interventions for people with low back pain in germany: Cost-effectiveness analysis. *JMIR mHealth and uHealth*, 10(2), 2022.

[254] J. Liu, J. Farr, O. Ramos, J. Voigt, and N. Amin. Workers' societal costs after knee and shoulder injuries and diagnosis with in-office arthroscopy or delayed mri a cost-minimization analysis. *JBJS Open Access*, 6(2), 2021.

[255] J. Liu, K. Srivastava, T. Washington, J. Hoegler, S.T. Guthrie, W. Hakeos, and V. Moutzouros. Cost-effectiveness of operative versus nonoperative treatment of displaced midshaft clavicle fractures: A decision analysis. *Journal of Bone and Joint Surgery - American Volume*, 101(1):35–47, 2019.

[256] P. Lodhia, C. Gui, S. Chandrasekaran, C. Suarez-Ahedo, D.R. Dirschl, and B.G. Domb. The economic impact of acetabular labral tears. *American Journal of Sports Medicine*, 44(7):1771–1780, 2016.

[257] E. Losina, R.P. Walensky, C.L. Kessler, P.S. Emrani, W.M. Reichmann, E.A. Wright, H.L. Holt, D.H. Solomon, E. Yelin, A.D. Paltiel, and J.N. Katz. Cost-effectiveness of total knee arthroplasty in the united states: Patient risk and

hospital volume. *Archives of Internal Medicine*, 169(12):1113–1121, 2009.

[258] A. Ltaif, A. Ammar, and L. Khrifch. A goal programming approach based on simulation and optimization to serve patients in an external orthopedic department. *Journal of Simulation*, 2022.

[259] T.-P. Lu, P.-F. Tsai, and Y.-C. Chu. An agent-based collaborative model for orthopedic outpatient scheduling. pages 621–626. IEEE Computer Society, 2014.

[260] T.S. Lynch, A. Minkara, S. Aoki, A. Bedi, S. Bharam, J. Clohisy, J. Harris, C. Larson, J. Nepple, S. Nho, M. Philippon, J. Rosneck, M. Safran, A.J. Stubbs, R. Westermann, and J.W.T. Byrd. Best practice guidelines for hip arthroscopy in femoroacetabular impingement: Results of a delphi process. *Journal of the American Academy of Orthopaedic Surgeons*, 28(2):81–89, 2020.

[261] T. MacKinnon, H. Selmi, A. Davies, T.W. Packer, P. Reilly, K.M. Sarraf, and S. Sabharwal. Protocolised mri as an adjunct to ct in the diagnosis of femoral neck fracture in high energy ipsilateral femoral shaft fractures – a break-even analysis. *Injury*, 2022.

[262] S.T. Mahan, J.N. Katz, and Y.-J. Kim. To screen or not to screen? a decision analysis of the utility of screening for developmental dysplasia of the hip. *Journal of Bone and Joint Surgery - Series A*, 91(7):1705–1719, 2009.

[263] M. Mahmoudi and D.M. Sobieraj. The cost-effectiveness of oral direct factor xa inhibitors compared with low-molecular-weight heparin for the prevention of venous thromboembolism prophylaxis in total hip or knee replacement surgery. *Pharmacotherapy*, 33(12):1333–1340, 2013.

[264] S.R. Majumdar, D.A. Lier, L.A. Beaupre, D.A. Hanley, W.P. Maksymowych, A.G. Juby, N.R. Bell, and D.W. Morrish. Osteoporosis case manager for patients with hip fractures: Results of a cost-effectiveness analysis conducted alongside a randomized trial. *Archives of Internal Medicine*, 169(1):25–31, 2009.

[265] S.R. Majumdar, D.A. Lier, F.A. McAlister, B.H. Rowe, K. Siminoski, D.A. Hanley, A.S. Russell, and J.A. Johnson. Cost-effectiveness of osteoporosis interventions for 'incidental' vertebral fractures. *American Journal of Medicine*, 126(2):169.e9–169.e17, 2013.

[266] S.R. Majumdar, D.A. Lier, B.H. Rowe, A.S. Russell, F.A. McAlister, W.P. Maksymowych, D.A. Hanley, D.W. Morrish, and J.A. Johnson. Cost-effectiveness of a multifaceted intervention to improve quality of osteoporosis care after wrist fracture. *Osteoporosis International*, 22(6):1799–1808, 2011.

[267] E.C. Makhni, N. Lamba, E. Swart, M.E. Steinhaus, C.S. Ahmad, A.A. Romeo, and N.N. Verma. Revision arthroscopic repair versus latarjet procedure in patients with recurrent instability after initial repair attempt: A cost-effectiveness model. *Arthroscopy - Journal of Arthroscopic and Related Surgery*, 32(9):1764–1770, 2016.

[268] E.C. Makhni, E. Swart, M.E. Steinhaus, III Mather, R.C., W.N. Levine, Jr. Bach, B.R., A.A. Romeo, and N.N. Verma. Cost-effectiveness of reverse total shoulder arthroplasty versus arthroscopic rotator cuff repair for symptomatic large and massive rotator cuff tears. *Arthroscopy - Journal of Arthroscopic and Related Surgery*, 32(9):1771–1780, 2016.

[269] M. Marchetti, N.L. Liberato, N. Ruperto, and G. Barosi. Long-term cost-effectiveness of low molecular weight heparin versus unfractionated heparin for the prophylaxis of venous thromboembolism in elective hip replacement. *Haematologica*, 84(8):730–737, 1999.

[270] K. Mari, P. Dégieux, F. Mistretta, F. Guillemin, and P. Richette. Cost utility modeling of early vs late total knee replacement in osteoarthritis patients.

Osteoarthritis and Cartilage, 24(12):2069–2076, 2016.

[271] M. Marinelli, A. Soccetti, N. Panfoli, and L. Palma. Cost-effectiveness of cemented versus cementless total hip arthroplasty. a markov decision analysis based on implant cost. *Journal of Orthopaedics and Traumatology*, 9(1):23–28, 2008.

[272] L.M. Marti-Martinez, A. Gracia-Sánchez, J. Ferrer-Torregrosa, R. Lorca-Gutierrez, J. Garcia-Campos, and S.P. Sánchez-Pérez. Description of the surgical technique for condylectomy with minimally invasive surgery to treat interdigital helomas on the lesser toes: A delphi study. *Journal of Foot and Ankle Research*, 12(1), 2019.

[273] R.C. Mather, W.E. Garrett, B.J. Cole, K. Hussey, M.P. Bolognesi, T. Lassiter, and L.A. Orlando. Cost-effectiveness analysis of the diagnosis of meniscus tears. *American Journal of Sports Medicine*, 43(1):128–137, 2015.

[274] R.C. Mather, K.T. Hug, L.A. Orlando, T.S. Watters, L. Koenig, R.M. Nunley, and M.P. Bolognesi. Economic evaluation of access to musculoskeletal care: The case of waiting for total knee arthroplasty. *BMC Musculoskeletal Disorders*, 15(1), 2014.

[275] R.C. Mather, L. Koenig, D. Acevedo, T.M. Dall, P. Gallo, A. Romeo, J. Tongue, and G. Williams Jr. The societal and economic value of rotator cuff repair. *Journal of Bone and Joint Surgery - Series A*, 95(22):1993–2000, 2013.

[276] R.C. Mather, L. Koenig, M.S. Kocher, T.M. Dall, P. Gallo, D.J. Scott, B.R. Bach Jr., and K.P. Spindler. Societal and economic impact of anterior cruciate ligament tears. *Journal of Bone and Joint Surgery - American Volume*, 95(19):1751–1759, 2013.

[277] R.C. Mather III, C.M. Hettrich, W.R. Dunn, B.J. Cole, B.R. Bach Jr., L.J. Huston, E.K. Reinke, and K.P. Spindler. Cost-effectiveness analysis of early reconstruction versus rehabilitation and delayed reconstruction for anterior cruciate ligament tears. *American Journal of Sports Medicine*, 42(7):1583–1591, 2014.

[278] R.C. Mather III, T.S. Watters, L.A. Orlando, M.P. Bolognesi, and C.T. Moorman III. Cost effectiveness analysis of hemiarthroplasty and total shoulder arthroplasty. *Journal of Shoulder and Elbow Surgery*, 19(3):325–334, 2010.

[279] S.J. McAnany, M.A.F. Anwar, and S.A. Qureshi. Decision analytic modeling in spinal surgery: A methodologic overview with review of current published literature. *Spine Journal*, 15(10):2254–2270, 2015.

[280] S.J. McAnany, R.K. Merrill, S.C. Overley, J.S. Kim, R.L. Brochin, and S.A. Qureshi. Investigating the 7-year cost-effectiveness of single-level cervical disc replacement compared to anterior cervical discectomy and fusion. *Global Spine Journal*, 8(1):32–39, 2018.

[281] S.J. McAnany, S. Overley, E.O. Baird, S.K. Cho, A.C. Hecht, J.E. Zigler, and S.A. Qureshi. The 5-year cost-effectiveness of anterior cervical discectomy and fusion and cervical disc replacement: A markov analysis. *Spine*, 39(23):1924–1933, 2014.

[282] M.P. McClincy, J.D. Wylie, D.N. Williams, and E.N. Novais. Standardizing the diagnostic evaluation of nonarthritic hip pain through the delphi method. *Orthopaedic Journal of Sports Medicine*, 9(4), 2021.

[283] L. McCullagh, L. Tilson, C. Walsh, and M. Barry. A cost-effectiveness model comparing rivaroxaban and dabigatran etexilate with enoxaparin sodium as thromboprophylaxis after total hip and total knee replacement in the irish healthcare setting. *PharmacoEconomics*, 27(10):829–846, 2009.

[284] H. McDonald, A. Diamantopoulos, P. Wells, M. Lees, K. Folkerts, F. Forster, and J. Ananthapavan. Cost-effectiveness of rivaroxaban in the prevention of venous thromboembolism: A canadian analysis using the ontario ministry of health perspective. *Journal of Medical Economics*, 15(5):817–828, 2012.

[285] L. McKenzie, L. Vale, S. Stearns, and K. McCormack. Metal on metal hip resurfacing arthroplasty: An economic analysis. *European Journal of Health Economics*, 4(2):122–129, 2003.

[286] R.J. McLaughlin, A. Miniaci, and M.H. Jones. Bony versus soft tissue reconstruction for anterior shoulder instability: An expected value decision analysis. *Orthopaedic Journal of Sports Medicine*, 3(12), 2015.

[287] A.S. McLawhorn, K.M. Carroll, J.L. Blevins, S.T. DeNegre, D.J. Mayman, and S.A. Jerabek. Template-directed instrumentation reduces cost and improves efficiency for total knee arthroplasty: An economic decision analysis and pilot study. *Journal of Arthroplasty*, 30(10):1699–1704, 2015.

[288] A.S. McLawhorn, D. Southren, Y.C. Wang, R.G. Marx, and E.R. Dodwell. Cost-effectiveness of bariatric surgery prior to total knee arthroplasty in the morbidly obese a computer model-based evaluation. *Journal of Bone and Joint Surgery - American Volume*, 98(2):e6, 2016.

[289] M.P. Mehta, M.A. Hoffer-Hawlik, M. O'Connor, and T.S. Lynch. Immediate versus delayed hip arthroscopy for femoroacetabular impingement: An expected value decision analysis. *Journal of the American Academy of Orthopaedic Surgeons Global Research and Reviews*, 4(12), 2020.

[290] K.A. Meijer and V. Dasa. Is resurfacing the patella cheaper? an economic analysis of evidence based medicine on patellar resurfacing. *Knee*, 22(2):136–141, 2015.

[291] L.J. Melton III, M. Thamer, N.F. Ray, J.K. Chan, C.H. Chesnut III, T.A. Einhorn, C.C. Johnston, L.G. Raisz, S.L. Silverman, and E.S. Siris. Fractures attributable to osteoporosis: Report from the national osteoporosis foundation. *Journal of Bone and Mineral Research*, 12(1):16–23, 1997.

[292] J. Menzin, G.A. Colditz, M.M. Regan, R.E. Richner, and G. Oster. Cost-effectiveness of enoxaparin vs low-dose warfarin in the prevention of deep-vein thrombosis after total hip replacement surgery. *Archives of Internal Medicine*, 155(7):757–764, 1995.

[293] S.J. Meredith, T. Rauer, T.L. Chmielewski, C. Fink, T. Diermeier, B.B. Rothrauff, E. Svantesson, E. Hamrin Senorski, T.E. Hewett, S.L. Sherman, B.P. Lesniak, and The Panther Symposium ACL Injury Return to Sport Consensus Group. Return to sport after anterior cruciate ligament injury: Panther symposium acl injury return to sport consensus group. *Knee Surgery, Sports Traumatology, Arthroscopy*, 28(8):2403–2414, 2020.

[294] K.M.D. Merollini, R.W. Crawford, S.L. Whitehouse, and N. Graves. Surgical site infection prevention following total hip arthroplasty in australia: A cost-effectiveness analysis. *American Journal of Infection Control*, 41(9):803–809, 2013.

[295] R.K. Merrill, S.J. McAnany, T.J. Albert, and S.A. Qureshi. Is two-level cervical disc replacement more cost-effective than anterior cervical discectomy and fusion at 7 years? *Spine*, 43(9):610–616, 2018.

[296] A.J. Meyr, R. Mirmiran, J. Naldo, B.D. Sachs, and N. Shibuya. American college of foot and ankle surgeons® clinical consensus statement: Perioperative management. *Journal of Foot and Ankle Surgery*, 56(2):336–356, 2017.

[297] J.D. Michelson. Using decision analysis to assess comparative clinical efficacy of

surgical treatment of unstable ankle fractures. *Journal of Orthopaedic Trauma*, 27(11):642–648, 2013.

[298] A. Migliore, D. Integlia, G. Pompilio, F. Di Giuseppe, C. Aru, and T. Brown. Cost-effectiveness and budget impact analysis of viscosupplementation with hylan g-f 20 for knee and hip osteoarthritis. *ClinicoEconomics and Outcomes Research*, 11:453–464, 2019.

[299] K. Min, C. Fedorka, M.J. Solberg, S.H. Shaha, and L.D. Higgins. The cost-effectiveness of the arthroscopic bankart versus open latarjet in the treatment of primary shoulder instability. *Journal of Shoulder and Elbow Surgery*, 27(6):S2–S9, 2018.

[300] P.N. Mittwede, C.D. Murawski, J. Ackermann, S. Görtz, B. Hintermann, H.J. Kim, D.B. Thordarson, F. Vannini, A.S.E. Younger, Jr. Adams, S.B., C.L. Andrews, C. Angthong, J.P. Batista, O.L. Baur, S. Bayer, C. Becher, G.C. Berlet, L.A.T. Boakye, A.J. Brown, R. Buda, J.D.F. Calder, G.L. Canata, D.S. Carreira, T.O. Clanton, J. Dahmen, P. D'Hooghe, C.W. DiGiovanni, M.E. Dombrowski, M.C. Drakos, R.D. Ferkel, P.N.F. Ferrao, L.A. Fortier, M. Glazebrook, E. Giza, M. Gomaa, A.M. Haleem, K. Hamid, L. Hangody, C.P. Hannon, D. Haverkamp, J. Hertel, M.V. Hogan, K.J. Hunt, E.T. Hurley, J. Karlsson, S.R. Kearns, J.G. Kennedy, G.M.M.J. Kerkhoffs, S.W. Kong, S.A. Labib, K.T.A. Lambers, J.W. Lee, K.B. Lee, J.S. Ling, U.G. Longo, A. Marangon, G. McCollum, A.W. Mitchell, S. Nehrer, P. Niemeyer, II Nunley, J.A., M.J. O'Malley, D.O. Osei-Hwedieh, J. Paul, C.J. Pearce, H. Pereira, A. Popchak, M.P. Prado, S.M. Raikin, M.L. Reilingh, B.B. Rothrauff, L.C. Schon, Y. Shimozono, H. Simpson, N.A. Smyth, C.M. Sofka, P. Spennacchio, J.W. Stone, M. Sullivan, M. Takao, Y. Tanaka, R. Tuan, V. Valderrabano, C.J.A. van Bergen, C.N. van Dijk, P.A.D. van Dijk, T. Vaseenon, M. Walther, M. Wiewiorski, X. Xu, Y. Yasui, H. Yinghui, I. Yoshimura, Z. Zhang, and the International Consensus Group on Cartilage Repair of the Ankle. Revision and salvage management: Proceedings of the international consensus meeting on cartilage repair of the ankle. *Foot and Ankle International*, 39(1_suppl):54S–60S, 2018.

[301] A. Mohandas, C. Summa, W.B. Worthington, J. Lerner, K.T. Foley, R.J. Bohinski, G.B. Lanford, C. Holden, and R.N.W. Wohns. Best practices for outpatient anterior cervical surgery. *Spine*, 42(11):E648–E659, 2017.

[302] M. Monreal, K. Folkerts, A. Diamantopoulos, D. Imberti, and M. Brosa. Cost-effectiveness impact of rivaroxaban versus new and existing prophylaxis for the prevention of venous thromboembolism after total hip or knee replacement surgery in france, italy and spain. *Thrombosis and Haemostasis*, 110(5):987–994, 2013.

[303] S. Montgomery, J. Bourget-Murray, D.Z. You, L. Nherera, A. Khoshbin, A. Atrey, and J.N. Powell. Cost-effectiveness of dual-mobility components in patients with displaced femoral neck fractures. *Bone and Joint Journal*, 103 B(12):1783–1790, 2021.

[304] N. Moretto, T.A. Comans, A.T. Chang, S.P. O'Leary, S. Osborne, H.E. Carter, D. Smith, T. Cavanagh, D. Blond, and M. Raymer. Implementation of simulation modelling to improve service planning in specialist orthopaedic and neurosurgical outpatient services. *Implementation Science*, 14(1), 2019.

[305] T. Mori, C.J. Crandall, T. Fujii, and D.A. Ganz. Cost-effectiveness of zoledronic acid compared with sequential denosumab/alendronate for older osteoporotic women in japan. *Archives of Osteoporosis*, 16(1), 2021.

[306] T. Mori, C.J. Crandall, and D.A. Ganz. Cost-effectiveness of denosumab versus

oral alendronate for elderly osteoporotic women in japan. *Osteoporosis International*, 28(5):1733–1744, 2017.

[307] W.E. Moschetti, J.F. Konopka, H.E. Rubash, and J.W. Genuario. Can robot-assisted unicompartmental knee arthroplasty be cost-effective? a markov decision analysis. *Journal of Arthroplasty*, 31(4):759–765, 2016.

[308] R. Mostafavi Tabatabaee, M.R. Rasouli, M.G. Maltenfort, and J. Parvizi. Cost-effective prophylaxis against venous thromboembolism after total joint arthroplasty: Warfarin versus aspirin. *Journal of Arthroplasty*, 30(2):159–164, 2015.

[309] M.A. Moverman, M.J. Bruha, N.R. Pagani, R.N. Puzzitiello, M.E. Menendez, and C.L. Barnes. Perioperative medical optimization of symptomatic benign prostatic hyperplasia is an economically justified infection prevention strategy in total joint arthroplasty. *Journal of Arthroplasty*, 36(7):2551–2557, 2021.

[310] R.E. Mujica-Mota, L.K. Watson, R. Tarricone, and M. Jäger. Cost-effectiveness of timely versus delayed primary total hip replacement in germany: A social health insurance perspective. *Orthopedic Reviews*, 9(3):76–87, 2017.

[311] C.D. Murawski, M.S. Jamal, E.T. Hurley, R. Buda, K. Hunt, G. McCollum, J. Paul, F. Vannini, M. Walther, Y. Yasui, Z. Ali, J.N. Altink, J. Batista, S. Bayer, G.C. Berlet, J.D.F. Calder, J. Dahmen, M.S. Davey, P. D'Hooghe, C.W. DiGiovanni, R.D. Ferkel, A.L. Gianakos, E. Giza, M. Glazebrook, L. Hangody, D. Haverkamp, B. Hintermann, Y. Hua, D.J. Hurley, J. Karlsson, S. Kearns, J.G. Kennedy, G.M.M.J. Kerkhoffs, K. Lambers, J.W. Lee, N.P. Mercer, C. Mulvin, J.A. Nunley, C. Pearce, H. Pereira, M. Prado, S.M. Raikin, I. Savage-Elliott, L.C. Schon, Y. Shimozono, J.W. Stone, S.A.S. Stufkens, M. Sullivan, M. Takao, H. Thermann, D. Thordarson, J. Toale, V. Valderrabano, C.J.A. van Bergen, C.N. van Dijk, R.J. Walls, A.S. Younger, and M.V. Hogan. Terminology for osteochondral lesions of the ankle: proceedings of the international consensus meeting on cartilage repair of the ankle. *Journal of ISAKOS*, pages 62–66, 2022.

[312] R.E. Mújica Mota. Cost-effectiveness analysis of early versus late total hip replacement in italy. *Value in Health*, 16(2):267–279, 2013.

[313] A.T. Namin, M.S. Jalali, V. Vahdat, H.S. Bedair, M.I. O'Connor, S. Kamarthi, and J.A. Isaacs. Adoption of new medical technologies: The case of customized individually made knee implants. *Value in Health*, 22(4):423–430, 2019.

[314] J. Neumann, C. Angrick, C. Höhn, D. Zajonz, M. Ghanem, A. Roth, and T. Neumuth. Surgical workflow simulation for the design and assessment of operating room setups in orthopedic surgery. *BMC Medical Informatics and Decision Making*, 20(1), 2020.

[315] J. Neumann, C. Angrick, D. Rollenhagen, A. Roth, and T. Neumuth. Perioperative workflow simulation and optimization in orthopedic surgery. *Lecture Notes in Computer Science (including subseries Lecture Notes in Artificial Intelligence and Lecture Notes in Bioinformatics)*, 11041 LNCS:3–11, 2018.

[316] L.M. Nherera, P. Trueman, A. Horner, A.J. Johnstone, T.J. Watson, and F.A. Fatoye. Comparing the costs and outcomes of an integrated twin compression screw (itcs) nail with standard of care using a single lag screw or a single helical blade cephalomedullary nail in patients with intertrochanteric hip fractures. *Journal of Orthopaedic Surgery and Research*, 13(1), 2018.

[317] L.M. Nherera, S. Verma, P. Trueman, and S. Jennings. Early economic evaluation demonstrates that noncomputerized tomography robotic-assisted surgery is cost-effective in patients undergoing unicompartmental knee arthroplasty at high-volume orthopaedic centres. *Advances in Orthopedics*, 2020, 2020.

[318] L.E. Nikkel, L. Tran, J.M. Jennings, and C.S. Hollenbeak. Cost-effectiveness of

preoperative spinal imaging before total hip arthroplasty. *Journal of Arthroplasty*, 37(1):3–9.e1, 2022. cited By 0.

[319] E.J. Novak, M.D. Silverstein, and K.J. Bozic. The cost-effectiveness of computer-assisted navigation in total knee arthroplasty. *Journal of Bone and Joint Surgery - Series A*, 89(11):2389–2397, 2007.

[320] B.U. Nwachukwu, A.S. McLawhorn, M.S. Simon, K.S. Hamid, C.A. Demetracopoulos, J.T. Deland, and S.J. Ellis. Management of end-stage ankle arthritis: Cost-utility analysis using direct and indirect costs. *Journal of Bone and Joint Surgery - American Volume*, 97(14):1159–1172, 2015.

[321] B.U. Nwachukwu, W.W. Schairer, F. McCormick, D.M. Dines, E.V. Craig, and L.V. Gulotta. Arthroplasty for the surgical management of complex proximal humerus fractures in the elderly: A cost-utility analysis. *Journal of Shoulder and Elbow Surgery*, 25(5):704–713, 2016.

[322] B.U. Nwachukwu, C. So, W.W. Schairer, B.E. Shubin-Stein, S.M. Strickland, D.W. Green, and E.R. Dodwell. Economic decision model for first-time traumatic patellar dislocations in adolescents. *American Journal of Sports Medicine*, 45(10):2267–2275, 2017.

[323] S.M. Odum, J.L. Troyer, M.P. Kelly, R.D. Dedini, and K.J. Bozic. A cost-utility analysis comparing the cost-effectiveness of simultaneous and staged bilateral total knee arthroplasty. *Journal of Bone and Joint Surgery - Series A*, 95(16):1441–1449, 2013.

[324] G. Olafsson, E. Jonsson, P. Fritzell, O. Hägg, and F. Borgström. A health economic lifetime treatment pathway model for low back pain in sweden. *Journal of Medical Economics*, 20(12):1281–1289, 2017.

[325] D.A. Ollendorf, J.G. Kotsanos, W.J. Wishner, M. Friedman, T. Cooper, M. Bittoni, and G. Osterf. Potential economic benefits of lower-extremity amputation prevention strategies in diabetes. *Diabetes Care*, 21(8):1240–1245, 1998.

[326] S. O'Neill, N. Kreif, M. Sutton, and R. Grieve. A comparison of methods for health policy evaluation with controlled pre-post designs. *Health Services Research*, 55(2):328–338, 2020.

[327] G. Osterhoff, N.N. O'Hara, J. D'Cruz, S.A. Sprague, N. Bansback, N. Evaniew, and G.P. Slobogean. A cost-effectiveness analysis of reverse total shoulder arthroplasty versus hemiarthroplasty for the management of complex proximal humeral fractures in the elderly. *Value in Health*, 20(3):404–411, 2017.

[328] M.E. Otto, C. Senter, R. Gonzales, and N. Gleason. Referring wisely: Orthopedic referral guidelines at an academic institution. *American Journal of Managed Care*, 22(5):e185–e191, 2016.

[329] S.C. Overley, S.J. McAnany, R.L. Brochin, J.S. Kim, R.K. Merrill, and S.A. Qureshi. The 5-year cost-effectiveness of two-level anterior cervical discectomy and fusion or cervical disc replacement: a markov analysis. *Spine Journal*, 18(1):63–71, 2018.

[330] A. Ozen, Y. Marmor, T. Rohleder, H. Balasubramanian, J. Huddleston, and P. Huddleston. Optimization and simulation of orthopedic spine surgery cases at mayo clinic. *Manufacturing and Service Operations Management*, 18(1):157–175, 2016.

[331] A.J. Palmer, S. Roze, W.J. Valentine, M.E. Minshall, V. Foos, F.M. Lurati, M. Lammert, and G.A. Spinas. The core diabetes model: Projecting long-term clinical outcomes, costs and cost-effectiveness of interventions in diabetes mellitus (types 1 and 2) to support clinical and reimbursement decision-making. *Current Medical Research and Opinion*, 20(SUPPL. 1):S5–S26, 2004.

[332] A.R. Paoli, H.T. Gold, S.A. Mahure, D.H. Mai, C.A. Agten, A.S. Rokito, and M.S. Virk. Treatment for symptomatic slap tears in middle-aged patients comparing repair, biceps tenodesis, and nonoperative approaches: A cost-effectiveness analysis. *Arthroscopy - Journal of Arthroscopic and Related Surgery*, 34(7):2019–2029, 2018.

[333] G. Papadopoulos, S. Griffin, H. Rathi, A. Gupta, B. Sharma, and D. van Bavel. Cost-effectiveness analysis of arthroscopic injection of a bioadhesive hydrogel implant in conjunction with microfracture for the treatment of focal chondral defects of the knee–an australian perspective. *Journal of Medical Economics*, 25(1):712–721, 2022.

[334] T.J. Parisi, J.F. Konopka, and H.S. Bedair. What is the long-term economic societal effect of periprosthetic infections after tha? a markov analysis. *Clinical Orthopaedics and Related Research*, 475(7):1891–1900, 2017.

[335] M.S. Park, C.Y. Chung, D.G. Kwon, K.H. Sung, I.H. Choi, and K.M. Lee. Prophylactic femoral varization osteotomy for contralateral stable hips in non-ambulant individuals with cerebral palsy undergoing hip surgery: Decision analysis. *Developmental Medicine and Child Neurology*, 54(3):231–239, 2012.

[336] S.L. Parker, L.H. Anderson, T. Nelson, and V.V. Patel. Cost-effectiveness of three treatment strategies for lumbar spinal stenosis: Conservative care, laminectomy, and the superion interspinous spacer. *International Journal of Spine Surgery*, 9, 2015.

[337] A. Parthan, M. Kruse, I. Agodoa, S. Silverman, and E. Orwoll. Denosumab: A cost-effective alternative for older men with osteoporosis from a swedish payer perspective. *Bone*, 59:105–113, 2014.

[338] P.G. Passias, A.E. Brown, H. Alas, C.A. Bortz, K.E. Pierce, H. Hassanzadeh, L.A. Labaran, V. Puvanesarajah, D. Vasquez-Montes, E. Wang, R.C. Ihejirika, B.G. Diebo, V. Lafage, R. Lafage, D.M. Sciubba, M.B. Janjua, T.S. Protopsaltis, A.J. Buckland, and M.C. Gerling. A cost benefit analysis of increasing surgical technology in lumbar spine fusion. *Spine Journal*, 21(2):193–201, 2021.

[339] A.M. Pearson, A.N.A. Tosteson, K.J. Koval, M.D. McKee, R.V. Cantu, J.E. Bell, and M. Vicente. Is surgery for displaced, midshaft clavicle fractures in adults cost-effective? results based on a multicenter randomized, controlled trial. *Journal of Orthopaedic Trauma*, 24(7):426–433, 2010.

[340] G. Peersman, W. Jak, T. Vandenlangenbergh, C. Jans, P. Cartier, and P. Fennema. Cost-effectiveness of unicondylar versus total knee arthroplasty: A markov model analysis. *Knee*, 21(S1):S37–S42, 2014.

[341] M. Pennington, R. Grieve, N. Black, and J.H. Van der Meulen. Cost-effectiveness of five commonly used prosthesis brands for total knee replacement in the uk: A study using the njr dataset. *PLoS ONE*, 11(3), 2016.

[342] M.J. Persson and J.A. Persson. Analysing management policies for operating room planning using simulation. *Health Care Management Science*, 13(2):182–191, 2010.

[343] W. Petersen, D. Guenther, A.B. Imhoff, M. Herbort, T. Stein, C. Schoepp, R. Akoto, J. Höher, S. Scheffler, A. Stoehr, T. Stoffels, M. Häner, T. Hees, J. Mehl, A. Ellermann, M. Krause, N. Mengis, C. Eberle, P.E. Müller, R. Best, P.M. Lutz, and A. Achtnich. Management after acute rupture of the anterior cruciate ligament (acl). part 1: Acl reconstruction has a protective effect on secondary meniscus and cartilage lesions. *Knee Surgery, Sports Traumatology, Arthroscopy*, 2022.

[344] F.M. Pieracci, S. Majercik, F. Ali-Osman, D. Ang, A. Doben, J.G. Edwards,

B. French, M. Gasparri, S. Marasco, C. Minshall, B. Sarani, W. Tisol, D.H. VanBoerum, and T.W. White. Consensus statement: Surgical stabilization of rib fractures rib fracture colloquium clinical practice guidelines. *Injury*, 48(2):307–321, 2017.

[345] B. Pijls, O. Dekkers, S. Middeldorp, E. Valstar, H. Van Der Heide, H. Van Der Linden-Van Der Zwaag, and R. Nelissen. Aquila: Assessment of quality in lower limb arthroplasty. an expert delphi consensus for total knee and total hip arthroplasty. *BMC Musculoskeletal Disorders*, 12, 2011.

[346] U. Plenge, M.B. Nortje, L.C. Marais, J.D. Jordaan, R. Parker, N. Van Der Westhuizen, J.F. Van Der Merwe, J. Marais, W.V. September, G.L. Davies, T. Pretorius, C. Solomon, P. Ryan, A.M. Torborg, Z. Farina, R. Smit, C. Cairns, H. Shanahan, S. Sombili, A. Mazibuko, H.R. Hobbs, O.S. Porrill, N.E. Timothy, R.E. Siebritz, C. Van Der Westhuizen, A.J. Troskie, C.A. Blake, L.A. Gray, T.W. Munting, H.K.S. Steinhaus, P. Rowe, J.G. Van Der Walt, R. Isaacs Noordien, A. Theron, and B.M. Biccard. Optimising perioperative care for hip and knee arthroplasty in south africa: A delphi consensus study. *BMC Musculoskeletal Disorders*, 19(1), 2018.

[347] K.E. Ponnusamy, E.M. Vasarhelyi, R.W. McCalden, L.E. Somerville, and J.D. Marsh. Cost-effectiveness of total hip arthroplasty versus nonoperative management in normal, overweight, obese, severely obese, morbidly obese, and super obese patients: A markov model. *Journal of Arthroplasty*, 33(12):3629–3636, 2018.

[348] K.E. Ponnusamy, E.M. Vasarhelyi, L. Somerville, R.W. McCalden, and J.D. Marsh. Cost-effectiveness of total knee arthroplasty vs nonoperative management in normal, overweight, obese, severely obese, morbidly obese, and super-obese patients: A markov model. *Journal of Arthroplasty*, 33(7):S32–S38, 2018.

[349] D.Y. Ponzio, A.G. Park, S.B. Bhat, and J.J. Purtill. Can we reduce the utilization of home-visiting nurse services after primary total joint arthroplasty? *Journal of Arthroplasty*, 31(9):50–53, 2016.

[350] A. Premkumar, D.G. Lebrun, S. Sidharthan, C.L. Penny, E.R. Dodwell, A.S. McLawhorn, and B.U. Nwachukwu. Bariatric surgery prior to total hip arthroplasty is cost-effective in morbidly obese patients. *Journal of Arthroplasty*, 35(7):1766–1775.e3, 2020.

[351] R. Pulikottil-Jacob, M. Connock, N.-B. Kandala, H. Mistry, A. Grove, K. Freeman, M. Costa, P. Sutcliffe, and A. Clarke. Cost effectiveness of total hip arthroplasty in osteoarthritis: Comparison of devices with differing bearing surfaces and modes of fixation. *Bone and Joint Journal*, 97-B(4):449–457, 2015.

[352] R. Pulikottil-Jacob, M. Connock, N.-B. Kandala, H. Mistry, A. Grove, K. Freeman, M. Costa, P. Sutcliffe, and A. Clarke. Has metal-on-metal resurfacing been a cost-effective intervention for health care providers? - a registry based study. *PLoS ONE*, 11(11), 2016.

[353] R. Quigley, N. Verma, Jr. Evuarherhe, A., and B.J. Cole. Rotator cuff repair with graft augmentation improves function, decreases revisions, and is cost-effective. *Arthroscopy - Journal of Arthroscopic and Related Surgery*, 38(7):2166–2174, 2022.

[354] S.A. Qureshi, S. McAnany, V. Goz, S.M. Koehler, and A.C. Hecht. Cost-effectiveness analysis: Comparing single-level cervical disc replacement and single-level anterior cervical discectomy and fusion: Clinical article. *Journal of Neurosurgery: Spine*, 19(5):546–554, 2013.

[355] M. Raad, C. Ortiz-Babilonia, H. Hassanzadeh, V. Puvanesarajah, K. Kebaish,

and A. Jain. Cost-utility analysis of neoadjuvant teriparatide therapy in osteopenic patients undergoing adult spinal deformity surgery. *Spine*, 47(16):1121–1127, 2022.

[356] P.V. Rajan, M.K. Ng, A. Klika, A.F. Kamath, G.F. Muschler, C.A. Higuera, and N.S. Piuzzi. The cost-effectiveness of platelet-rich plasma injections for knee osteoarthritis: A markov decision analysis. *Journal of Bone and Joint Surgery*, 102(18):E104, 2020.

[357] P.V. Rajan, R.A. Qudsi, G.S.M. Dyer, and E. Losina. The cost-effectiveness of surgical fixation of distal radial fractures. *Journal of Bone and Joint Surgery - American Volume*, 100(3):e13, 2018.

[358] A.J. Ramme, E.J. Strauss, L. Jazrawi, and H.T. Gold. Cost effectiveness of meniscal allograft for torn discoid lateral meniscus in young women. *Physician and Sportsmedicine*, 44(3):278–282, 2016.

[359] D.C. Ramsey and D.M. Friess. Cost-effectiveness analysis of syndesmotic screw versus suture button fixation in tibiofibular syndesmotic injuries. *Journal of Orthopaedic Trauma*, 32(6):e198–e203, 2018.

[360] C.-L. Rau, P.-F.J. Tsai, S.-F.M. Liang, J.-C. Tan, H.-C. Syu, Y.-L. Jheng, T.-S. Ciou, and F.-S. Jaw. Using discrete-event simulation in strategic capacity planning for an outpatient physical therapy service. *Health Care Management Science*, 16(4):352–365, 2013.

[361] A.J. Recker, T.L. Waters, G. Bullock, S. Rosas, II Scholten, D.J., K. Nicholson, and B.R. Waterman. Biceps tenodesis has greater expected value than repair for isolated type ii slap tears: A meta-analysis and expected-value decision analysis. *Arthroscopy - Journal of Arthroscopic and Related Surgery*, 2022.

[362] W.K. Redekop, J. McDonnell, P. Verboom, K. Lovas, and Z. Kalo. The cost effectiveness of apligraf® treatment of diabetic foot ulcers. *PharmacoEconomics*, 21(16):1171–1183, 2003.

[363] M.L. Reilingh, C.D. Murawski, C.W. DiGiovanni, J. Dahmen, P.N.F. Ferrao, K.T.A. Lambers, J.S. Ling, Y. Tanaka, G.M.M.J. Kerkhoffs, J. Ackermann, Jr. Adams, S.B., C.L. Andrews, C. Angthong, J.P. Batista, O.L. Baur, S. Bayer, C. Becher, G.C. Berlet, L.A.T. Boakye, A.J. Brown, R. Buda, J.D.F. Calder, G.L. Canata, D.S. Carreira, T.O. Clanton, P. D'Hooghe, M.E. Dombrowski, M.C. Drakos, R.D. Ferkel, L.A. Fortier, M. Glazebrook, E. Giza, M. Gomaa, S. Görtz, A.M. Haleem, K. Hamid, L. Hangody, C.P. Hannon, D. Haverkamp, J. Hertel, B. Hintermann, M.V. Hogan, K.J. Hunt, E.T. Hurley, J. Karlsson, S.R. Kearns, J.G. Kennedy, H.J. Kim, S.W. Kong, S.A. Labib, J.W. Lee, K.B. Lee, U.G. Longo, A. Marangon, G. McCollum, A.W. Mitchell, P.N. Mittwede, S. Nehrer, P. Niemeyer, II Nunley, J.A., M.J. O'Malley, D.O. Osei-Hwedieh, J. Paul, C.J. Pearce, H. Pereira, A. Popchak, M.P. Prado, S.M. Raikin, B.B. Rothrauff, L.C. Schon, Y. Shimozono, H. Simpson, N.A. Smyth, C.M. Sofka, P. Spennacchio, J.W. Stone, M. Sullivan, M. Takao, D.B. Thordarson, R. Tuan, V. Valderrabano, C.J.A. van Bergen, C.N. van Dijk, P.A.D. van Dijk, F. Vannini, T. Vaseenon, M. Walther, M. Wiewiorski, X. Xu, Y. Yasui, H. Yinghui, I. Yoshimura, A.S.E. Younger, Z. Zhang, and the International Consensus Group on Cartilage Repair of the Ankle. Fixation techniques: Proceedings of the international consensus meeting on cartilage repair of the ankle. *Foot and Ankle International*, 39(1_suppl):23S–27S, 2018.

[364] N. Revankar, J. Patterson, A. Kadambi, V. Raymond, and W. El-Hadi. A canadian study of the cost-effectiveness of apixaban compared with enoxaparin for post-surgical venous thromboembolism prevention. *Postgraduate medicine*,

125(4):141–153, 2013.

[365] K.F. Rezende, M.B. Ferraz, D.A. Malerbi, N.H. Melo, M.P. Nunes, H.C. Pedrosa, and A.R. Chacra. Predicted annual costs for inpatients with diabetes and foot ulcers in a developing country - a simulation of the current situation in brazil. *Diabetic Medicine*, 27(1):109–112, 2010.

[366] R.S. Rice, B.R. Waterman, and J.H. Lubowitz. Allograft versus autograft decision for anterior cruciate ligament reconstruction: An expected-value decision analysis evaluating hypothetical patients. *Arthroscopy - Journal of Arthroscopic and Related Surgery*, 28(4):539–547, 2012.

[367] J.A. Rihn, S. Bhat, J. Grauer, J. Harrop, Z. Ghogawala, A.R. Vaccaro, and A.S. Hilibrand. Economic and outcomes analysis of recalcitrant cervical radiculopathy: Is nonsurgical management or surgery more cost-effective? *Journal of the American Academy of Orthopaedic Surgeons*, 27(14):533–540, 2019.

[368] R. Robinson, T.C. Wirt, C. Barbosa, A. Amidi, S. Chen, R.M. Joseph, and A.E. Fleischer. Routine use of low-molecular-weight heparin for deep venous thrombosis prophylaxis after foot and ankle surgery: A cost-effectiveness analysis. *Journal of Foot and Ankle Surgery*, 57(3):543–551, 2018.

[369] M. Rogers, S. Dart, S. Odum, and J. Fleischli. A cost-effectiveness analysis of isolated meniscal repair versus partial meniscectomy for red-red zone, vertical meniscal tears in the young adult. *Arthroscopy - Journal of Arthroscopic and Related Surgery*, 35(12):3280–3286, 2019.

[370] T.R. Rohleder, P. Lewkonia, D.P. Bischak, P. Duffy, and R. Hendijani. Using simulation modeling to improve patient flow at an outpatient orthopedic clinic. *Health Care Management Science*, 14(2):135–145, 2011.

[371] J.J. Rongen, T.M. Govers, P. Buma, J.P.C. Grutters, and G. Hannink. Societal and economic effect of meniscus scaffold procedures for irreparable meniscus injuries. *American Journal of Sports Medicine*, 44(7):1724–1734, 2016.

[372] J.J. Rongen, T.M. Govers, P. Buma, M.M. Rovers, and G. Hannink. Arthroscopic meniscectomy for degenerative meniscal tears reduces knee pain but is not cost-effective in a routine health care setting: a multi-center longitudinal observational study using data from the osteoarthritis initiative. *Osteoarthritis and Cartilage*, 26(2):184–194, 2018.

[373] A. Roposch, J.H. Wedge, and M.D. Krahn. The role of the ossific nucleus in the treatment of established hip dislocation. *Clinical Orthopaedics and Related Research*, 449:295–302, 2006.

[374] B.B. Rothrauff, C.D. Murawski, C. Angthong, C. Becher, S. Nehrer, P. Niemeyer, M. Sullivan, V. Valderrabano, M. Walther, R.D. Ferkel, J. Ackermann, Jr. Adams, S.B., C.L. Andrews, J.P. Batista, O.L. Baur, S. Bayer, G.C. Berlet, L.A.T. Boakye, A.J. Brown, R. Buda, J.D.F. Calder, G.L. Canata, D.S. Carreira, T.O. Clanton, J. Dahmen, P. D'Hooghe, C.W. DiGiovanni, M.E. Dombrowski, M.C. Drakos, P.N.F. Ferrao, L.A. Fortier, M. Glazebrook, E. Giza, M. Gomaa, S. Görtz, A.M. Haleem, K. Hamid, L. Hangody, C.P. Hannon, D. Haverkamp, J. Hertel, B. Hintermann, M.V. Hogan, K.J. Hunt, E.T. Hurley, J. Karlsson, S.R. Kearns, J.G. Kennedy, G.M.M.J. Kerkhoffs, H.J. Kim, S.W. Kong, S.A. Labib, K.T.A. Lambers, J.W. Lee, K.B. Lee, J.S. Ling, U.G. Longo, A. Marangon, G. McCollum, A.W. Mitchell, P.N. Mittwede, II Nunley, J.A., M.J. O'Malley, D.O. Osei-Hwedieh, J. Paul, C.J. Pearce, H. Pereira, A. Popchak, M.P. Prado, S.M. Raikin, M.L. Reilingh, L.C. Schon, Y. Shimozono, H. Simpson, N.A. Smyth, C.M. Sofka, P. Spennacchio, J.W. Stone, M. Takao, Y. Tanaka, D.B. Thordarson, R. Tuan, C.J.A. van Bergen, C.N. van Dijk, P.A.D. van Dijk, F. Vannini,

T. Vaseenon, M. Wiewiorski, X. Xu, Y. Yasui, H. Yinghui, I. Yoshimura, A.S.E. Younger, Z. Zhang, and the International Consensus Group on Cartilage Repair of the Ankle. Scaffold-based therapies: Proceedings of the international consensus meeting on cartilage repair of the ankle. *Foot and Ankle International*, 39(1_suppl):41S–47S, 2018.

[375] D. Ruiz Jr., L. Koenig, T.M. Dall, P. Gallo, A. Narzikul, J. Parvizi, and J. Tongue. The direct and indirect costs to society of treatment for end-stage knee osteoarthritis. *Journal of Bone and Joint Surgery - Series A*, 95(16):1473–1480, 2013.

[376] L. Ryttberg, A. Diamantopoulos, F. Forster, M. Lees, A. Fraschke, and I. Bjrholt. Cost-effectiveness of rivaroxaban versus heparins for prevention of venous thromboembolism after total hip or knee surgery in sweden. *Expert Review of Pharmacoeconomics and Outcomes Research*, 11(5):601–615, 2011.

[377] H. Saadouli, B. Jerbi, A. Dammak, L. Masmoudi, and A. Bouaziz. A stochastic optimization and simulation approach for scheduling operating rooms and recovery beds in an orthopedic surgery department. *Computers and Industrial Engineering*, 80:72–79, 2015.

[378] K.J. Saleh, K.C. Wood, A. Gafni, and A.E. Gross. Immediate surgery versus waiting list policy in revision total hip arthroplasty: An economic evaluation. *Journal of Arthroplasty*, 12(1):1–10, 1997.

[379] L.T. Samuel, A.A. Sultan, M. Kheir, J. Villa, P. Patel, J. Parvizi, and C.A. Higuera. Positive alpha-defensin at reimplantation of a two-stage revision arthroplasty is not associated with infection at 1 year. *Clinical Orthopaedics and Related Research*, 477(7):1615–1621, 2019.

[380] E.M. Samuelson, J.A. Ebel, S.B. Reynolds, R.M. Arnold, and D.E. Brown. The cost-effectiveness of platelet-rich plasma compared with hyaluronic acid injections for the treatment of knee osteoarthritis. *Arthroscopy - Journal of Arthroscopic and Related Surgery*, 36(12):3072–3078, 2020.

[381] E.M. Samuelson, S.M. Odum, and J.E. Fleischli. The cost-effectiveness of using platelet-rich plasma during rotator cuff repair: A markov model analysis. *Arthroscopy - Journal of Arthroscopic and Related Surgery*, 32(7):1237–1244, 2016.

[382] Z.M. Sardar, J.R. Coury, M. Cerpa, C.J. Dewald, C.P. Ames, C. Shuhart, C. Watkins, D.W. Polly, D.R. Dirschl, E.O. Klineberg, J.R. Dimar, K.D. Krohn, K.M. Kebaish, L.L. Tosi, M. Kelly, N.E. Lane, N.C. Binkley, S.H. Berven, N.J. Lee, P. Anderson, P.D. Angevine, R.A. Lehman, and L.G. Lenke. Best practice guidelines for assessment and management of osteoporosis in adult patients undergoing elective spinal reconstruction. *Spine*, 47(2):128–135, 2022.

[383] J.K. Schmier, M. Halevi, G. Maislin, and K. Ong. Comparative cost effectiveness of coflex® interlaminar stabilization versus instrumented posterolateral lumbar fusion for the treatment of lumbar spinal stenosis and spondylolisthesis. *ClinicoEconomics and Outcomes Research*, 6(1):125–131, 2014.

[384] W.R. Schultz, J.N. Weinstein, S.L. Weinstein, and B.G. Smith. Prophylactic pinning of the contralateral hip in slipped capital femoral epiphysis: Evaluation of long-term outcome for the contralateral hip with use of decision analysis. *Journal of Bone and Joint Surgery*, 84(8):1305–1314, 2002.

[385] A. Schumaier, D. Kovacevic, C. Schmidt, A. Green, A. Rokito, C. Jobin, E. Yian, F. Cuomo, J. Koh, M. Gilotra, M. Ramirez, M. Williams, R. Burks, R. Stanley, S. Hasan, S. Paxton, S. Hasan, W. Nottage, W. Levine, U. Srikumaran, and B. Grawe. Defining massive rotator cuff tears: a delphi consensus study. *Journal*

of Shoulder and Elbow Surgery, 29(4):674–680, 2020.

[386] D. Schwicker. Cost effectiveness of lumbar disc surgery and of a preventive treatment for peridural fibrosis. *European Spine Journal*, 5(SUPPL. 1):S21–S25, 1996. cited By 12.

[387] D.J. Scott, S. Sherman, A. Dhawan, B.J. Cole, Jr. Bach, B.R., and III Mather, R.C. Quantifying the economic impact of provider volume through adverse events: The case of sports medicine. *Orthopaedic Journal of Sports Medicine*, 3(3), 2015.

[388] K. Seng, D. Appleby, and J.H. Lubowitz. Operative versus nonoperative treatment of anterior cruciate ligament rupture in patients aged 40 years or older: An expected-value decision analysis. *Arthroscopy - Journal of Arthroscopic and Related Surgery*, 24(8):914–920, 2008.

[389] III Serino, J., III Burnett, R.A., A.J. Boniello, J. Yang, S.M. Sporer, and C.J. Della Valle. The cost-effectiveness of tibial metaphyseal cones in revision total knee arthroplasty. *Journal of Arthroplasty*, 37(6):S50–S55, 2022.

[390] E. Sharifi, H. Sharifi, S. Morshed, K. Bozic, and M. Diab. Cost-effectiveness analysis of periacetabular osteotomy. *Journal of Bone and Joint Surgery - Series A*, 90(7):1447–1456, 2008.

[391] M.J. Shauver, P.J. Clapham, and K.C. Chung. An economic analysis of outcomes and complications of treating distal radius fractures in the elderly. *Journal of Hand Surgery*, 36(12):1912–1918.e3, 2011.

[392] K.A. Shaw, V. Heboyan, N.D. Fletcher, and J.S. Murphy. Comparative cost–utility analysis of postoperative discharge pathways following posterior spinal fusion for scoliosis in non-ambulatory cerebral palsy patients. *Spine Deformity*, 9(6):1659–1667, 2021.

[393] D.W. Shearer, J. Kramer, K.J. Bozic, and B.T. Feeley. Is hip arthroscopy cost-effective for femoroacetabular impingement? *Clinical Orthopaedics and Related Research*, 470(4):1079–1089, 2012.

[394] D.W. Shearer, J. Youm, and K.J. Bozic. Short-term complications have more effect on cost-effectiveness of tha than implant longevity. *Clinical Orthopaedics and Related Research*, 473(5):1702–1708, 2015.

[395] Y. Shimozono, A.J. Brown, J.P. Batista, C.D. Murawski, M. Gomaa, S.W. Kong, T. Vaseenon, M. Takao, M. Glazebrook, J. Ackermann, Jr. Adams, S.B., C.L. Andrews, C. Angthong, O.L. Baur, S. Bayer, C. Becher, G.C. Berlet, L.A.T. Boakye, R. Buda, J.D.F. Calder, G.L. Canata, D.S. Carreira, T.O. Clanton, J. Dahmen, P. D'Hooghe, C.W. DiGiovanni, M.E. Dombrowski, M.C. Drakos, R.D. Ferkel, P.N.F. Ferrao, L.A. Fortier, E. Giza, S. Görtz, A.M. Haleem, K. Hamid, L. Hangody, C.P. Hannon, D. Haverkamp, J. Hertel, B. Hintermann, M.V. Hogan, K.J. Hunt, E.T. Hurley, J. Karlsson, S.R. Kearns, J.G. Kennedy, G.M.M.J. Kerkhoffs, H.J. Kim, S.A. Labib, K.T.A. Lambers, J.W. Lee, K.B. Lee, J.S. Ling, U.G. Longo, A. Marangon, G. McCollum, A.W. Mitchell, P.N. Mittwede, S. Nehrer, P. Niemeyer, II Nunley, J.A., M.J. O'Malley, D.O. Osei-Hwedieh, J. Paul, C.J. Pearce, H. Pereira, A. Popchak, M.P. Prado, S.M. Raikin, M.L. Reilingh, B.B. Rothrauff, L.C. Schon, H. Simpson, N.A. Smyth, C.M. Sofka, P. Spennacchio, J.W. Stone, M. Sullivan, Y. Tanaka, D.B. Thordarson, R. Tuan, V. Valderrabano, C.J.A. van Bergen, C.N. van Dijk, P.A.D. van Dijk, F. Vannini, M. Walther, M. Wiewiorski, X. Xu, Y. Yasui, H. Yinghui, I. Yoshimura, A.S.E. Younger, Z. Zhang, and the International Consensus Group on Cartilage Repair of the Ankle. Subchondral pathology: Proceedings of the international consensus meeting on cartilage repair of the ankle. *Foot and Ankle International*,

39(1_suppl):48S–53S, 2018.

[396] L. Si, T.M. Winzenberg, Q. Jiang, and A.J. Palmer. Screening for and treatment of osteoporosis: construction and validation of a state-transition microsimulation cost-effectiveness model. *Osteoporosis International*, 26(5):1477–1489, 2015.

[397] S. Silverman, I. Agodoa, M. Kruse, A. Parthan, and E. Orwoll. Denosumab for elderly men with osteoporosis: A cost-effectiveness analysis from the us payer perspective. *Journal of Osteoporosis*, 2015, 2015.

[398] Y.W. Simwita and B.I. Helgheim. Improving surgeon utilization in an orthopedic department using simulation modeling. *Journal of Healthcare Leadership*, 8:41–50, 2016.

[399] Y.W. Simwita and B.I. Helgheim. Simulation analysis of resource flexibility on healthcare processes. *Journal of Multidisciplinary Healthcare*, 9:519–528, 2016.

[400] C.L. Siqueira, E.F. Arruda, L. Bahiense, G.L. Bahr, and G.R. Motta. Long-term integrated surgery room optimization and recovery ward planning, with a case study in the brazilian national institute of traumatology and orthopedics (into). *European Journal of Operational Research*, 264(3):870–883, 2018.

[401] D.D. Skaar, T. Park, M.F. Swiontkowski, and K.M. Kuntz. Cost-effectiveness of antibiotic prophylaxis for dental patients with prosthetic joints comparisons of antibiotic regimens for patients with total hip arthroplasty. *Journal of the American Dental Association*, 146(11):830–839, 2015.

[402] D.D. Skaar, T. Park, M.F. Swiontkowski, and K.M. Kuntz. Is antibiotic prophylaxis cost-effective for dental patients following total knee arthroplasty? *JDR Clinical and Translational Research*, 4(1):9–18, 2019.

[403] C. Skedgel, R. Goeree, S. Pleasance, K. Thompson, B. O'Brien, and D. Anderson. The cost-effectiveness of extended-duration antithrombotic prophylaxis after total hip arthroplasty. *Journal of Bone and Joint Surgery - Series A*, 89(4):819–828, 2007.

[404] G.P. Slobogean, C.A. Marra, M. Sadatsafavi, and D.W. Sanders. Is surgical fixation for stress-positive unstable ankle fractures cost effective? results of a multicenter randomized control trial. *Journal of Orthopaedic Trauma*, 26(11):652–658, 2012.

[405] G.P. Slobogean, P.J. O'Brien, and C.A. Brauer. Single-dose versus multiple-dose antibiotic prophylaxis for the surgical treatment of closed fractures. *Acta Orthopaedica*, 81(2):256–262, 2010.

[406] J. Slover, B. Espehaug, L.I. Havelin, L.B. Engesaeter, O. Furnes, I. Tomek, and A. Tosteson. Cost-effectiveness of unicompartmental and total knee arthroplasty in elderly low-demand patients: A markov decision analysis. *Journal of Bone and Joint Surgery - Series A*, 88(11):2348–2355, 2006.

[407] J. Slover, J.P. Haas, M. Quirno, M.S. Phillips, and J.A. Bosco. Cost-effectiveness of a staphylococcus aureus screening and decolonization program for high-risk orthopedic patients. *Journal of Arthroplasty*, 26(3):360–365, 2011.

[408] J. Slover, M.V. Hoffman, H. Malchau, A.N.A. Tosteson, and K.J. Koval. A cost-effectiveness analysis of the arthroplasty options for displaced femoral neck fractures in the active, healthy, elderly population. *Journal of Arthroplasty*, 24(6):854–860, 2009.

[409] J.D. Slover, K.A. Mullaly, A. Payne, R. Iorio, and J. Bosco. What is the best strategy to minimize after-care costs for total joint arthroplasty in a bundled payment environment? *Journal of Arthroplasty*, 31(12):2710–2713, 2016.

[410] J.D. Slover, H.E. Rubash, H. Malchau, and J.A. Bosco. Cost-effectiveness analysis of custom total knee cutting blocks. *Journal of Arthroplasty*, 27(2):180–185,

2012.

[411] J.D. Slover, A.N.A. Tosteson, K.J. Bozic, H.E. Rubash, and H. Malchau. Impact of hospital volume on the economic value of computer navigation for total knee replacement. *Journal of Bone and Joint Surgery - Series A*, 90(7):1492–1500, 2008.

[412] P.A. Slullitel, J.I. Oñativia, G. Zanotti, F. Comba, F. Piccaluga, and M.A. Buttaro. One-stage exchange should be avoided in periprosthetic joint infection cases with massive femoral bone loss or with history of any failed revision to treat periprosthetic joint infection. *Bone and Joint Journal*, 103-B(7):1247–1253, 2021.

[413] II Smith, W.B., J. Steinberg, S. Scholtes, and I.R. Mcnamara. Medial compartment knee osteoarthritis: age-stratified cost-effectiveness of total knee arthroplasty, unicompartmental knee arthroplasty, and high tibial osteotomy. *Knee Surgery, Sports Traumatology, Arthroscopy*, 25(3):924–933, 2017.

[414] N.A. Smyth, C.D. Murawski, Jr Adams, S.B., G.C. Berlet, R. Buda, S.A. Labib, II Nunley, J.A., S.M. Raikin, J. Ackermann, C.L. Andrews, C. Angthong, J.P. Batista, O.L. Baur, S. Bayer, C. Becher, L.A.T. Boakye, A.J. Brown, J.D.F. Calder, G.L. Canata, D.S. Carreira, T.O. Clanton, J. Dahmen, P. D'Hooghe, C.W. DiGiovanni, M.E. Dombrowski, M.C. Drakos, R.D. Ferkel, P.N.F. Ferrao, L.A. Fortier, M. Glazebrook, E. Giza, M. Gomaa, S. Görtz, A.M. Haleem, K. Hamid, L. Hangody, C.P. Hannon, D. Haverkamp, J. Hertel, B. Hintermann, M.V. Hogan, K.J. Hunt, E.T. Hurley, J. Karlsson, S.R. Kearns, J.G. Kennedy, G.M.M.J. Kerkhoffs, H.J. Kim, S.W. Kong, K.T.A. Lambers, J.W. Lee, K.B. Lee, J.S. Ling, U.G. Longo, A. Marangon, G. McCollum, A.W. Mitchell, P.N. Mittwede, S. Nehrer, P. Niemeyer, M.J. O'Malley, D.O. Osei-Hwedieh, J. Paul, C.J. Pearce, H. Pereira, A. Popchak, M.P. Prado, M.L. Reilingh, B.B. Rothrauff, L.C. Schon, Y. Shimozono, H. Simpson, C.M. Sofka, P. Spennacchio, J.W. Stone, M. Sullivan, M. Takao, Y. Tanaka, D.B. Thordarson, R. Tuan, V. Valderrabano, C.J.A. van Bergen, C.N. van Dijk, P.A.D. van Dijk, F. Vannini, T. Vaseenon, M. Walther, M. Wiewiorski, X. Xu, Y. Yasui, H. Yinghui, I. Yoshimura, A.S.E. Younger, Z. Zhang, and the International Consensus Group on Cartilage Repair of the Ankle. Osteochondral allograft: Proceedings of the international consensus meeting on cartilage repair of the ankle. *Foot and Ankle International*, 39(1_suppl):35S–40S, 2018.

[415] N.F. SooHoo and G. Kominski. Cost-effectiveness analysis of total ankle arthroplasty. *Journal of Bone and Joint Surgery - Series A*, 86(11):2446–2455, 2004.

[416] N.F. Soohoo, H. Sharifi, G. Kominski, and J.R. Liebeuman. Cost-effectiveness analysis of unicompartmental knee arthroplasty as an alternative to total knee arthroplasty for unicompartmental osteoarthritis. *Journal of Bone and Joint Surgery - Series A*, 88(9):1975–1982, 2006.

[417] N.F. SooHoo, S. Vyas, J. Manunga, H. Sharifi, G. Kominski, and J.R. Lieberman. Cost-effectiveness analysis of core decompression. *Journal of Arthroplasty*, 21(5):670–681, 2006.

[418] D.J. Spiegelhalter and N.G. Best. Bayesian approaches to multiple sources of evidence and uncertainty in complex cost-effectiveness modelling. *Statistics in Medicine*, 22(23):3687–3709, 2003.

[419] U.J. Spiegl, S.C. Faucett, M.P. Horan, R.J. Warth, and P.J. Millett. The role of arthroscopy in the management of glenohumeral osteoarthritis: A markov decision model. *Arthroscopy - Journal of Arthroscopic and Related Surgery*, 30(11):1392–1399, 2014.

[420] K. Srivastava, K.J. Bozic, C. Silverton, A.J. Nelson, E.C. Makhni, and J.J. Davis. Reconsidering strategies for managing chronic periprosthetic joint infection in total knee arthroplasty: Using decision analytics to find the optimal strategy between one-stage and two-stage total knee revision. *Journal of Bone and Joint Surgery - American Volume*, 101(1):14–24, 2019.

[421] L. Standfield, T. Comans, M. Raymer, S. O'Leary, N. Moretto, and P. Scuffham. The efficiency of increasing the capacity of physiotherapy screening clinics or traditional medical services to address unmet demand in orthopaedic outpatients: A practical application of discrete event simulation with dynamic queuing. *Applied Health Economics and Health Policy*, 14(4):479–491, 2016.

[422] L.B. Standfield, T.A. Comans, and P.A. Scuffham. An empirical comparison of markov cohort modeling and discrete event simulation in a capacity-constrained health care setting. *European Journal of Health Economics*, 18(1):33–47, 2017.

[423] C. Steltzlen, J.-Y. Lazennec, Y. Catonné, and M.-A. Rousseau. Unstable odontoid fracture: Surgical strategy in a 22-case series, and literature review. *Orthopaedics and Traumatology: Surgery and Research*, 99(5):615–623, 2013.

[424] M. Stevenson, T. Gomersall, M.L. Jones, A. Rawdin, M. Hernández, S. Dias, D. Wilson, and A. Rees. Percutaneous vertebroplasty and percutaneous balloon kyphoplasty for the treatment of osteoporotic vertebral fractures: A systematic review and cost-effectiveness analysis. *Health Technology Assessment*, 18(17):1–289, 2014.

[425] B.A. Stewart, A.M. Momaya, M.D. Silverstein, and D. Lintner. The cost-effectiveness of anterior cruciate ligament reconstruction in competitive athletes. *American Journal of Sports Medicine*, 45(1):23–33, 2017.

[426] A.C. Straat, P. Coenen, D.J.M. Smit, G. Hulsegge, E.V.A. Bouwsma, J.A.F. Huirne, R.C. van Geenen, R.P.A. Janssen, T.A.E.J. Boymans, G.M.M.J. Kerkhoffs, J.R. Anema, and P.P.F.M. Kuijer. Development of a personalized m/ehealth algorithm for the resumption of activities of daily life including work and sport after total and unicompartmental knee arthroplasty: A multidisciplinary delphi study. *International Journal of Environmental Research and Public Health*, 17(14):1–15, 2020.

[427] J. Strony, S. Brown, P. Choong, M. Ghert, L. Jeys, and R.J. O'Donnell. Musculoskeletal infection in orthopaedic oncology: Assessment of the 2018 international consensus meeting on musculoskeletal infection. *Journal of Bone and Joint Surgery - American Volume*, 101(20):E107, 2019.

[428] F. Strozzi, E. Garagiola, and P. Trucco. Analysing the attractiveness, availability and accessibility of healthcare providers via social network analysis (sna). *Decision Support Systems*, 120:25–37, 2019.

[429] O. Ström, C. Leonard, D. Marsh, and C. Cooper. Cost-effectiveness of balloon kyphoplasty in patients with symptomatic vertebral compression fractures in a uk setting. *Osteoporosis International*, 21(9):1599–1608, 2010.

[430] J.D. Stull, S.B. Bhat, J.M. Kane, and S.M. Raikin. Economic burden of inpatient admission of ankle fractures. *Foot and Ankle International*, 38(9):997–1004, 2017.

[431] M.E. Suarez-Almazor, P. Kaul, C.J. Kendall, L.D. Saunders, and D.W.C. Johnston. The cost-effectiveness of magnetic resonance imaging for patients with internal derangement of the knee. *International Journal of Technology Assessment in Health Care*, 15(2):392–405, 1999.

[432] N. Suhaimi, V. Vahdat, and J. Griffin. Building a flexible simulation model for modeling multiple outpatient orthopedic clinics. volume 2018-December, pages 2612–2623. Institute of Electrical and Electronics Engineers Inc., 2019.

[433] X. Sun, X. Zhen, X. Hu, Y. Li, S.Y. Gu, Y. Gu, Z. Zhao, W. Yang, and H. Dong. Cost-utility analysis of imrecoxib compared with diclofenac for patients with osteoarthritis. *Cost Effectiveness and Resource Allocation*, 19(1), 2021.

[434] K.H. Sung, C.Y. Chung, K.M. Lee, S.Y. Lee, I.H. Choi, T.-J. Cho, W.J. Yoo, and M.S. Park. Determining the best treatment for coronal angular deformity of the knee joint in growing children: A decision analysis. *BioMed Research International*, 2014, 2014.

[435] E. Svantesson, E. Hamrin Senorski, K.E. Webster, J. Karlsson, T. Diermeier, B.B. Rothrauff, S.J. Meredith, T. Rauer, J.J. Irrgang, K.P. Spindler, C.B. Ma, V. Musahl, F.H. Fu, O.R. Ayeni, F. Della Villa, S. Della Villa, S. Dye, M. Ferretti, A. Getgood, T. Järvelä, C.C. Kaeding, R. Kuroda, B. Lesniak, R.G. Marx, G.B. Maletis, L. Pinczewski, A. Ranawat, B. Reider, R. Seil, C. van Eck, B.R. Wolf, P. Yung, S. Zaffagnini, M. Zheng, and The Panther Symposium ACLInjury Clinical Outcomes Consensus Group. Clinical outcomes after anterior cruciate ligament injury: panther symposium acl injury clinical outcomes consensus group. *Knee Surgery, Sports Traumatology, Arthroscopy*, 28(8):2415–2434, 2020.

[436] A. Svedbom, L. Alvares, C. Cooper, D. Marsh, and O. Ström. Balloon kyphoplasty compared to vertebroplasty and nonsurgical management in patients hospitalised with acute osteoporotic vertebral compression fracture: A uk cost-effectiveness analysis. *Osteoporosis International*, 24(1):355–367, 2013.

[437] E. Swart, E.C. Makhni, W. Macaulay, M.P. Rosenwasser, and K.J. Bozic. Cost-effectiveness analysis of fixation options for intertrochanteric hip fractures. *Journal of Bone and Joint Surgery - American Volume*, 96(19):1612–1620, 2014.

[438] E. Swart, L. Redler, P.D. Fabricant, B.R. Mandelbaum, C.S. Ahmad, and Y.C. Wang. Prevention and screening programs for anterior cruciate ligament injuries in young athletes: A cost-effectiveness analysis. *Journal of Bone and Joint Surgery - American Volume*, 96(9):705–711, 2014.

[439] E. Swart, P. Roulette, D. Leas, K.J. Bozic, and M. Karunakar. Orif or arthroplasty for displaced femoral neck fractures in patients younger than 65 years old an economic decision analysis. *Journal of Bone and Joint Surgery - American Volume*, 99(1):65–75, 2017.

[440] E. Swart, E. Vasudeva, E.C. Makhni, W. Macaulay, and K.J. Bozic. Dedicated perioperative hip fracture comanagement programs are cost-effective in high-volume centers: An economic analysis. *Clinical Orthopaedics and Related Research*, 474(1):222–233, 2016.

[441] A.-K.R. Sørensen, L.H. Hammeken, A.H. Qvist, S.L. Jensen, and L.H. Ehlers. Operative treatment of displaced midshaft clavicular fractures is not cost-effective. *Journal of Shoulder and Elbow Surgery*, 29(1):27–35, 2020.

[442] P. Tack, J. Victor, P. Gemmel, and L. Annemans. Do custom 3d-printed revision acetabular implants provide enough value to justify the additional costs? the health-economic comparison of a new porous 3d-printed hip implant for revision arthroplasty of paprosky type 3b acetabular defects and its closest alternative. *Orthopaedics and Traumatology: Surgery and Research*, 107(1), 2021.

[443] S. Takahashi, M. Hoshino, H. Yasuda, H. Terai, K. Hayashi, T. Tsujio, H. Kono, A. Suzuki, K. Tamai, S. Ohyama, H. Toyoda, S. Dohzono, F. Kanematsu, Y. Hori, and H. Nakamura. Cost-effectiveness of balloon kyphoplasty for patients with acute/subacute osteoporotic vertebral fractures in the super-aging japanese society. *Spine*, 44(5):E298–E305, 2019.

[444] T.L. Tan, K. Goswami, Y.A. Fillingham, N. Shohat, A.J. Rondon, and J. Parvizi. Defining treatment success after 2-stage exchange arthroplasty for periprosthetic

joint infection. *Journal of Arthroplasty*, 33(11):3541–3546, 2018.

[445] T.L. Tan, K. Goswami, M.M. Kheir, C. Xu, Q. Wang, and J. Parvizi. Surgical treatment of chronic periprosthetic joint infection: Fate of spacer exchanges. *Journal of Arthroplasty*, 34(9):2085–2090.e1, 2019.

[446] R.J. Taylor and R.S. Taylor. Spinal cord stimulation for failed back surgery syndrome: A decision-analytic model and cost-effectiveness analysis. *International Journal of Technology Assessment in Health Care*, 21(3):351–358, 2005.

[447] G.R. Tennvall and J. Apelqvist. Prevention of diabetes-related foot ulcers and amputations: A cost-utility analysis based on markov model simulations. *Diabetologia*, 44(11):2077–2087, 2001.

[448] R.M. Tikhilov, A.A. Dzhavadov, A.O. Denisov, A.M. Chililov, M.A. Cherkasov, S.S. Bilyk, I.E. Khujanazarov, and I.I. Shubnyakov. Cost-effectiveness analysis of custom-made and serial acetabular components in revision hip arthroplasty. *Genij Ortopedii*, 28(2):234–240, 2022.

[449] T. Tischer, R. Lenz, J. Breinlinger-O'Reilly, and C. Lutter. Cost analysis in shoulder surgery: A systematic review. *Orthopaedic Journal of Sports Medicine*, 8(5), 2020.

[450] M.T. Torchia, D.C. Austin, S.T. Kunkel, K.W. Dwyer, and W.E. Moschetti. Next-generation sequencing vs culture-based methods for diagnosing periprosthetic joint infection after total knee arthroplasty: A cost-effectiveness analysis. *Journal of Arthroplasty*, 34(7):1333–1341, 2019.

[451] M.T. Torchia, I.A. Khan, D.D. Christensen, W.E. Moschetti, and Y.A. Fillingham. Universal screening for malnutrition prior to total knee arthroplasty is cost-effective: A markov analysis. *Journal of Arthroplasty*, 2022.

[452] C.M. Toscano, T.H. Sugita, M.Q.M. Rosa, H.C. Pedrosa, R.S. Rosa, and L.R. Bahia. Annual direct medical costs of diabetic foot disease in brazil: A cost of illness study. *International Journal of Environmental Research and Public Health*, 15(1), 2018.

[453] A.P. Truong, D. Pérez-Prieto, J. Byrnes, J.C. Monllau, and C.J. Vertullo. Vancomycin soaking is highly cost-effective in primary aclr infection prevention: A cost-effectiveness study. *American Journal of Sports Medicine*, 50(4):922–931, 2022.

[454] L.M. Uhler, W.R. Schultz, A.D. Hill, and K.M. Koenig. Health utility of early hemiarthroplasty vs delayed total hip arthroplasty for displaced femoral neck fracture in elderly patients: A markov model. *Journal of Arthroplasty*, 32(5):1434–1438, 2017.

[455] V. Vahdat, J. Griffin, S. Burns, and R. Azghandi. Proactive patient flow redesign for integration of multiple outpatient clinics. pages 2893–2904. Institute of Electrical and Electronics Engineers Inc., 2017.

[456] V. Vahdat, A. Namin, R. Azghandi, and J. Griffin. Improving patient timeliness of care through efficient outpatient clinic layout design using data-driven simulation and optimisation. *Health Systems*, 8(3):162–183, 2019.

[457] C.J.A. van Bergen, O.L. Baur, C.D. Murawski, P. Spennacchio, D.S. Carreira, S.R. Kearns, A.W. Mitchell, H. Pereira, C.J. Pearce, J.D.F. Calder, J. Ackermann, Jr. Adams, S.B., C.L. Andrews, C. Angthong, J.P. Batista, S. Bayer, C. Becher, G.C. Berlet, L.A.T. Boakye, A.J. Brown, R. Buda, G.L. Canata, T.O. Clanton, J. Dahmen, P. D'Hooghe, C.W. DiGiovanni, M.E. Dombrowski, M.C. Drakos, R.D. Ferkel, P.N.F. Ferrao, L.A. Fortier, M. Glazebrook, E. Giza, M. Gomaa, S. Görtz, A.M. Haleem, K. Hamid, L. Hangody, C.P. Hannon, D. Haverkamp, J. Hertel, B. Hintermann, M.V. Hogan, K.J. Hunt, E.T. Hur-

ley, J. Karlsson, J.G. Kennedy, G.M.M.J. Kerkhoffs, H.J. Kim, S.W. Kong, S.A. Labib, K.T.A. Lambers, J.W. Lee, K.B. Lee, J.S. Ling, U.G. Longo, A. Marangon, G. McCollum, P.N. Mittwede, S. Nehrer, P. Niemeyer, II Nunley, J.A., M.J. O'Malley, D.O. Osei-Hwedieh, J. Paul, A. Popchak, M.P. Prado, S.M. Raikin, M.L. Reilingh, B.B. Rothrauff, L.C. Schon, Y. Shimozono, H. Simpson, N.A. Smyth, C.M. Sofka, J.W. Stone, M. Sullivan, M. Takao, Y. Tanaka, D.B. Thordarson, R. Tuan, V. Valderrabano, C.N. van Dijk, P.A.D. van Dijk, F. Vannini, T. Vaseenon, M. Walther, M. Wiewiorski, X. Xu, Y. Yasui, H. Yinghui, I. Yoshimura, A.S.E. Younger, Z. Zhang, and the International Consensus Group on Cartilage Repair of the Ankle. Diagnosis: History, physical examination, imaging, and arthroscopy: Proceedings of the international consensus meeting on cartilage repair of the ankle. *Foot and Ankle International*, 39(1_suppl):3S–8S, 2018.

[458] T. Van Den Wyngaert, S.R. Palli, R.J. Imhoff, and M.T. Hirschmann. Cost-effectiveness of bone spect/ct in painful total knee arthroplasty. *Journal of Nuclear Medicine*, 59(11):1742–1750, 2018.

[459] R.B. Van Der Meer, L.A. Rymaszewski, H. Findlay, and J. Curran. Using or to support the development of an integrated musculo-skeletal service. *Journal of the Operational Research Society*, 56(2):162–172, 2005.

[460] P.A.D. van Dijk, C.D. Murawski, K.J. Hunt, C.L. Andrews, U.G. Longo, G. McCollum, H. Simpson, C.M. Sofka, I. Yoshimura, J. Karlsson, J. Ackermann, Jr. Adams, S.B., C. Angthong, J.P. Batista, O.L. Baur, S. Bayer, C. Becher, G.C. Berlet, L.A.T. Boakye, A.J. Brown, R. Buda, J.D.F. Calder, G.L. Canata, D.S. Carreira, T.O. Clanton, J. Dahmen, P. D'Hooghe, C.W. DiGiovanni, M.E. Dombrowski, M.C. Drakos, R.D. Ferkel, P.N.F. Ferrao, L.A. Fortier, M. Glazebrook, E. Giza, M. Gomaa, S. Görtz, A.M. Haleem, K. Hamid, L. Hangody, C.P. Hannon, D. Haverkamp, J. Hertel, B. Hintermann, M.V. Hogan, E.T. Hurley, S.R. Kearns, J.G. Kennedy, G.M.M.J. Kerkhoffs, H.J. Kim, S.W. Kong, S.A. Labib, K.T.A. Lambers, J.W. Lee, K.B. Lee, J.S. Ling, A. Marangon, A.W. Mitchell, P.N. Mittwede, S. Nehrer, P. Niemeyer, II Nunley, J.A., M.J. O'Malley, D.O. Osei-Hwedieh, J. Paul, C.J. Pearce, H. Pereira, A. Popchak, M.P. Prado, S.M. Raikin, M.L. Reilingh, B.B. Rothrauff, L.C. Schon, Y. Shimozono, N.A. Smyth, P. Spennacchio, J.W. Stone, M. Sullivan, M. Takao, Y. Tanaka, D.B. Thordarson, R. Tuan, V. Valderrabano, C.J.A. van Bergen, C.N. van Dijk, F. Vannini, T. Vaseenon, M. Walther, M. Wiewiorski, X. Xu, Y. Yasui, H. Yinghui, A.S.E. Younger, Z. Zhang, and the International Consensus Group on Cartilage Repair of the Ankle. Post-treatment follow-up, imaging, and outcome scores: Proceedings of the international consensus meeting on cartilage repair of the ankle. *Foot and Ankle International*, 39(1_suppl):68S–73S, 2018.

[461] P. Vavken and R. Dorotka. Economic evaluation of nsaid and radiation to prevent heterotopic ossification after hip surgery. *Archives of Orthopaedic and Trauma Surgery*, 131(9):1309–1315, 2011.

[462] H. Vermue, P. Tack, T. Gryson, and J. Victor. Can robot-assisted total knee arthroplasty be a cost-effective procedure? a markov decision analysis. *Knee*, 29:345–352, 2021.

[463] S. Virk, H.S. Sandhu, and S.N. Khan. Cost effectiveness analysis of graft options in spinal fusion surgery using a markov model. *Journal of Spinal Disorders and Techniques*, 25(7):E204–E210, 2012.

[464] S.S. Virk, J.B. Elder, H.S. Sandhu, and S.N. Khan. The cost effectiveness of polyetheretheketone (peek) cages for anterior cervical discectomy and fusion.

Journal of Spinal Disorders and Techniques, 28(8):E482–E492, 2015.

[465] N. Vochelle. Cost effectiveness of a low-molecular-weight heparin in prolonged prophylaxis against deep vein thrombosis after total hip replacement. *PharmacoEconomics*, 13(1 PART I):81–89, 1998.

[466] J.F.A. Vogel, M. Barkhausen, C.M. Pross, and A. Geissler. Defining minimum volume thresholds to increase quality of care: a new patient-oriented approach using mixed integer programming. *European Journal of Health Economics*, 2022.

[467] B. Walton, K. Meijer, K. Melancon, and M. Hartman. A cost analysis of internal fixation versus nonoperative treatment in adult midshaft clavicle fractures using multiple randomized controlled trials. *Journal of Orthopaedic Trauma*, 29(4):173–180, 2015.

[468] S.-H. Wang, C. Xu, T.L. Tan, K. Goswami, A.M. Cooper, and J. Parvizi. Increased postoperative glucose variability is associated with adverse outcome following two-stage exchange arthroplasty for periprosthetic joint infection. *Journal of Arthroplasty*, 35(5):1368–1373, 2020.

[469] A. Watson, T. Leroux, D. Ogilvie-Harris, M. Nousiainen, P.C. Ferguson, L. Murnahan, and T. Dwyer. Entrustable professional activities in orthopaedics. *JBJS Open Access*, 6(2), 2021.

[470] C.A. Weeks, J.D. Marsh, S.J. MacDonald, S. Graves, and E.M. Vasarhelyi. Patellar resurfacing in total knee arthroplasty: A cost-effectiveness analysis. *Journal of Arthroplasty*, 33(11):3412–3415, 2018.

[471] W. Weerawat, J. Pichitlamken, and P. Subsombat. A generic discrete-event simulation model for outpatient clinics in a large public hospital. *Journal of Healthcare Engineering*, 4(2):285–305, 2013.

[472] A.E. White, R. Chatterji, S.U. Zaman, C.J. Hadley, S.B. Cohen, K.B. Freedman, and C.C. Dodson. Development of a return to play checklist following patellar instability surgery: a delphi-based consensus. *Knee Surgery, Sports Traumatology, Arthroscopy*, 28(3):806–815, 2020.

[473] V.E. Wilde, J.J. Ford, and J.M. McMeeken. Indicators of lumbar zygapophyseal joint pain: Survey of an expert panel with the delphi technique. *Physical Therapy*, 87(10):1348–1361, 2007.

[474] D.M. Williams, A.O. Miller, M.W. Henry, G.H. Westrich, and H.M.K. Ghomrawi. Cost-effectiveness of staphylococcus aureus decolonization strategies in high-risk total joint arthroplasty patients. *Journal of Arthroplasty*, 32(9):S91–S96, 2017.

[475] E.E. Williams, J.N. Katz, V.P. Leifer, J.E. Collins, T. Neogi, L.G. Suter, B. Levy, A. Farid, C.E. Safran-Norton, A.D. Paltiel, and E. Losina. Cost-effectiveness of arthroscopic partial meniscectomy and physical therapy for degenerative meniscal tear. *ACR Open Rheumatology*, 4(10):853–862, 2022.

[476] C.D. Witiw, L.A. Tetreault, F. Smieliauskas, B. Kopjar, E.M. Massicotte, and M.G. Fehlings. Surgery for degenerative cervical myelopathy: a patient-centered quality of life and health economic evaluation. *Spine Journal*, 17(1):15–25, 2017.

[477] A.G.H. Witteveen, C.J. Hofstad, M.J. Breslau, L. Blankevoort, and G.M.M.J. Kerkhoffs. The impact of ankle osteoarthritis. the difference of opinion between patient and orthopedic surgeon. *Foot and Ankle Surgery*, 20(4):241–247, 2014.

[478] C.F. Wolf, N.Y. Gu, J.N. Doctor, P.A. Manner, and S.S. Leopold. Comparison of one and two-stage revision of total hip arthroplasty complicated by infection a markov expected-utility decision analysis. *Journal of Bone and Joint Surgery - Series A*, 93(7):631–639, 2011.

[479] S.E. Wolowacz, N.S. Roskell, F. Maciver, S.M. Beard, P.A. Robinson, J.M.

Plumb, G. Dolan, and I.J. Brenkel. Economic evaluation of dabigatran etexilate for the prevention of venous thromboembolism after total knee and hip replacement surgery. *Clinical Therapeutics*, 31(1):194–212, 2009.

[480] C.K.H. Wong, J.P.Y. Cheung, P.W.H. Cheung, C.L.K. Lam, and K.M.C. Cheung. Traditional growing rod versus magnetically controlled growing rod for treatment of early onset scoliosis: Cost analysis from implantation till skeletal maturity. *Journal of Orthopaedic Surgery*, 25(2):1–10, 2017.

[481] C.H. Wu, C.F. Gray, and G.-C. Lee. Arthrodesis should be strongly considered after failed two-stage reimplantation tka. *Clinical Orthopaedics and Related Research*, 472(11):3295–3304, 2014.

[482] M. Xu, D.S. Garbuz, L. Kuramoto, and B. Sobolev. Classifying health-related quality of life outcomes of total hip arthroplasty. *BMC Musculoskeletal Disorders*, 6, 2005.

[483] M. Yaghoubi, M. Moradi-Lakeh, M. Moradi-Joo, V. Rahimi-Movaghar, N. Zamani, and A. Naghibzadeh-Tahami. The cost effectiveness of dynamic and static interspinous spacer for lumbar spinal stenosis compared with laminectomy. *Medical Journal of the Islamic Republic of Iran*, 30(1), 2016.

[484] X. Yan, X. Gu, L. Zhou, H. Lin, and B. Wu. Cost effectiveness of apixaban and enoxaparin for the prevention of venous thromboembolism after total knee replacement in china. *Clinical Drug Investigation*, 36(12):1001–1010, 2016.

[485] S.S. Yeap, S.R. Abu Amin, H. Baharuddin, K.C. Koh, J.K. Lee, V.K.M. Lee, N.H. Mohamad Yahaya, C.C. Tai, and M.P. Tan. A malaysian delphi consensus on managing knee osteoarthritis. *BMC Musculoskeletal Disorders*, 22(1), 2021.

[486] T.M. Yong, D.C. Austin, I.B. Molloy, S.T. Kunkel, D.S. Jevsevar, and I.L. Gitajn. Screw fixation versus hemiarthroplasty for nondisplaced femoral neck fractures in the elderly: A cost-effectiveness analysis. *Journal of Orthopaedic Trauma*, 34(7):348–355, 2020.

[487] R. You, J. Liu, L. Ke, M. Wan, Y. Zhang, G. Yu, and T. Mori. Cost-effectiveness of sequential denosumab/zoledronic acid compared with zoledronic acid monotherapy for postmenopausal osteoporotic women in china. *Frontiers in Pharmacology*, 13, 2022.

[488] R. You, J. Liu, L. Ke, G. Yu, Y. Zhang, and T. Mori. Cost-effectiveness of sequential teriparatide/zoledronic acid compared with zoledronic acid monotherapy for postmenopausal osteoporotic women in china. *Frontiers in Public Health*, 10, 2022.

[489] B.J. Yun, M.G. Myriam Hunink, A.M. Prabhakar, M. Heng, S.W. Liu, R. Qudsi, and A.S. Raja. Diagnostic imaging strategies for occult hip fractures: A decision and cost–effectiveness analysis. *Academic Emergency Medicine*, 23(10):1161–1169, 2016.

[490] L. Zang, N. Fan, Y. Hai, S.B. Lu, Q.J. Su, J.C. Yang, P. Du, and Y.J. Gao. Using the modified delphi method to establish a new chinese clinical consensus of the treatments for cervical radiculopathy. *European Spine Journal*, 24(6):1116–1126, 2015.

[491] P. Zangger and A. Detsky. Computer-assisted decision analysis in orthopedics: Resurfacing the patella in total knee arthroplasty as an example. *Journal of Arthroplasty*, 15(3):283–288, 2000.

[492] N.J. Zarkadis, E.D. Eisenstein, N.A. Kusnezov, J.C. Dunn, and J.A. Blair. Open reduction–internal fixation versus intramedullary nailing for humeral shaft fractures: an expected value decision analysis. *Journal of Shoulder and Elbow Surgery*, 27(2):204–210, 2018.

[493] Z. Zeng, X. Xie, H. Menaker, S.G. Sanford-Ring, and J. Li. Performance evaluation of operating room schedules in orthopedic surgery. *Flexible Services and Manufacturing Journal*, 30(1-2):198–223, 2018.

[494] Z. Zhang, Q. Wu, L. Zeng, and S. Wang. Modeling-based assessment of 3d printing-enabled meniscus transplantation. *Healthcare (Switzerland)*, 7(2), 2019.

[495] M. Zhou, G.G. Loke, C. Bandi, Z.Q.G. Liau, and W. Wang. Intraday scheduling with patient re-entries and variability in behaviours. *Manufacturing and Service Operations Management*, 24(1):561–579, 2022.

[496] T. Zhuang, L.M. Shapiro, D.F. Amanatullah, W.J. Maloney, and R.N. Kamal. Costs and benefits of routine hemoglobin a1c screening prior to total joint arthroplasty: A cost-benefit analysis. *Current Orthopaedic Practice*, 33(4):338–346, 2022.

[497] T. Zhuang, S. Wong, R. Aoki, E. Zeng, S. Ku, and R.N. Kamal. A cost-effectiveness analysis of corticosteroid injections and open surgical release for trigger finger. *Journal of Hand Surgery*, 45(7):597–609.e7, 2020.

[498] S. Zindel, S. Stock, D. Müller, and B. Stollenwerk. A multi-perspective cost-effectiveness analysis comparing rivaroxaban with enoxaparin sodium for thromboprophylaxis after total hip and knee replacement in the german healthcare setting. *BMC Health Services Research*, 12(1), 2012.

[499] B.M. Zmistowski, Y.A. Fillingham, H.I. Salmons, D.T. Ward, R.P. Good, and J.H. Lonner. Routine patellar resurfacing during total knee arthroplasty is not cost-effective in patients without patellar arthritis. *Journal of Arthroplasty*, 34(9):1963–1968, 2019.

[500] C.K. Zogg, J.R. Falvey, J.B. Dimick, A.H. Haider, K.A. Davis, and J.N. Grauer. Changes in discharge to rehabilitation: Potential unintended consequences of medicare total hip arthroplasty/total knee arthroplasty bundled payments, should they be implemented on a nationwide scale? *Journal of Arthroplasty*, 34(6):1058–1065.e4, 2019.
